# Supplementary material for: Mutant ACVR1 Arrests Glial Cell Differentiation to Drive Tumorigenesis in Pediatric Gliomas
Source: Cancer Cell. 2020 Mar 16;37(3):308–323.e12. doi: 10.1016/j.ccell.2020.02.002 (PMC7105820; doi:10.1016/j.ccell.2020.02.002)
Supplement: Document S1. Figures S1–S7 and Tables S2 and S3 [file mmc1.pdf]

Cancer Cell, Volume *37*

Supplemental Information

Mutant *ACVR1* Arrests Glial Cell Differentiation to Drive Tumorigenesis in Pediatric Gliomas

Jerome Fortin, Ruxiao Tian, Ida Zarrabi, Graham Hill, Eleanor Williams, Gonzalo Sanchez-Duffhues, Midory Thorikay, Parameswaran Ramachandran, Robert Siddaway, Jong Fu Wong, Annette Wu, Lorraine N. Apuzzo, Jillian Haight, Annick You-Ten, Bryan E. Snow, Andrew Wakeham, David J. Goldhamer, Daniel Schramek, Alex N. Bullock, Peter ten Dijke, Cynthia Hawkins, and Tak W. Mak

A

$Acvr1^{floxG328V/+}$ X *EIIa-Cre*

Genotype	# alive after birth
$Acvr1^{+/+}$	19
$Acvr1^{floxG328V/+}$	17
$Acvr1^{+/+}$; EIIa-Cre	16
$Acvr1^{floxG328V/+}$; EIIa-Cre	0

B

$Acvr1^{floxG328V/+}$; EIIa-Cre

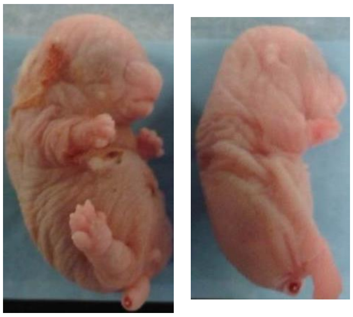

C

Postnatal day 6

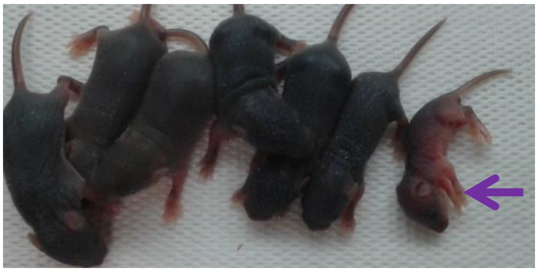

Figure S1, related to Figure 1. Characterization of the *Acvr1*^{floxG328V} allele and effects of the *Acvr1*^{G328V} mutation in glial cells

(A) Table showing the number of viable animals from various genotypes after birth, obtained from a cross of *Acvr1*$^{floxG328V/+}$ mice with *EIIa-Cre* mice.

(B) Photographs of two stillborn *Acvr1*$^{floxG328V/+}$*; EIIa-Cre* pups.

(C) Photograph of a litter from an *Acvr1*$^{floxG328V/+}$ X *Olig2*$^{Cre/+}$ intercross at postnatal day 6. The arrow indicates a runt *Acvr1*$^{floxG328V/+}$*;Olig2*$^{Cre/+}$ pup that did not survive until weaning.

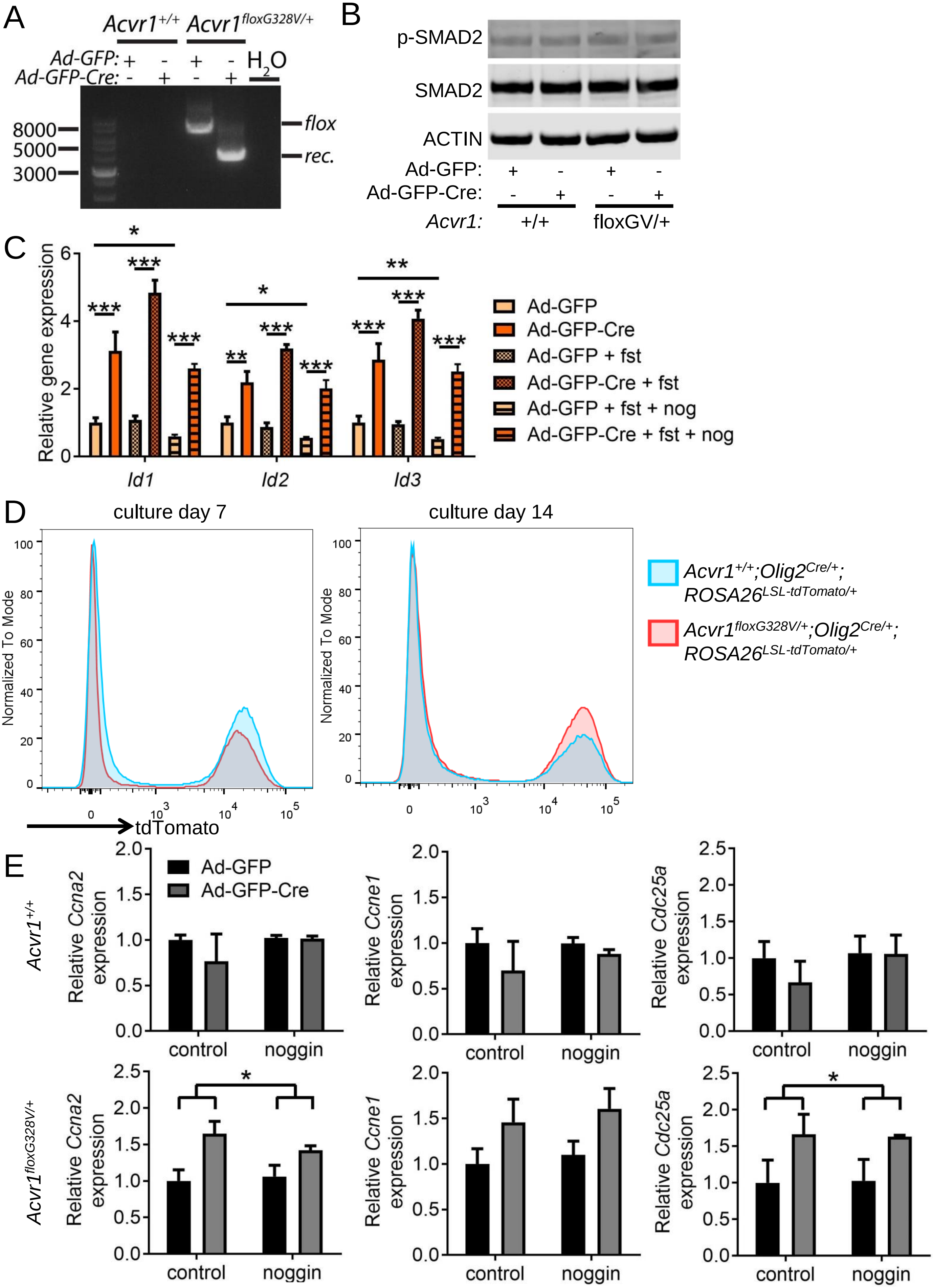
A
Acvr1+/+ Acvr1floxG328V/+
Ad-GFP: + - + - H2O
Ad-GFP-Cre: - + - +
8000
5000
3000
flox
rec.
B
p-SMAD2
SMAD2
ACTIN
Ad-GFP: + - + -
Ad-GFP-Cre: - + - +
Acvr1: +/+ floxGV/+
C
Relative gene expression
Id1
Id2
Id3
Ad-GFP
Ad-GFP-Cre
Ad-GFP + fst
Ad-GFP-Cre + fst
Ad-GFP + fst + nog
Ad-GFP-Cre + fst + nog
D
culture day 7
culture day 14
Normalized To Mode
tdTomato
Acvr1+/+;Olig2Cre/+;ROSA26LSL-tdTomato/+
Acvr1floxG328V/+;Olig2Cre/+;ROSA26LSL-tdTomato/+
E
Acvr1+/+
Acvr1floxG328V/+
Ad-GFP
Ad-GFP-Cre
Relative Ccna2 expression
Relative Ccne1 expression
Relative Cdc25a expression
control
noggin

F

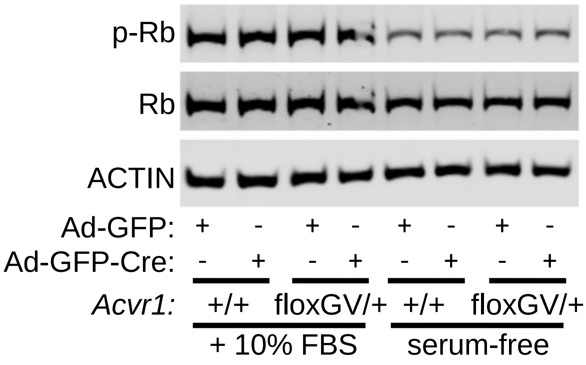
p-Rb
Rb
ACTIN
Ad-GFP: + - + - + - + -
Ad-GFP-Cre: - + - + - + - +
Acvr1: +/+ floxGV/+ +/+ floxGV/+
+ 10% FBS
serum-free

G

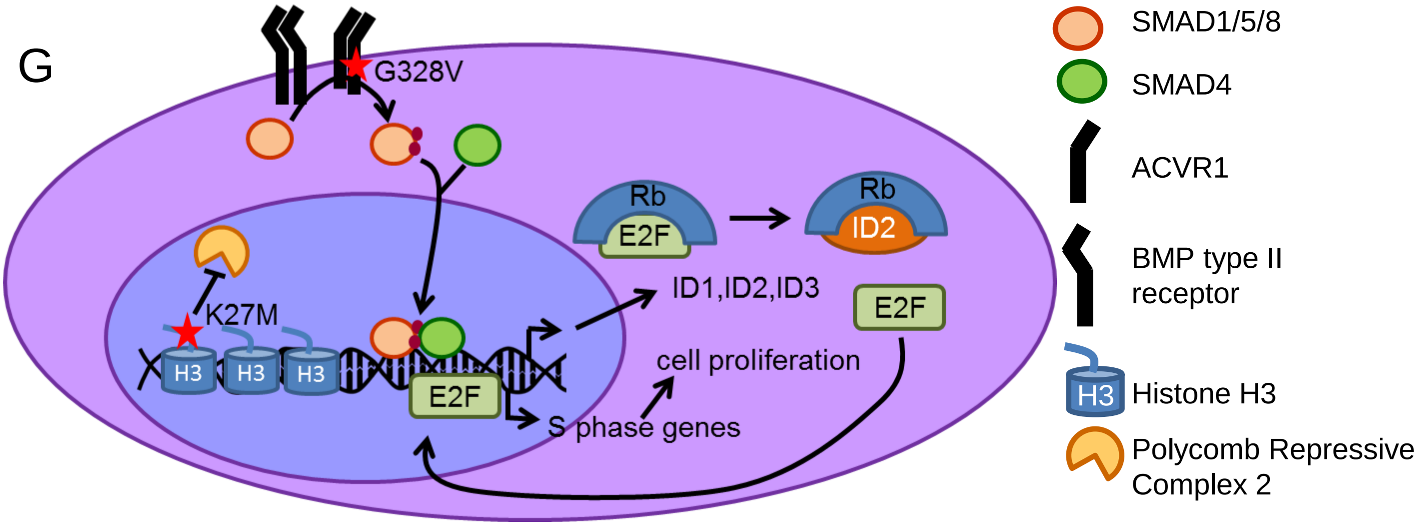
G328V
K27M
H3
H3
H3
E2F
Rb
E2F
Rb
ID2
ID1,ID2,ID3
E2F
cell proliferation
S phase genes
SMAD1/5/8
SMAD4
ACVR1
BMP type II receptor
H3
Histone H3
Polycomb Repressive Complex 2

Figure S2, related to Figure 2. ***Acvr1^{G328V}* enhances glial cell proliferation**

(A) Analysis of genomic DNA by PCR with primers amplifying the unrecombined (flox) and recombined (rec.) *Acvr1$^{floxG328V/+}$* allele, extracted from primary brainstem glial cell cultures prepared from *Acvr1$^{+/+}$* or *Acvr1$^{floxG328V/+}$* pups and transduced with Ad-GFP or Ad-GFP-Cre. DNA markers are shown on the right lane.

(B) Western blot of whole cell lysates from primary glial cells prepared from *Acvr1$^{+/+}$* or *Acvr1$^{floxG328V/+}$* (floxGV) pups and transduced with Ad-GFP or Ad-GFP-Cre, probed with p-SMA2 (top), SMAD2 (middle), and actin (bottom) antibodies.

(C) Expression of *Id1*, *Id2*, and *Id3*, normalized to the expression of *Rpl19*, measured by qPCR on cDNA prepared from primary brainstem glial cells derived from *Acvr1$^{floxG328V/+}$* pups, transduced with *Ad-GFP* or *Ad-GFP-Cre*, and treated or not with 100 ng/mL follistatin (fst) and/or 100 ng/mL noggin (Nog). n=4 independent cultures from 4 pups per genotype. *:$p<0.05$; **:$p<0.01$; ***:$p<0.001$, assessed by repeated-measures ANOVA, assessed by repeated-measures ANOVA with Sidak multiple comparisons tests.

(D) Flow cytometry histograms showing tdTomato-positive and tdTomato-negative cells in primary brainstem glial cell cultures prepared from *Acvr1$^{+/+}$;Olig2$^{Cre/+}$;ROSA26$^{LSL\text{-}tdTomato/+}$* (blue trace) or *Acvr1$^{floxG328V/+}$;Olig2$^{Cre/+}$;ROSA26$^{LSL\text{-}tdTomato/+}$* (red trace) pups at culture day 7 (left) and 14 (right). To adjust for different cell counts, data were normalized to the highest point on the histogram for in each sample (normalized to mode).

(E) Expression of *Ccna2* (left), *Ccne1* (middle) and *Cdc25a* (right), normalized to the expression of *Rpl19*, measured by qPCR on cDNA prepared from brainstem glial cultures derived from *Acvr1$^{+/+}$* (top) or *Acvr1flox$^{G328V/+}$* (bottom), and transduced with Ad-GFP (black bars) or Ad-GFP-Cre (grey bars). In all panels, *:$p<0.05$, assessed by repeated-measures ANOVA with Tukey multiple comparisons tests.

(F) Western blot of whole cell lysates from primary glial cells prepared from *Acvr1$^{+/+}$* (+/+) or *Acvr1$^{floxG328V/+}$* (floxGV) pups, transduced with Ad-GFP or Ad-GFP-Cre, and cultured in the presence of 10% fetal bovine serum (FBS) or under serum-free conditions, probed with phospho-Rb (p-Rb) (top), Rb (middle), and actin (bottom) antibodies.

(G) Model of the cellular effects of the *Acvr1^{G328V}* mutation on BMP target gene expression and cell proliferation, inferred from the data shown in this paper. *Acvr1^{G328V}* increases the expression of *Id1*, *Id2*, and *Id3*, and drive cell cycle progression in the absence of growth factors through a mechanism involving enhanced E2F-dependent transcriptional activity, likely as a result of increased ID2 levels.

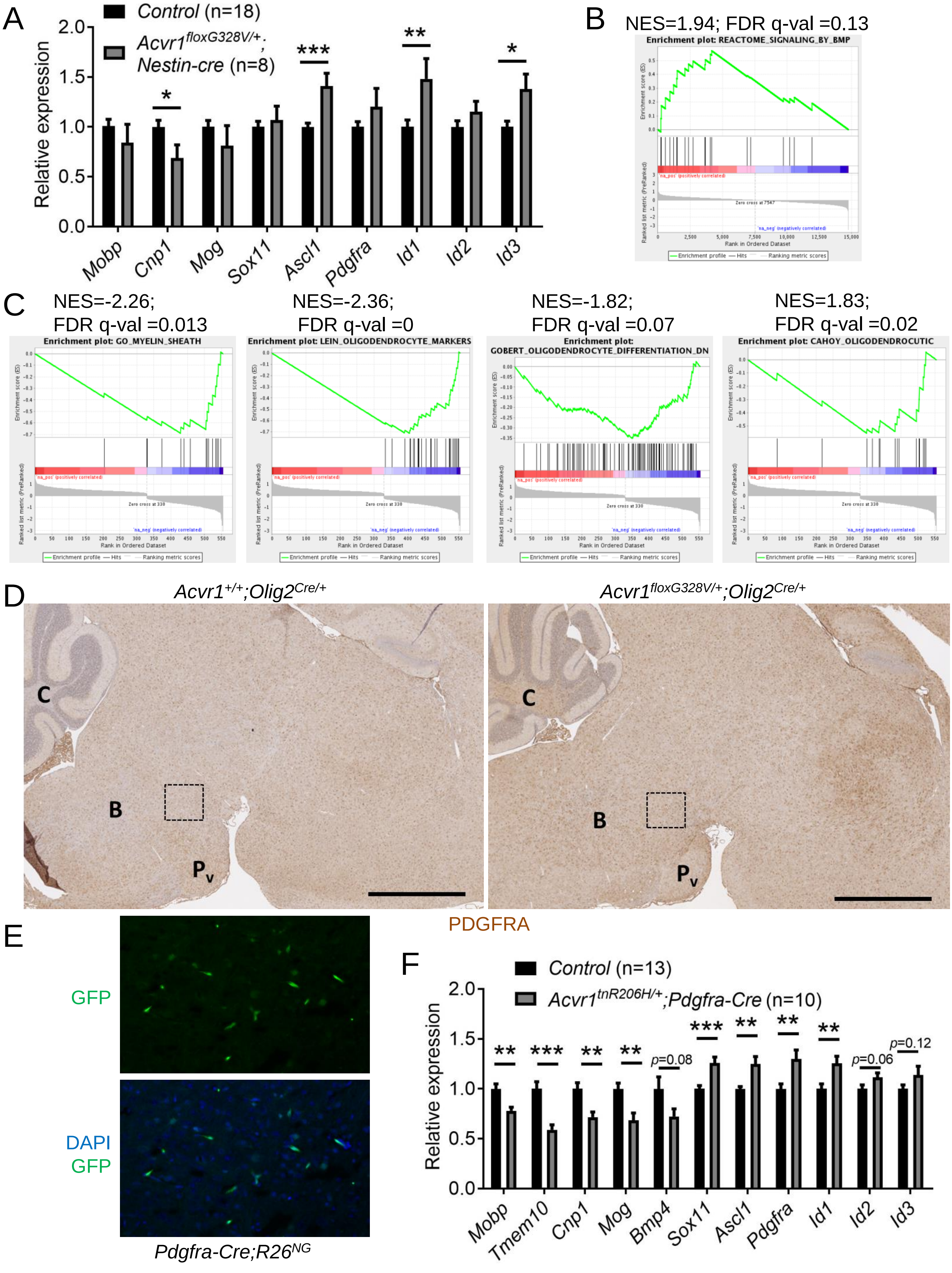
A
Control (n=18)
Acvr1floxG328V/+;
Nestin-cre (n=8)
Relative expression
2.0
1.5
1.0
0.5
0.0
*
***
**
*
Mobp
Cnp1
Mog
Sox11
Ascl1
Pdgfra
Id1
Id2
Id3
B
NES=1.94; FDR q-val =0.13
Enrichment plot: REACTOME_SIGNALING_BY_BMP
Zero cross at 7547
Rank in Ordered Dataset
Enrichment profile — Hits — Ranking metric scores
C
NES=-2.26;
FDR q-val =0.013
Enrichment plot: GO_MYELIN_SHEATH
NES=-2.36;
FDR q-val =0
Enrichment plot: LEIN_OLIGODENDROCYTE_MARKERS
NES=-1.82;
FDR q-val =0.07
Enrichment plot:
GOBERT_OLIGODENDROCYTE_DIFFERENTIATION_DN
NES=1.83;
FDR q-val =0.02
Enrichment plot: CAHOY_OLIGODENDROCUTIC
Zero cross at 330
Rank in Ordered Dataset
Enrichment profile — Hits — Ranking metric scores
D
Acvr1+/+;Olig2Cre/+
Acvr1floxG328V/+;Olig2Cre/+
C
B
Pv
PDGFRA
E
GFP
DAPI
GFP
Pdgfra-Cre;R26NG
F
Control (n=13)
Acvr1tnR206H/+;Pdgfra-Cre (n=10)
Relative expression
2.0
1.5
1.0
0.5
0.0
**
***
**
**
p=0.08
***
**
**
**
p=0.06
p=0.12
Mobp
Tmem10
Cnp1
Mog
Bmp4
Sox11
Ascl1
Pdgfra
Id1
Id2
Id3

Figure S3, related to Figure 3. ***Acvr1**^{G328V}* **induces differentiation arrest of oligodendroglial lineage cells and PDGFRA upregulation**

(A) Expression of selected genes, normalized to the expression of *Rpl19*, measured by qPCR on cDNA prepared from the brainstem tissue of control (black bars – a mix of *Acvr1*$^{+/+}$, *Acvr1*$^{floxG328V/+}$, and *Acvr1*$^{+/+}$*;Nestin-Cre*) and *Acvr1*$^{floxG328V/+}$*;Nestin-Cre* (grey bars) postnatal day 7 pups.

(B) Gene Set Enrichment Analysis (GSEA) showing significantly altered BMP signaling pathway (FDR q value <0.25) in *Acvr1*$^{floxG328V/+}$*;Olig2*$^{Cre/+}$ P7 pups, compared with their *Acvr1*$^{+/+}$*;Olig2*$^{Cre/+}$ littermates, using a pre-ranked list of all genes with FPKM>10. The Normalized Enrichment Score (NES) and FDR q value are indicated on top of the plot.

(C) Gene Set Enrichment Analysis (GSEA) showing significantly altered pathways (FDR q value <0.25), a pre-ranked list of genes differentially expressed in *Acvr1*$^{floxG328V/+}$*;Olig2*$^{Cre/+}$ P7 pups, compared with their *Acvr1*$^{+/+}$*;Olig2*$^{Cre/+}$ littermates, with adjusted p values lower than 0.1. The Normalized Enrichment Score (NES) and FDR q values are indicated on top of each plot.

(D) Representative immunohistochemistry images showing PDGFRA protein expression in sagittal brain sections from *Acvr1*$^{+/+}$*;Olig2*$^{Cre/+}$ (left) and *Acvr1*$^{floxG328V/+}$*;Olig2*$^{Cre/+}$ (right) postnatal day 14 mice. Higher magnifications of the boxed areas are shown in Figure 3D. C: Cerebellum. B: Brainstem. P_V: ventral pons. Scale bars: 1 mm.

(E) GFP (top) and combined GFP and DAPI (bottom) fluorescence microscopy images of a sagittal brainstem section for a postnatal day 7 *Pdgfra-Cre;R26*^{NG} mouse.

(F) Expression of selected genes, normalized to the expression of *Rpl19*, measured by qPCR on cDNA prepared from the brainstem tissue of control (black bars – a mix of *Acvr1*$^{+/+}$ and *Acvr1*$^{+/+}$*;Pdgfra-Cre*) and *Acvr1*$^{tnR206H/+}$*;Pdgfra-Cre* (grey bars) postnatal day 7 pups.

In (A) and (F), *:p<0.05; **:p<0.01; ***:p<0.001, assessed by unpaired t-test.

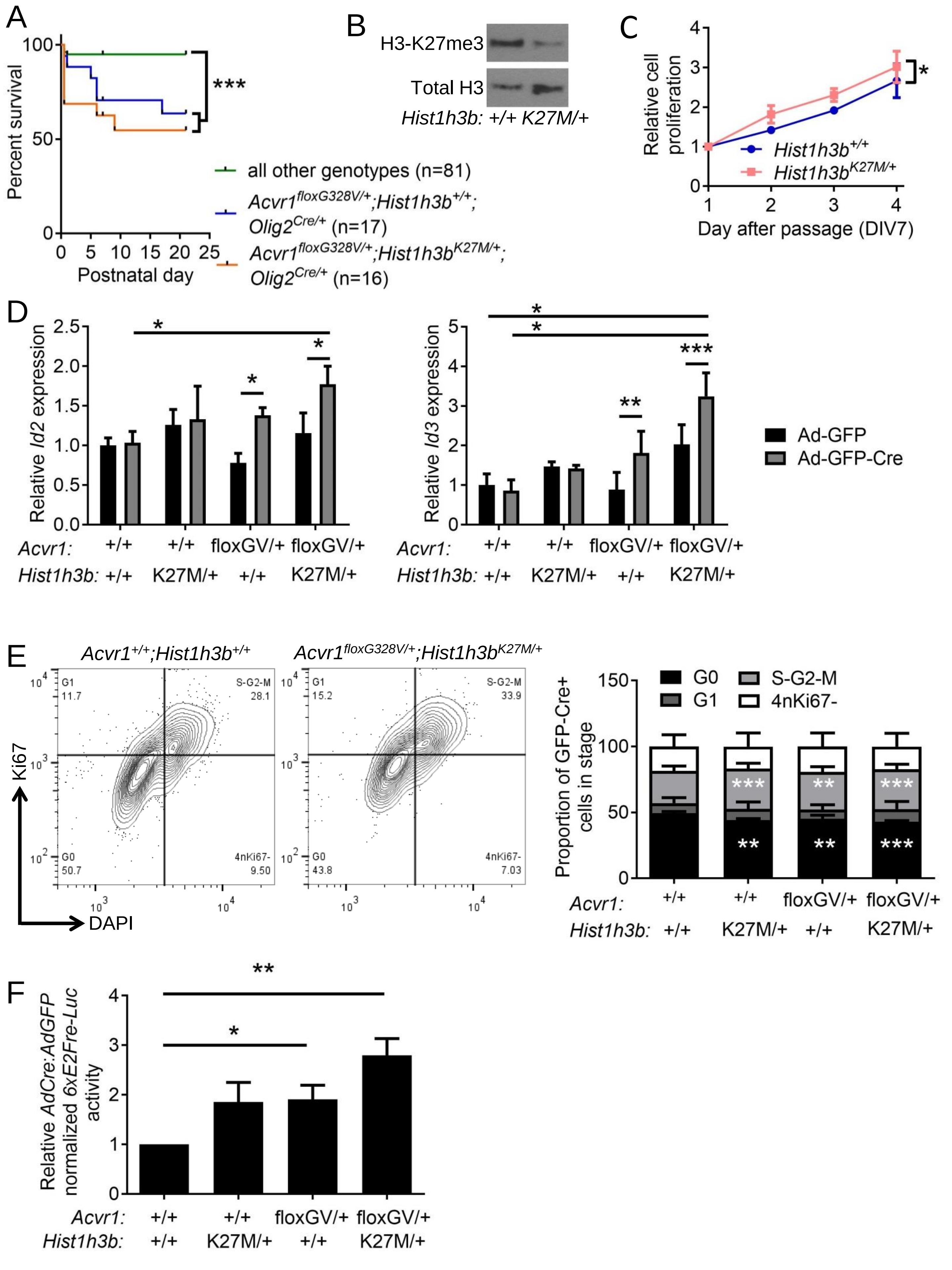

A
Percent survival
Postnatal day
***
all other genotypes (n=81)
Acvr1floxG328V/+;Hist1h3b+/+;Olig2Cre/+ (n=17)
Acvr1floxG328V/+;Hist1h3bK27M/+;Olig2Cre/+ (n=16)
B
H3-K27me3
Total H3
Hist1h3b: +/+ K27M/+
C
Relative cell proliferation
Hist1h3b+/+
Hist1h3bK27M/+
Day after passage (DIV7)
D
Relative Id2 expression
Relative Id3 expression
Ad-GFP
Ad-GFP-Cre
Acvr1: +/+ +/+ floxGV/+ floxGV/+
Hist1h3b: +/+ K27M/+ +/+ K27M/+
E
Acvr1+/+;Hist1h3b+/+
Acvr1floxG328V/+;Hist1h3bK27M/+
G1 11.7
S-G2-M 28.1
G0 50.7
4nKi67- 9.50
G1 15.2
S-G2-M 33.9
G0 43.8
4nKi67- 7.03
Ki67
DAPI
G0
G1
S-G2-M
4nKi67-
Proportion of GFP-Cre+ cells in stage
F
Relative AdCre:AdGFP normalized 6xE2Fre-Luc activity

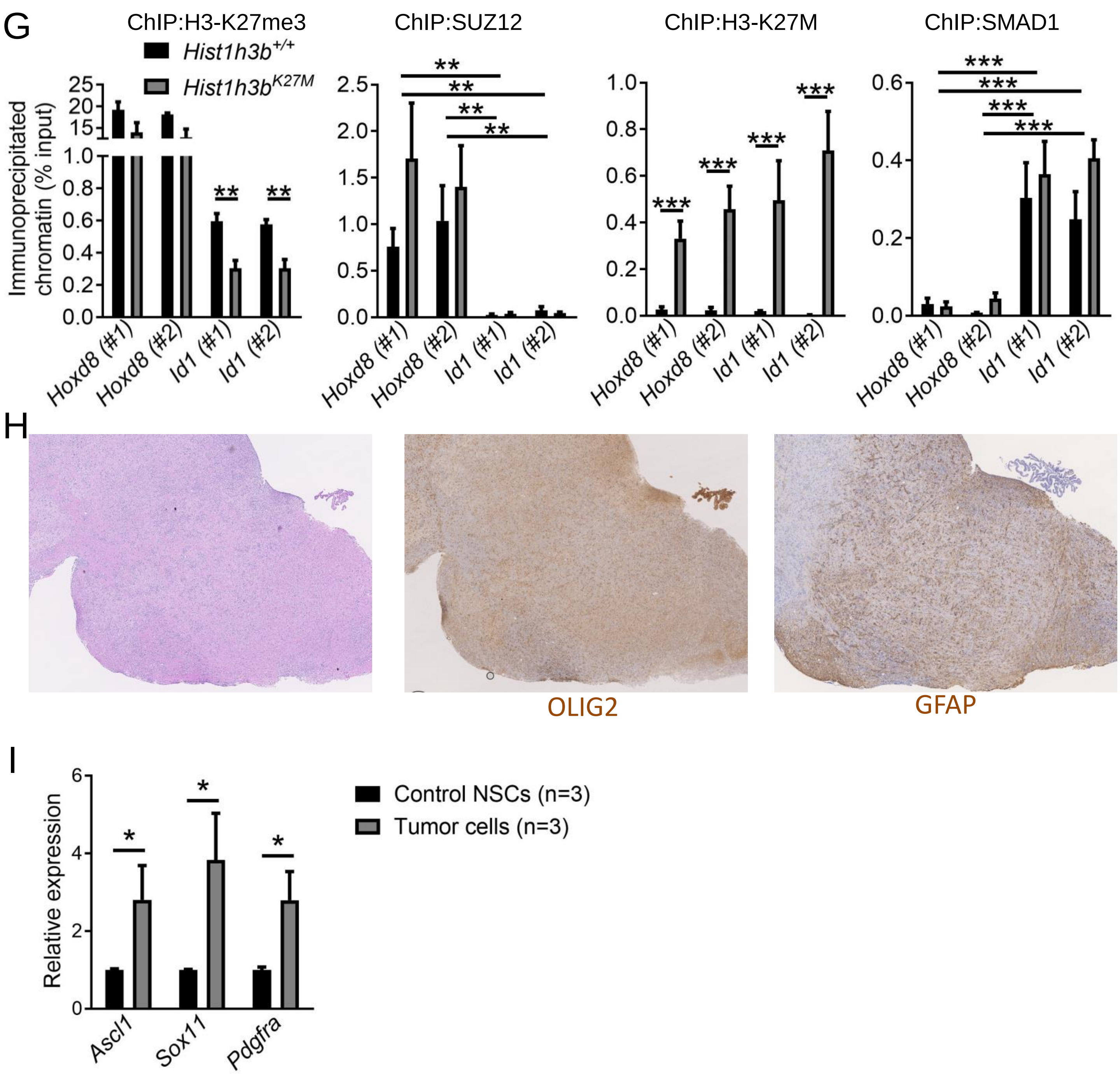
G
ChIP:H3-K27me3
ChIP:SUZ12
ChIP:H3-K27M
ChIP:SMAD1
Hist1h3b+/+
Hist1h3bK27M
Immunoprecipitated chromatin (% input)
Hoxd8 (#1)
Hoxd8 (#2)
Id1 (#1)
Id1 (#2)
H
OLIG2
GFAP
I
Relative expression
Control NSCs (n=3)
Tumor cells (n=3)
Ascl1
Sox11
Pdgfra

Figure S4, related to Figure 4. ***Acvr1*^{G328V} and *Hist1h3b*^{K27M} cooperatively stimulate gene expression and glial cell proliferation**

(A) Survival curves of *Acvr1*$^{floxG328V/+}$;*Hist1h3b*$^{K27M/+}$;*Olig2*$^{Cre/+}$ mice (orange curve), *Acvr1*$^{floxG328V/+}$;*Olig2*$^{Cre/+}$ mice (blue curve), and their control littermates (green curve), within the first three postnatal weeks.

(B) Western blot of whole cell lysates from primary brainstem glial cells prepared from *Hist1h3b*$^{+/+}$ or *Hist1h3b*^{K27M} pups, probed with H3-K27me3 (top), or total-H3 (bottom) antibodies.

(C) Relative cell proliferation, measured by the PrestoBlue assay, in primary brainstem glial cells prepared from *Hist1h3b*$^{+/+}$ (blue line) and *Hist1h3b*$^{K27M/+}$ (pink line) pups. n=5 independent experiments.

(D) Expression of *Id2* (left), and *Id3* (right), normalized to the expression of *Rpl19*, measured by qPCR on cDNA prepared from primary glial cells derived from pups carrying a combination of *Acvr1*$^{+/+}$ or *Acvr1*$^{floxG328V/+}$, and *Hist1h3b*$^{+/+}$ or *Hist1h3b*$^{K27M/+}$ alleles as indicated under the graphs (floxGV indicates the *Acvr1*^{floxG328V} allele), transduced with Ad-GFP (black bars) or Ad-GFP-Cre (grey bars), and treated with 100 ng/mL noggin. n=3 experiments from 3 independent litters.

(E) Left: representative flow cytometry plots depicting GFP-positive cells from Ad-GFP-Cre-transduced primary brainstem glial cell cultures prepared from *Acvr1*$^{+/+}$;*Hist1h3b*$^{+/+}$ and *Acvr1*$^{floxG328V/+}$;*Hist1h3b*$^{K27M/+}$ pups and stained with Ki67 antibodies and DAPI. The gating strategy to identify cells in the G0, G1, S-G2-M, and undefined ("4nKi67-") phases of the cell cycles is shown. Right: quantification of the percentage of GFP-positive cells in each cell cycle stage in Ad-GFP-Cre-transduced primary brainstem cell cultures derived from pups carrying a combination of *Acvr1*$^{+/+}$ or *Acvr1*$^{floxG328V/+}$, and *Hist1h3b*$^{+/+}$ or *Hist1h3b*$^{K27M/+}$ alleles as indicated under the graphs (floxGV indicates the *Acvr1*^{floxG328V} allele). n= 2 experiments from 2 independent litters.

(F) Relative reporter activity, measured by luminescence activity, in lysates from primary brainstem glial cells prepared from pups carrying a combination of *Acvr1*$^{+/+}$ or *Acvr1*$^{floxG328V/+}$, and *Hist1h3b*$^{+/+}$ or *Hist1h3b*$^{K27M/+}$ alleles as indicated under the graphs (floxGV indicates the *Acvr1*^{floxG328V} allele), transduced with Ad-GFP or Ad-GFP-Cre, and transfected with Renilla-Luc (to control for differences in transfection efficiency) and the 6xE2F-Luc reporter. Relative reporter activity was measured as a ratio of luminescence in lysates from cells transduced with Ad-GFP-Cre compared with Ad-GFP. n=4 experiments from 4 independent litters. Data from the "*Acvr1*$^{+/+}$;*Hist1h3b*$^{+/+}$" and "*Acvr1*$^{floxG328V/+}$;*Hist1h3b*$^{+/+}$" conditions are also shown by themselves in Figure 2F, and included here for comparison purposes.

(G) Occupancy of two regions of the *Hoxd8* promoter and of the *Id1* promoter by H3-K27me3 (left), SUZ12 (second from left), H3-K27M (third from left), and SMAD1 (right), measured by chromatin immunoprecipitation (ChIP) in primary glial cells prepared from *Hist1h3b*$^{+/+}$ (black bars) or *Hist1h3b*^{K27M} (grey bars) pups, expressed as a percentage of the input chromatin. For all the ChIP experiments, data were pooled from at least 3 independent primary glial cultures, prepared from separate animals, for each genotype.

(H) Histological sections showing an example of a high-grade diffuse glioma with brainstem involvement in an *Acvr1*$^{floxG328V/+}$*; Pik3ca*$^{floxH1047R/+}$*; Olig2*$^{Cre/+}$ mouse, stained with hematoxylin and eosin (left), and immunohistochemistry for OLIG2 (middle), and GFAP (right). Scale bars: 1mm.

(I) Expression of *Ascl1*, *Sox11*, and *Pdgfra*, normalized to the expression of *Rpl19*, measured by qPCR on cDNA prepared from cell lines derived from *Acvr1*^{floxG328V}; *Hist1h3b*^{K27M}; *Pik3ca*^{floxH1047R}; *Olig2*^{Cre} tumors, and from neural stem cells derived from normal brains, cultured in the same conditions.

In all panels, *:$p<0.05$; **:$p<0.01$; ***:$p<0.001$, assessed by Mantel-Cox test (A), linear regression analysis (C), repeated-measures ANOVA with Tukey (D-F) multiple-comparisons tests, two-way ANOVA with Sidak multiple comparisons tests (G), or t-tests (I).

A

	SOX11 log$_2$FC	*SOX11* Adj. p value	*ASCL1* log$_2$FC	*ASCL1* Adj. p value
Normal brain vs. DIPG	2	<0.0001	1.586	<0.0001
Normal brain vs. *ACVR1*-WT	2.006	<0.0001	1.489	0.0004
Normal brain vs. *ACVR1*-MUT	1.962	0.021	2.047	0.0002
ACVR1-WT vs. *ACVR1*-MUT	-0.044	>0.9999	0.558	0.4007

Spearman	*SOX11-ASCL1* r	*SOX11-ASCL1* p value
Overall	0.807	8.93E-13
Normal	0.461	0.0625
DIPG	0.666	1.68E-05
ACVR1-WT	0.726	8.14E-06
ACVR1-Mut	0.7	0.233

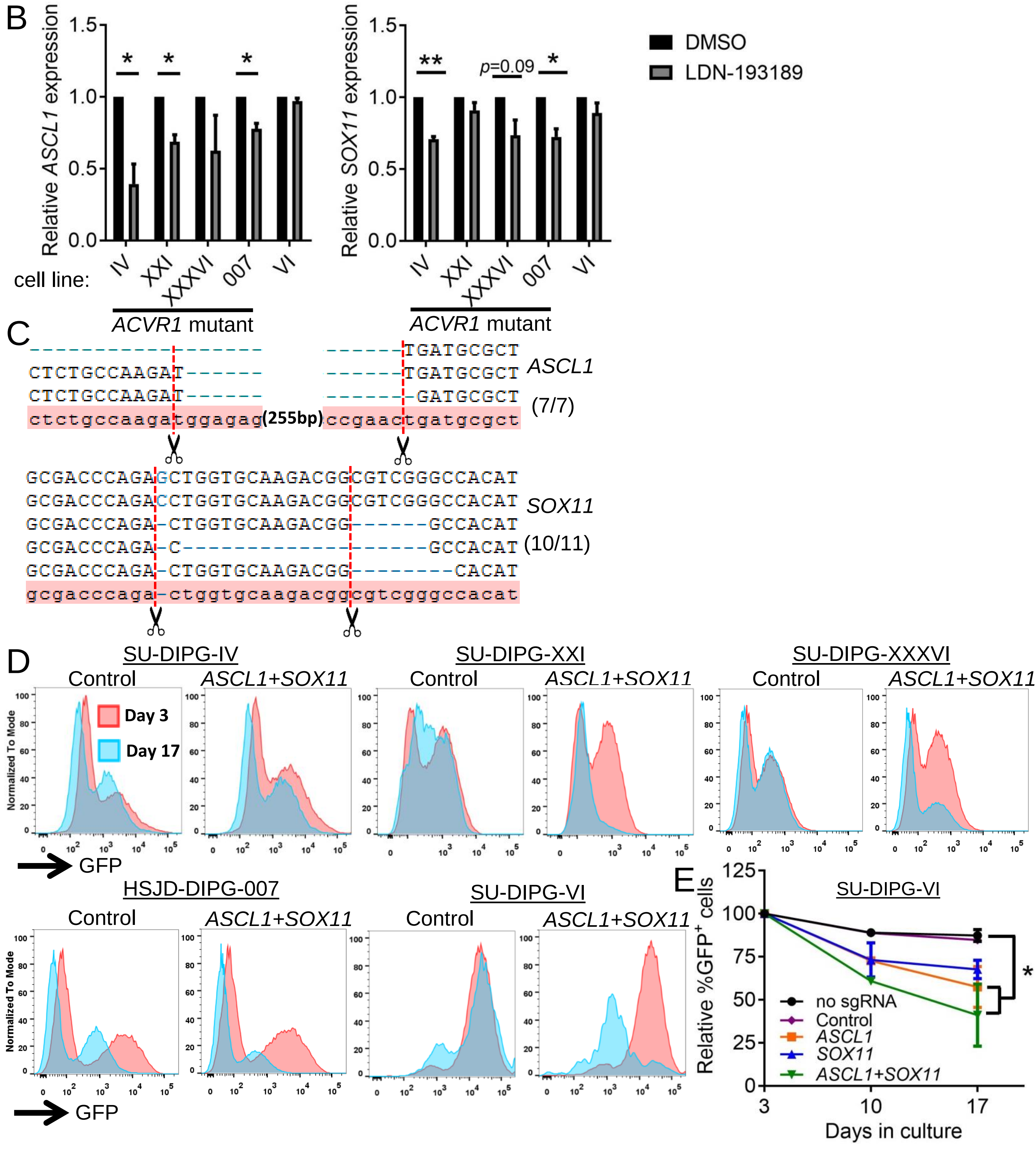

Figure S5, related to Figure 5. ASCL1 and SOX11 control DIPG cell fitness

(A) Left: summary statistic comparing the expression of *ASCL1* and *SOX11* in normal brain and DIPG samples, or only those DIPG samples that are wild-type (WT) or mutant (MUT) for *ACVR1*, assessed by ANOVA with Dunn's multiple comparisons tests. Right: Spearman analysis of the correlation between *ASCL1* and *SOX11* expression in the same samples.

(B) Expression of *ASCL1* (left) and SOX11 (right), normalized to the expression of *RPL19*, measured by qPCR on cDNA prepared from *ACVR1* mutant DIPG cell lines (SU-DIPG-IV, SU-DIPG-XXI, SU-DIPG-XXXVI, HSJD-DIPG-007, or *ACVR1* wild-type DIPG cells (SU-DIPG-VI), treated with 1 µM LDN-193189 (grey bars), or DMSO (black bars) for 24 hours.

(C) Alignment to the reference genome (highlighted in red) of sequencing reads from randomly selected clones generated from PCR-amplified fragments of the genomic regions edited by sgRNAs targeting *ASCL1* (top) and *SOX11* (bottom) in GFP-positive SU-DIPG-IV cells transduced with LentiCRISPRv2-GFP lentiviruses. Cells were transduced with a mixture of lentiviruses encoding two sgRNAs, and the scissors and dotted lines indicate the third nucleotide upstream of the protospacer adjacent motif (PAM) site for both gRNAs. The number of edited clones with insertions/deletions predicted to cause loss-of-function, out of the total number of sequenced clones, is indicated to the right.

(D) representative flow cytometry histograms showing GFP fluorescence for cells transduced with lentiviruses encoding control (left panels) or *ASCL1* and *SOX11*-targeting sgRNAs (right panels) at day 3 (red trace) and day 17 (blue trace) post-infection. To adjust for different cell counts, data were normalized to the highest point on the histogram for in each sample (normalized to mode).

(E) Relative percentage of GFP-positive cells over time in SU-DIPG-VI cells transduced with the lentiviruses encoding the indicated sgRNAs.

In all panels, *:$p<0.05$; **:$p<0.01$, assessed by *t*-test (B), or linear regression and slope comparisons (E).

Table S2, related to Figure 5. Expression of *ASCL1* and *SOX11* in normal brain tissue and DIPG tumor samples

Gene expression (FPKM)					
Normal brain		*ACVR1*-WT tumor		*ACVR1*-Mut tumor	
ASCL1	*SOX11*	*ASCL1*	*SOX11*	*ASCL1*	*SOX11*
7.75	4.2	8.62	18.55	31.19	41.06
1.23	2.12	26.62	23.28	5.34	0.99
2.05	1.69	3.15	3.91	17.39	7
1.1	1.34	16.42	11.16	31.12	9.68
2.25	2.71	5.82	7.21	17.85	56.03
2.46	2.49	12.04	39.07		
2.38	1.13	1.18	4.37		
2.24	1.38	8.14	11.12		
1.5	2.67	21.91	19.83		
1.86	1.89	1.56	1.87		
1.36	0.85	25.99	14.89		
1.55	1.78	1.71	3.05		
1.55	1.37	8.31	18.89		
2.09	2.11	11.08	9.56		
2.85	4.04	8.35	5.58		
1.83	1.41	15.89	18.51		
1.58	1.93	24.41	19.66		
		6.65	16.94		
		11.08	12.8		
		16.78	17.08		
		1.32	2.75		
		20.82	23.52		
		4.77	18.1		
		2.66	11.99		
		5.09	6.95		
		12.95	19.51		
		8.25	15.71		
		10.75	40.24		
		2.34	9.56		

Normalized expression of *ASCL1* and *SOX11* in normal brain tissue and *ACVR1*-WT or *ACVR1*-Mut DIPG tumor samples. Each row represents an individual patient. Normal brain: n=17; *ACVR1*-WT: n=29; *ACVR1*-Mut: n=5.

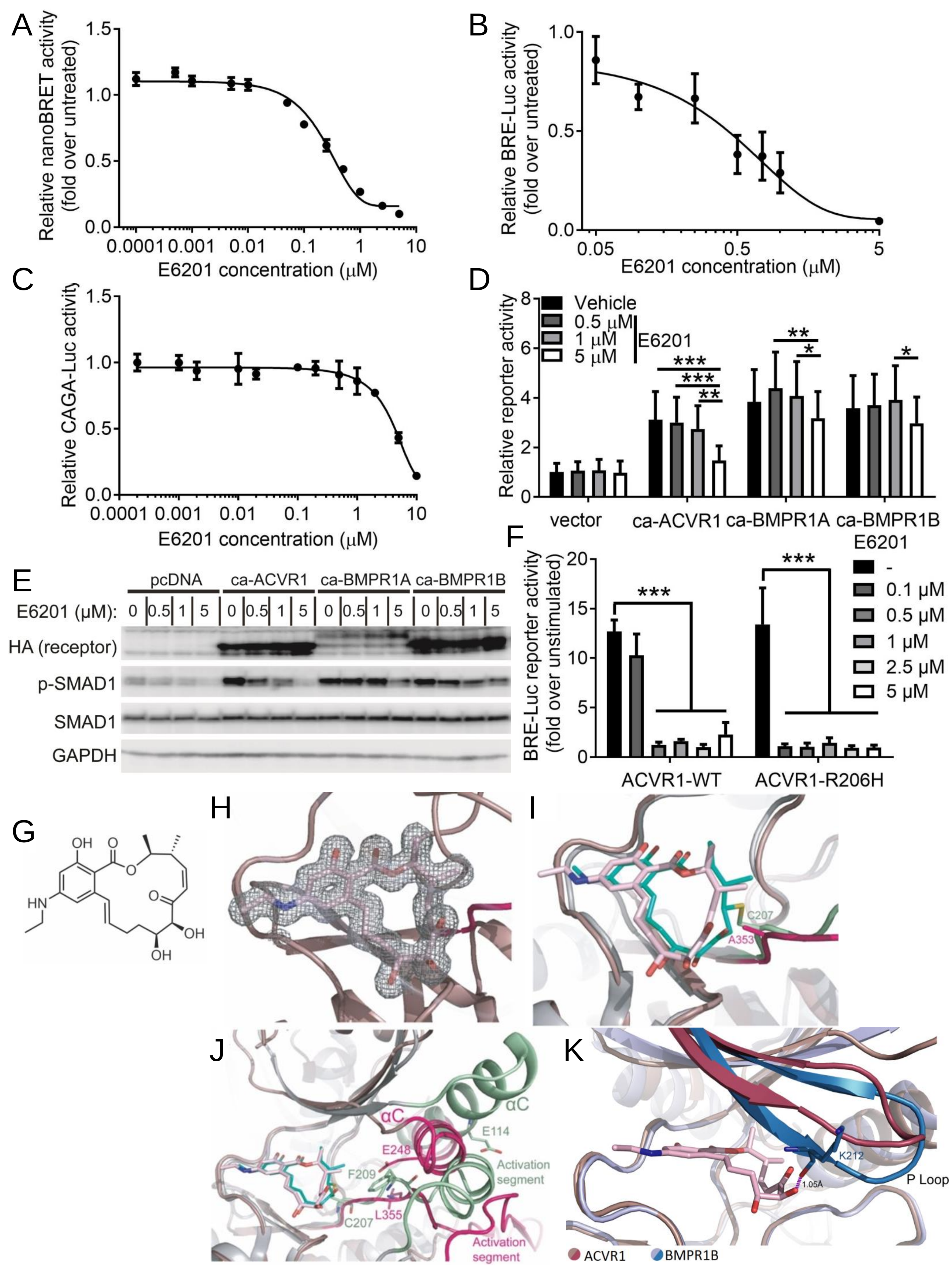

A
Relative nanoBRET activity (fold over untreated)
E6201 concentration (μM)
B
Relative BRE-Luc activity (fold over untreated)
E6201 concentration (μM)
C
Relative CAGA-Luc activity
E6201 concentration (μM)
D
Relative reporter activity
Vehicle
0.5 μM
1 μM
5 μM
E6201
vector
ca-ACVR1
ca-BMPR1A
ca-BMPR1B
E
pcDNA
ca-ACVR1
ca-BMPR1A
ca-BMPR1B
E6201 (μM):
HA (receptor)
p-SMAD1
SMAD1
GAPDH
F
BRE-Luc reporter activity (fold over unstimulated)
E6201
-
0.1 μM
0.5 μM
1 μM
2.5 μM
5 μM
ACVR1-WT
ACVR1-R206H
G
OH
HN
OH
OH
H
I
C207
A353
J
αC
E114
E248
F209
Activation segment
C207
L355
K
K212
1.05Å
P Loop
ACVR1
BMPR1B

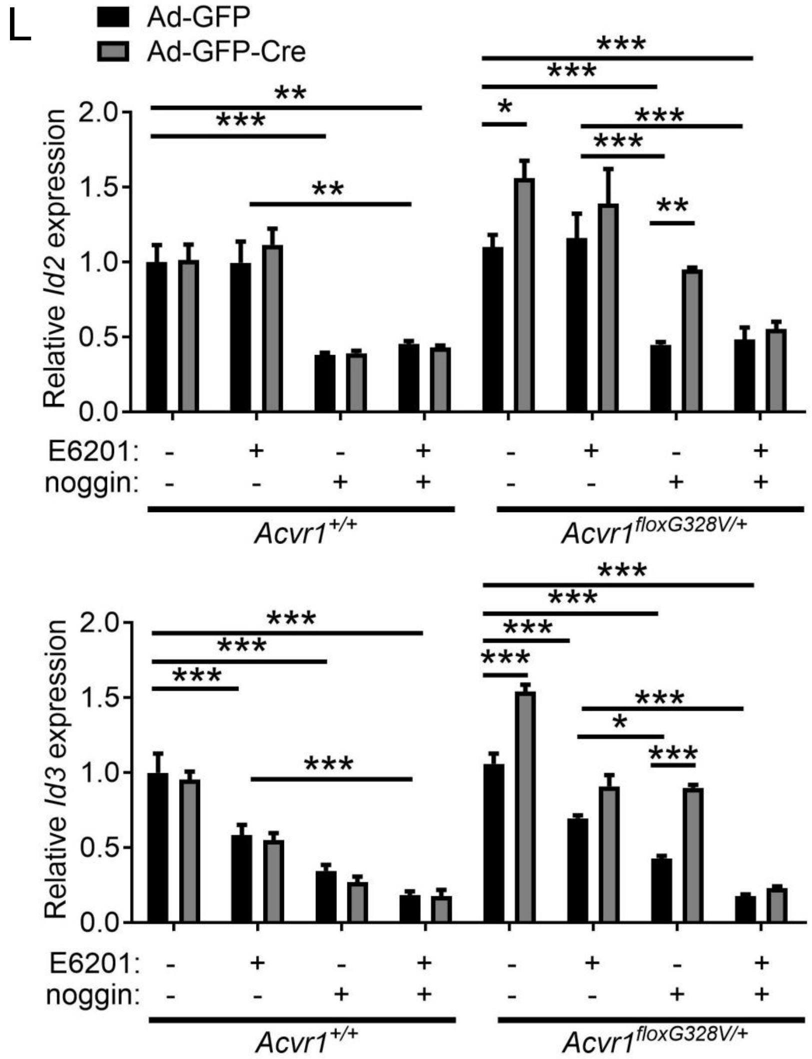

L
Ad-GFP
Ad-GFP-Cre
Relative Id2 expression
2.0
1.5
1.0
0.5
0.0
E6201: - + - + - + - +
noggin: - - + + - - + +
Acvr1+/+
Acvr1floxG328V/+
Relative Id3 expression
2.0
1.5
1.0
0.5
0.0
E6201: - + - + - + - +
noggin: - - + + - - + +
Acvr1+/+
Acvr1floxG328V/+

Figure S6, related to Figure 6. **E6201 inhibits BMP pathway activation induced by wild-type and mutant ACVR1**

(A) NanoBRET target engagement assay demonstrating binding of E6201 to ACVR1. Data shown are pooled from 3 independent experiments.

(B) Luminescent reporter activity in lysates from C2C12 cells transfected with BRE-Luc reporter and treated with 25 ng/mL BMP-6 and the indicated concentrations of E6201 overnight. Data shown are pooled from 3 independent experiments.

(C) Luminescent reporter activity in lysates from HEK-293 cells transfected with CAGA-Luc reporter and treated with 10 ng/ml TGF-β and the indicated concentrations of E6201 for 24 hours (IC_{50}~0.51 μM). Data shown are from triplicate measurements.

(D) Luminescent reporter activity in lysates from HEK-293 cells transfected with the BRE-Luc reporter and constructs encoding the indicated constitutively active (ca-) receptor, or empty vector, and exposed to the indicated concentrations of E6201 overnight. n= 3 independent experiments.

(E) Western blot of whole cell lysates from HEK-293 cell transfected with constructs encoding the indicated HA0-tagged constitutively active (ca-) receptor, or empty vector (pcDNA) probed with HA, p-SMAD1, SMAD1, and GAPDH antibodies. Point mutations for the receptors were: human ca-ACVR1: Q207D; human ca-BMPR1A: Q233D; mouse ca-BMPR1B: Q203D.

(F) Luminescent reporter activity in lysates from C2C12 cells transfected with the BRE-Luc reporter and constructs encoding wild-type (WT) or R206H mutant ACVR1, and exposed to 50 ng/mL BMP6 and the indicated concentrations of E6201 overnight. Data are shown as fold over luminescence activity in untreated cells transfected with wild-type (WT) or R206H mutant ACVR1. n= 3 experiments.

(G) Chemical structure of E6201.

(H) 2Fo-Fc electron density map for E6201 in the ACVR1 complex contoured at 1 σ.

(I) Superposition of the E6201 complexes of ACVR1 (brown ACVR1 ribbon, pink E6201) and MEK1 (grey MEK1 ribbon, green E6201; PDB 5HZE). MEK1 shows covalent binding to E6201 through Cys207, which is replaced by Ala353 in ACVR1.

(J) MEK1 complex shows an αC out, DFG-out conformation in contrast to the αC in, DFG-in conformation of ACVR1. Parts of strands β1 and β2 are omitted for clarity.

(K) Superposition of the ACVR1-E6201 structure (red) with that of the BMPR1B-LDN-193189 inhibitor complex (blue, PDB 3MDY). ACVR1 and BMPR1B adopt distinct inactive conformations of the kinase domain. The BMPR1B structure suggests it favours a more collapsed conformation of the P-loop (β1-β2) that would clash with E6201 (purple dashed line). While no structure exists for the kinase domain of BMPR1A, it is likely to resemble BMPR1B with which it shares 85% sequence identity compared to just 64% for ACVR1.

(L) Expression of *Id2* (top), and *Id3* (bottom), normalized to the expression of *Rpl19*, measured by qPCR on cDNA prepared from primary brainstem glial cells derived from *Acvr1*$^{+/+}$ or *Acvr1flox*$^{G328V/+}$ pups, transduced with Ad-GFP (black bars) or Ad-GFP-Cre (grey bars), and treated where indicated with 100 ng/mL Noggin and/or 1 µM E6201. n=3 experiments from 3 independent litters.

In all panels, *:p<0.05; **:p<0.01; ***:p<0.001, assessed by repeated-measures ANOVA with Tukey multiple comparisons tests.

Table S3, related to Figure 6. X-ray crystallography data collection and refinement statistics.

	ACVR1-FKBP12 complex with E6201
Wavelength	0.9763
Resolution range [Å]	41.32 - 1.52 (1.574 - 1.52)
Space group	$P2_12_12_1$
Cell dimensions [Å] α,β,γ	*a* = 44.3, *b* = 108.6, *c* = 114.4 90°, 90°, 90°
Total reflections	2436319 (240741)
Unique reflections	85798 (8479)
Multiplicity	28.4 (28.4)
Completeness (%)	99.95 (99.99)
Mean *I/σI*	33.17 (1.95)
R-merge	0.712 (2.274)
R-meas	0.727 (2.319)
R-pim	0.143 (0.448)
CC1/2	0.841 (0.581)
CC*	0.956 (0.857)
Reflections (for R-free)	4191 (386)
R-work (%)	17.9 (23.1)
R-free (%)	19.4 (24.8)
# Atoms total	3762
macromolecules	3464
Ligands	61
solvent	237
RMSD bonds [Å]	0.006
RMSD angles [°]	1.18
Ramachandran favored (%)	98.11
Ramachandran allowed (%)	1.89
Average B-factor [$Å^2$]	23.00
Macromolecules [$Å^2$]	22.49
Ligands [$Å^2$]	27.90
Solvent [$Å^2$]	29.14
Number of TLS groups	10

Statistics for the highest-resolution shell are shown in parentheses.

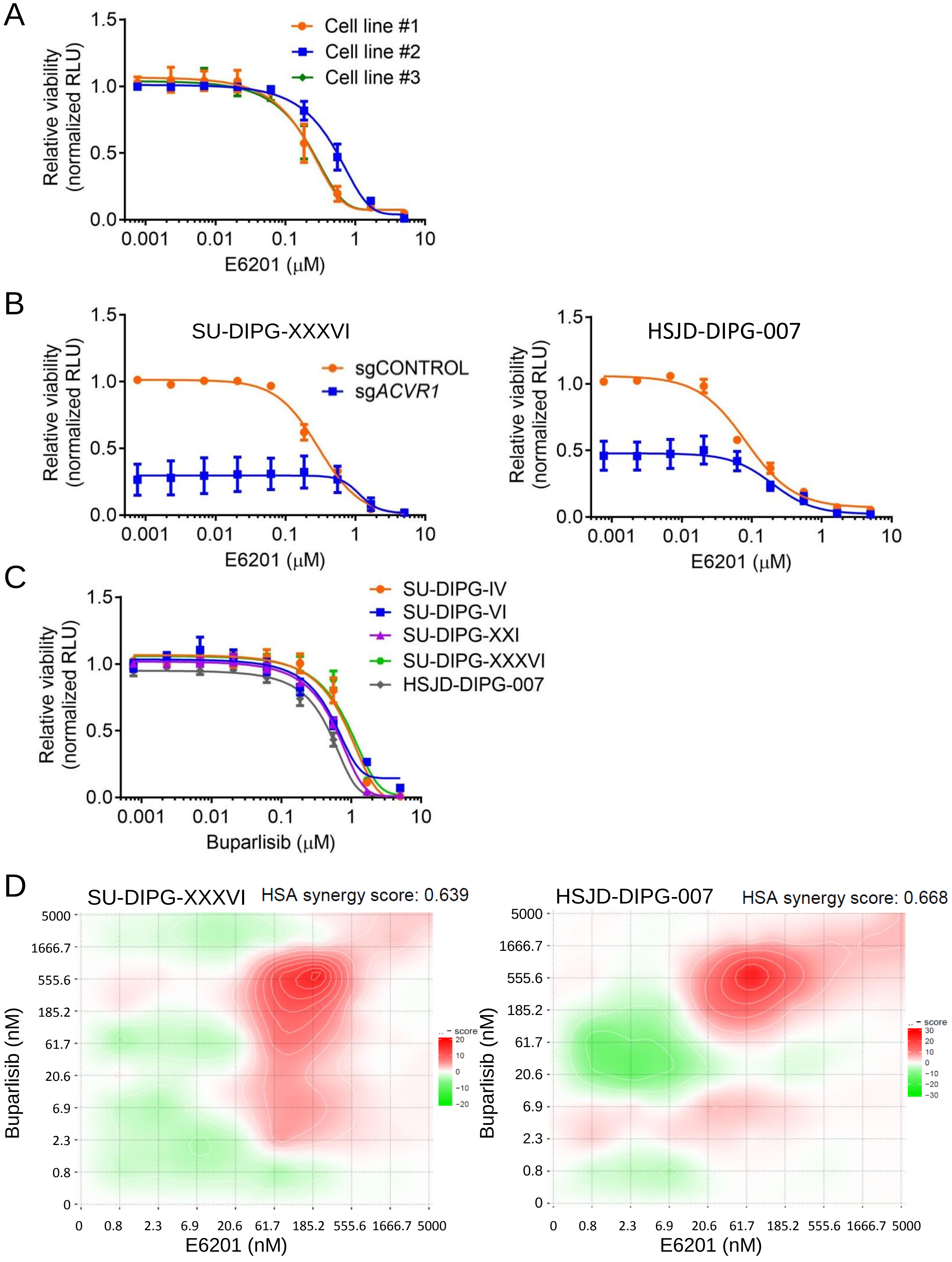
A
Relative viability
(normalized RLU)
E6201 (μM)
Cell line #1
Cell line #2
Cell line #3
B
SU-DIPG-XXXVI
sgCONTROL
sgACVR1
HSJD-DIPG-007
Relative viability
(normalized RLU)
E6201 (μM)
C
SU-DIPG-IV
SU-DIPG-VI
SU-DIPG-XXI
SU-DIPG-XXXVI
HSJD-DIPG-007
Relative viability
(normalized RLU)
Buparlisib (μM)
D
SU-DIPG-XXXVI
HSA synergy score: 0.639
HSJD-DIPG-007
HSA synergy score: 0.668
Buparlisib (nM)
E6201 (nM)
0
0.8
2.3
6.9
20.6
61.7
185.2
555.6
1666.7
5000
score

Figure S7, related to Figure 7. **Characterization of E6201 as an inhibitor of mutant ACVR1**

(A) Relative cell proliferation or viability, measured by the ATPLite ATP-dependent luminescence reaction, in cell lines derived from three *Acvr1*$^{floxG328V/+}$; *Hist1h3b*$^{K27M/+}$; *Pik3ca*$^{floxH1047R/+}$; *Olig2*$^{Cre/+}$ brain tumors, exposed to increasing concentrations of E6201. n=3 experiments for each cell line.

(B) Relative cell proliferation or viability, measured by the ATPLite ATP-dependent luminescence reaction, in SU-DIPG-XXXVI (left) or HSJD-DIPG-007 (right) cells transduced with LentiCRISPRv2-GFP lentiviruses encoding a control (orange line) or *ACVR1*-targeting sgRNAs (blue line), and exposed to increasing concentrations of E6201. Data were normalized to the vehicle-treated condition for *sgCONTROL*. n= 3 experiments for each cell line.

(C) Relative cell proliferation or viability, measured by the ATPLite ATP-dependent luminescence reaction, in DIPG cell lines exposed to increasing concentrations of Buparlisib. n=3 experiments for each cell line.

(D) Visualization of Highest Single Agent (HAS) synergy scores in SU-DIPG-XXXVI (left) or HSJD-DIPG-007 (right) cells exposed to various concentrations of E6201 and/or Buparlisib, based on measurements of ATPLite ATP-dependent luminescence reactions. Score calculations and graphical representation were generated using the SynergyFinder software. Areas of potential synergy are indicated in red; areas of potential antagonism are indicated in green.
