# Supplementary material for: Mutant ACVR1 Arrests Glial Cell Differentiation to Drive Tumorigenesis in Pediatric Gliomas
Source: Cancer Cell. 2020 Mar 16;37(3):308–323.e12. doi: 10.1016/j.ccell.2020.02.002 (PMC7105820; doi:10.1016/j.ccell.2020.02.002)
Supplement: Document S2. Article plus Supplemental Information [file mmc5.pdf]

Article

Cancer Cell

Mutant *ACVR1* Arrests Glial Cell Differentiation to Drive Tumorigenesis in Pediatric Gliomas

Graphical Abstract

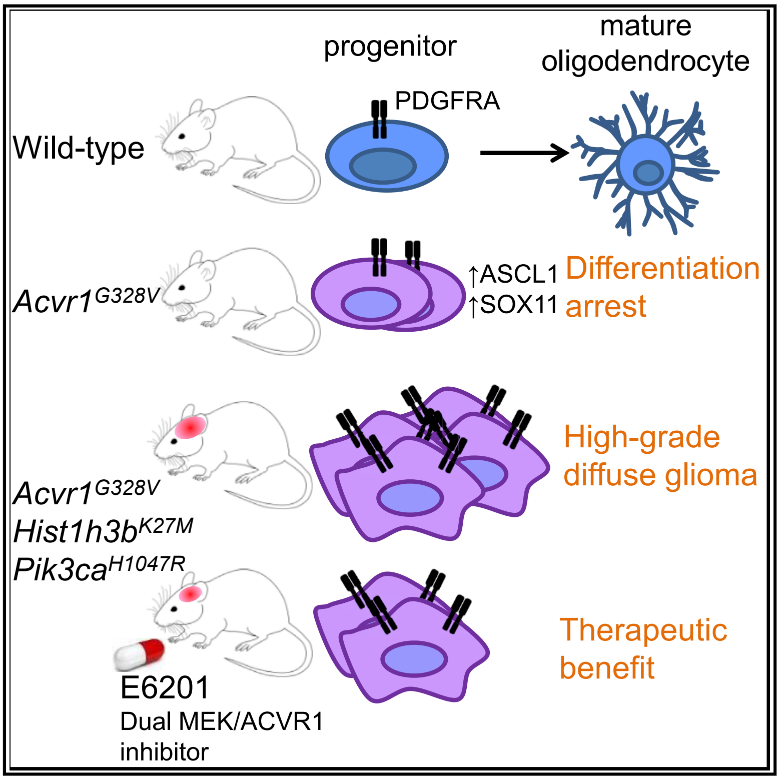

Highlights

- Mouse model of the pediatric high-grade diffuse glioma-driving *Acvr1*^{G328V} mutation
- *Acvr1*^{G328V} causes oligodendroglial lineage differentiation arrest
- Combining *Acvr1*^{G328V}, *Hist1h3b*^{K27M}, and *Pik3ca*^{H1047R} causes high-grade diffuse gliomas
- E6201 is a dual inhibitor of MEK and ACVR1, and shows anti-tumor activity

Authors

Jerome Fortin, Ruxiao Tian, Ida Zarrabi, ..., Peter ten Dijke, Cynthia Hawkins, Tak W. Mak

Correspondence

jerome.fortin@uhnresearch.ca (J.F.), tmak@uhnresearch.ca (T.W.M.)

In Brief

Fortin et al. find that *Acvr1*^{G328V} upregulates transcription factors to block oligodendroglial cell differentiation. *Acvr1*^{G328V} cooperates with *Hist1h3b*^{K27M} and *Pik3ca*^{H1047R} to induce diffuse gliomas in mice. E6201, a covalent MEK1/2 inhibitor, can inhibit ACVR1 and reduce growth of *ACVR1* mutant glioma xenografts.

Fortin et al., 2020, Cancer Cell *37*, 308–323
March 16, 2020 © 2020 Elsevier Inc.
https://doi.org/10.1016/j.ccell.2020.02.002

CellPress

Cancer Cell
Article

Mutant *ACVR1* Arrests Glial Cell Differentiation to Drive Tumorigenesis in Pediatric Gliomas

Jerome Fortin,[1,*] Ruxiao Tian,[1] Ida Zarrabi,[1] Graham Hill,[1] Eleanor Williams,[2] Gonzalo Sanchez-Duffhues,[3] Midory Thorikay,[3] Parameswaran Ramachandran,[1] Robert Siddaway,[4] Jong Fu Wong,[2] Annette Wu,[1] Lorraine N. Apuzzo,[5] Jillian Haight,[1] Annick You-Ten,[1] Bryan E. Snow,[1] Andrew Wakeham,[1] David J. Goldhamer,[5] Daniel Schramek,[6,7] Alex N. Bullock,[2] Peter ten Dijke,[3] Cynthia Hawkins,[4,8,9] and Tak W. Mak[1,10,*]
[1]Princess Margaret Cancer Centre, University Health Network, Toronto, ON M5G 1L7, Canada
[2]Structural Genomics Consortium, University of Oxford, Old Road Campus, Roosevelt Drive, Oxford OX3 7DQ, UK
[3]Department of Cell and Chemical Biology and Oncode Institute, Leiden University Medical Center, P.O. Box 9600 RC, Leiden, the Netherlands
[4]The Arthur and Sonia Labatt Brain Tumour Research Centre, The Hospital for Sick Children, 555 University Avenue, Toronto, ON M5G1X8, Canada
[5]Department of Molecular and Cell Biology, University of Connecticut, Storrs, CT 06268, USA
[6]Center for Systems Biology, Lunenfeld-Tanenbaum Research Institute, Mount Sinai Hospital, Toronto, ON M5G 1X5, Canada
[7]Department of Molecular Genetics, University of Toronto, Toronto, ON M5S 1A8, Canada
[8]Division of Pathology, The Hospital for Sick Children, Toronto, ON M5G 1X8, Canada
[9]Department of Laboratory Medicine and Pathobiology, University of Toronto, Toronto, ON M5S 1A8, Canada
[10]Lead Contact
*Correspondence: jerome.fortin@uhnresearch.ca (J.F.), tmak@uhnresearch.ca (T.W.M.)
https://doi.org/10.1016/j.ccell.2020.02.002

SUMMARY

Diffuse intrinsic pontine gliomas (DIPGs) are aggressive pediatric brain tumors for which there is currently no effective treatment. Some of these tumors combine gain-of-function mutations in *ACVR1*, *PIK3CA*, and histone H3-encoding genes. The oncogenic mechanisms of action of *ACVR1* mutations are currently unknown. Using mouse models, we demonstrate that *Acvr1*^{G328V} arrests the differentiation of oligodendroglial lineage cells, and cooperates with *Hist1h3b*^{K27M} and *Pik3ca*^{H1047R} to generate high-grade diffuse gliomas. Mechanistically, *Acvr1*^{G328V} upregulates transcription factors which control differentiation and DIPG cell fitness. Furthermore, we characterize E6201 as a dual inhibitor of ACVR1 and MEK1/2, and demonstrate its efficacy toward tumor cells *in vivo*. Collectively, our results describe an oncogenic mechanism of action for *ACVR1* mutations, and suggest therapeutic strategies for DIPGs.

INTRODUCTION

Among pediatric brain tumors, diffuse midline gliomas, which include diffuse intrinsic pontine gliomas (DIPGs), carry a particularly poor prognosis (Jones and Baker, 2014; Jones et al., 2017). These tumors cannot be surgically resected, respond only transiently to radiation, and do not reliably respond to conventional chemotherapy or any targeted therapy tested to date (Jones et al., 2017). The recent identification of recurrent genetic lesions in DIPGs provides an opportunity to dissect how these tumors develop, progress, and might be treated (Mackay et al., 2017). Around 85% of DIPGs carry missense mutations in a histone H3-encoding gene, most frequently *H3F3A* or *HIST1H3B*, in which methionine substitutes for lysine at position 27 (H3-K27M) (Mackay et al., 2017; Schwartzentruber et al., 2012; Wu et al., 2012). The tumorigenic effects of K27M mutant histones involve dominant-negative inhibition of H3 K27 trimethylation over large portions of the genome (Bender et al., 2013; Chan et al., 2013; Harutyunyan et al., 2019; Lewis et al., 2013; Mohammad and Helin, 2017; Weinberg et al., 2017).

Significance

There is currently no effective treatment for diffuse intrinsic pontine gliomas (DIPGs), an aggressive type of brain tumor that occurs in children. To better understand how these tumors arise and progress, we analyzed mouse models carrying mutations that recapitulate those that occur in human DIPGs. Our studies uncovered an oncogenic mechanism of action of *Acvr1* mutations, involving an arrest in the maturation of a specific type of glial cells in the brain. Prompted by these findings, we demonstrated the therapeutic potential of a kinase inhibitor that can simultaneously block two oncogenic pathways driving DIPGs.

 © 2020 Elsevier Inc.

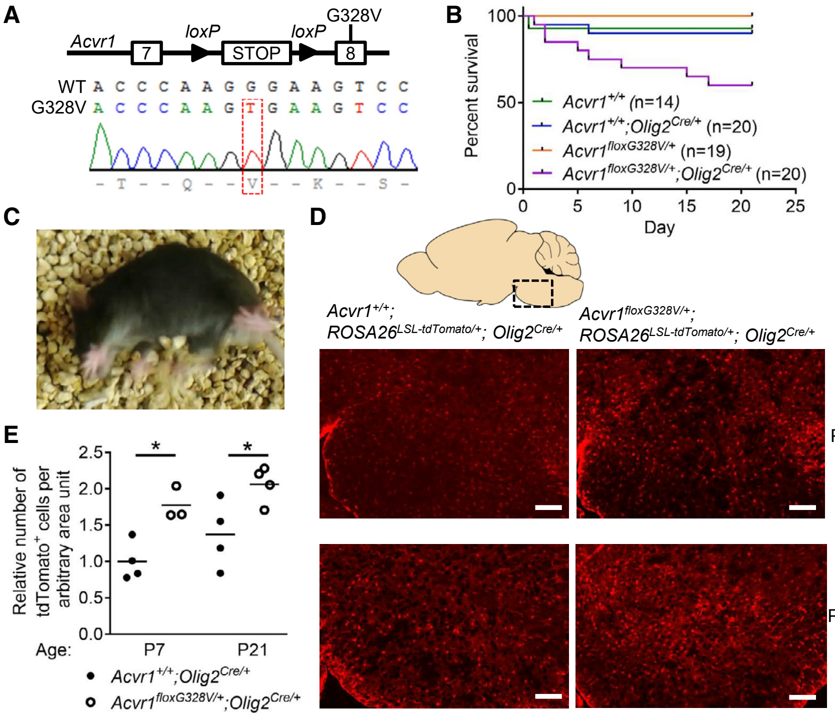

Figure 1. $Acvr1^{G328V}$ Causes Neurological Anomalies and Oligodendroglial Cell Expansion

(A) Schematic of the $Acvr1^{floxG328V}$ allele, and sequencing chromatogram from an $Acvr1^{floxG328V/+}$ (G328V) mouse, showing the G→V substitution.

(B) Survival curves of $Acvr1^{floxG328V/+}$;$Olig2^{Cre/+}$ mice and their littermates.

(C) Snapshot of a video recording of a $Acvr1^{floxG328V/+}$;$Olig2^{Cre/+}$ mouse experiencing spasms and motor anomalies.

(D) Representative images of tdTomato-expressing cells in the brainstem of mice with the indicated genotypes, at postnatal day 7 (P7) and 21 (P21). Scale bars, 100 μm.

(E) Quantification of the data shown in (D). Each dot represents an individual animal (two to four sections measured per mouse). Horizontal bars represent the mean. $^{*}p < 0.05$; assessed by unpaired t test.

See also Figure S1 and Video S1.

DIPG-associated $H3F3A^{K27M}$ and $HIST1H3B^{K27M}$ mutations co-occur with distinct recurrent genetic lesions (Mackay et al., 2017). In particular, approximately 80% of the $HIST1H3B^{K27M}$ tumors contain mutations in *ACVR1* (Buczkowicz et al., 2014; Fontebasso et al., 2014; Taylor et al., 2014a; Wu et al., 2014), which encodes a bone morphogenetic protein (BMP) type I receptor. Around 55% of these tumors also carry mutations that hyperactivate phosphoinositide-3-kinase (PI3K) signaling, especially in *PIK3CA* (Carvalho et al., 2019; Mackay et al., 2017). DIPG-associated *ACVR1* mutations are known or predicted to confer gain of function (Buczkowicz et al., 2014; Fontebasso et al., 2014; Taylor et al., 2014a; Wu et al., 2014) by mechanisms that may include neomorphic ligand responsiveness (Hatsell et al., 2015; Hino et al., 2015) or ligand-independent activation (Mucha et al., 2018). However, the mechanisms by which *ACVR1* mutations exert their oncogenic effects are unknown, and their delineation is crucial for the design of therapeutic strategies for *ACVR1*-mutant tumors.

Analyses of tumor evolution in DIPG patients have indicated that *H3F3A*, *HIST1H3B*, and *ACVR1* mutations occur very early during tumorigenesis, and are positively selected during tumor progression (Hoffman et al., 2016; Nikbakht et al., 2016; Vinci et al., 2018). Additional lesions, such as *PIK3CA* mutations, arise later (Nikbakht et al., 2016; Vinci et al., 2018). Because of their broad effects on epigenetics, H3-K27M mutations have been proposed to reprogram the fate of tumor-initiating glial cells to a more primitive state, or to arrest the differentiation of these cells (Funato et al., 2014; Weinberg et al., 2017). Indeed, differentiation arrest is a hallmark event in the oncogenesis of many types of brain tumors (Lan et al., 2017; Tirosh et al., 2016). Recent single-cell transcriptomic studies lend credence to the importance of this process in DIPGs, suggesting that these tumors are fueled by cells that are similar to oligodendrocyte precursors cells (OPCs) (Filbin et al., 2018). However, the underlying mechanisms have yet to be defined. Here, by generating and analyzing a conditional knockin mouse model of the DIPG-causing $ACVR1^{G328V}$ mutation, we aimed to uncover how mutant ACVR1 drives tumorigenesis, and could be therapeutically targeted.

RESULTS

Expression of $Acvr1^{G328V}$ in Murine Oligodendroglial Cells Causes Neurological Anomalies

To model the DIPG-causing $Acvr1^{G328V}$ mutation in mice, we engineered a conditional knockin allele, $Acvr1^{floxG328V}$ (Figure 1A). We inserted a *loxP*-flanked transcriptional stop cassette in intron 7, upstream of a mutant exon 8 encoding the G328V substitution. Mice expressing the recombined $Acvr1^{G328V}$ allele in the whole body died before or around birth, showing obvious developmental anomalies (Figures S1A and S1B). To evaluate the effect of targeting the $Acvr1^{G328V}$ mutation to a broad population of neuroglial progenitors, we crossed the $Acvr1^{floxG328V}$ allele with the *Nestin-Cre* driver. However, the resulting animals showed no obvious abnormal phenotype. OLIG2-expressing cells in the ventral brainstem of juvenile mice and humans, most of which do not express Nestin, have been identified as candidate tumor-initiating cells in DIPG (Lindquist et al., 2016; Monje et al., 2011). Therefore, we used $Olig2^{Cre}$ to target the $Acvr1^{G328V}$ mutation to OPCs. $Acvr1^{floxG328V/+}$;$Olig2^{Cre/+}$ mice were born at the expected Mendelian ratio, but some of them failed to gain normal body weight and died before weaning (Figures 1B and S1C). By the third postnatal week, most surviving $Acvr1^{floxG328V/+}$;$Olig2^{Cre/+}$ animals developed overt neurological anomalies, often showing pronounced spasms when disrupted during rest, and moderate ataxia (Figure 1C; Video S1).

To verify whether the neurological defects observed in $Acvr1^{floxG328V/+}$;$Olig2^{Cre/+}$ mice were associated with abnormal

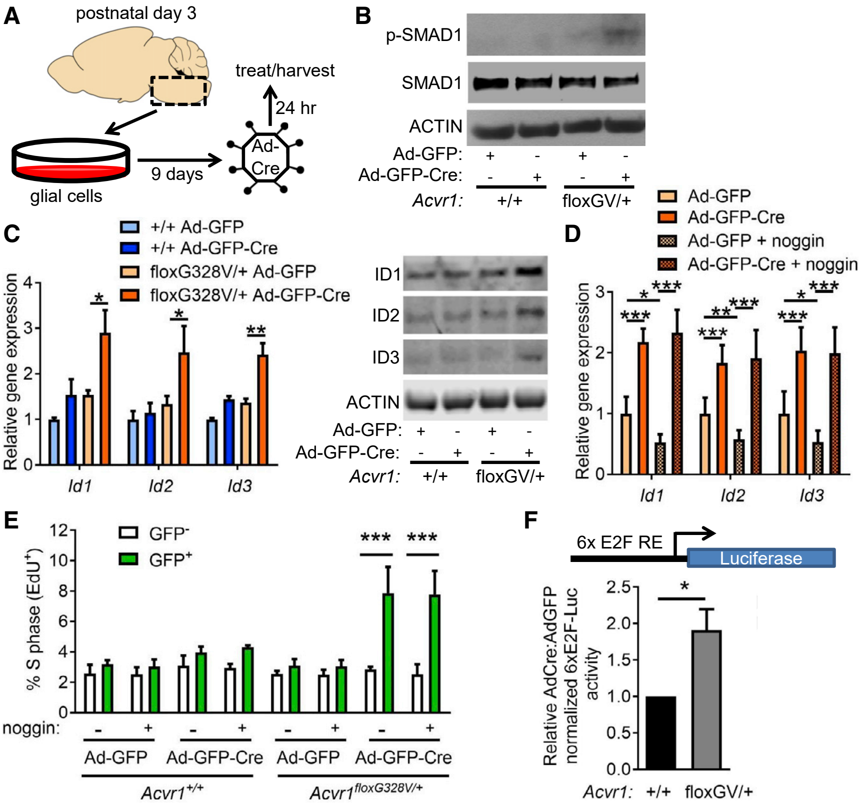

Figure 2. *Acvr1*^{G328V} Hyperactivates BMP Signaling and Stimulates Glial Cell Proliferation

(A) Schematic depicting experiments in primary glial cells.

(B) Western blot of lysates from *Acvr1*$^{+/+}$ or *Acvr1*$^{floxG328V/+}$ (floxGV) glial cell, transduced with Ad-GFP or Ad-GFP-Cre, probed with the indicated antibodies.

(C) mRNA expression, measured by qPCR (left) and protein levels (right), assessed by western blot of Id1, Id2, and Id3, in *Acvr1*$^{+/+}$ or *Acvr1*$^{floxG328V/+}$ (floxGV) brainstem glial cells transduced with Ad-GFP or Ad-GFP-Cre. For qPCR, n = 3 experiments.

(D) Expression of *Id1*, *Id2*, and *Id3*, measured by qPCR in *Acvr1*$^{+/+}$ or *Acvr1*$^{floxG328V/+}$ brainstem glial cells transduced with Ad-GFP or Ad-GFP-Cre, and treated or not with 100 ng/mL noggin. n = 4 experiments.

(E) Percentage of 5-ethynyl-2′-deoxyuridine (EdU)-positive cells in GFP-negative and GFP-positive *Acvr1*$^{+/+}$ or *Acvr1*$^{floxG328V/+}$ glial cells transduced with Ad-GFP or Ad-GFP-Cre, and incubated with 10 μM EdU for 2 h. n = 3 experiments.

(F) Normalized E2F-Luc reporter activity in *Acvr1*$^{+/+}$ or *Acvr1*$^{floxG328V/+}$ glial cells, transduced Ad-GFP-Cre, relative to reporter activity in cells transduced with Ad-GFP. n = 4 experiments.

In all panels, mean + SEM is shown. *p < 0.05, **p < 0.01, ***p < 0.001; assessed by repeated-measures ANOVA (C–E) with Sidak (C and D), or Dunnett (E) multiple comparisons test, or paired t test (F). See also Figure S2.

function or impaired survival of oligodendroglial cells, we first examined the fate of cells carrying the *Acvr1*^{G328V} mutation by using the *ROSA26*$^{LSL\text{-}tdTomato}$ reporter allele. Quantification of tdTomato^{+} cells in the ventral brainstem at postnatal days 7 (P7) and 21 revealed an approximately 2-fold increase in the number of lineage-traced cells in *Acvr1*$^{floxG328V/+}$;*Olig2*$^{Cre/+}$;*ROSA26*$^{LSL\text{-}tdTomato}$ animals compared with their littermate controls (Figures 1D and 1E). These results suggest that the *Acvr1*^{G328V} mutation favors oligodendroglial cell expansion, and that the neurological symptoms observed are likely due to the dysfunction of these cells.

Acvr1^{G328V} Induces Hyperactive BMP Signaling and Glial Cell Proliferation

To investigate the molecular mechanisms underlying the oligodendroglial lineage cell expansion induced by *Acvr1*^{G328V}, we generated primary glial cell cultures from neonatal *Acvr1*$^{+/+}$ and *Acvr1*$^{floxG328V/+}$ mouse brainstems (Figure 2A). Cells were transduced with adenoviruses encoding GFP (Ad-GFP) or GFP plus Cre (Ad-GFP-Cre). In *Acvr1*$^{floxG328V/+}$ cells, Ad-GFP-Cre triggered recombination of the conditional allele and stimulated the phosphorylation of the canonical BMP signaling effector, SMAD1, but not of SMAD2 (Figures 2B, S2A, and S2B). Ad-GFP-Cre-transduced *Acvr1*$^{floxG328V/+}$ cells expressed higher mRNA and protein levels of the BMP target genes, *Id1*, *Id2*, and *Id3* (Figure 2C). Addition of the BMP ligand antagonist noggin decreased basal, but not *Acvr1*^{G328V}-stimulated *Id1/2/3* expression (Figure 2D). Some activating *ACVR1* mutations have been shown to confer activin responsiveness to the receptor (Hatsell et al., 2015; Hino et al., 2015). However, the activin antagonist follistatin, alone or combined with noggin, did not prevent *Id1/2/3* gene induction by *Acvr1*^{G328V} in glial cells (Figure S2C). These data suggest that the gain-of-function effects of the *Acvr1*^{G328V} mutation can be mediated independently of certain extracellular BMP ligands and activins. *Acvr1*^{G328V} stimulated moderate cell proliferation, as judged by the incorporation of 5-ethynyl-2′-deoxyuridine (Figure 2E). Accordingly, glial cells from *Acvr1*$^{floxG328V/+}$;*Olig2*$^{Cre/+}$ mice exhibited a growth advantage compared with those from their *Acvr1*$^{+/+}$;*Olig2*$^{Cre/+}$ littermates (Figure S2D).

The ID proteins can stimulate cell proliferation by inhibiting the expression of negative cell-cycle regulators or by interfering with the ability of the retinoblastoma-associated protein (Rb) to suppress the activity of E2F transcription factors (Lasorella et al., 2014). Therefore, we measured the expression of E2F-dependent genes that drive the G1-to-S cell-cycle transition. *Ccna2* and *Cdc25a* were upregulated upon transduction of primary glial cells from *Acvr1*$^{floxG328V/+}$, but not *Acvr1*$^{+/+}$ pups, with Ad-GFP-Cre (Figure S2E). A similar trend was observed for *Ccne1* (Figure S2E). To verify whether the upregulation of these genes reflected enhanced E2F-dependent transcriptional activity, we transfected primary glial cells with an E2F-dependent luciferase reporter construct. Compared with *Acvr1*$^{+/+}$ controls, *Acvr1*$^{floxG328V/+}$ cells transduced with Ad-GFP-Cre showed greater reporter activity (Figure 2F). Levels of phosphorylated Rb, which were lower than in cells cultured in the presence of serum, remained unchanged across all conditions in these experiments (Figure S2F). Overall, these results suggest that *Acvr1*^{G328V}-dependent induction of

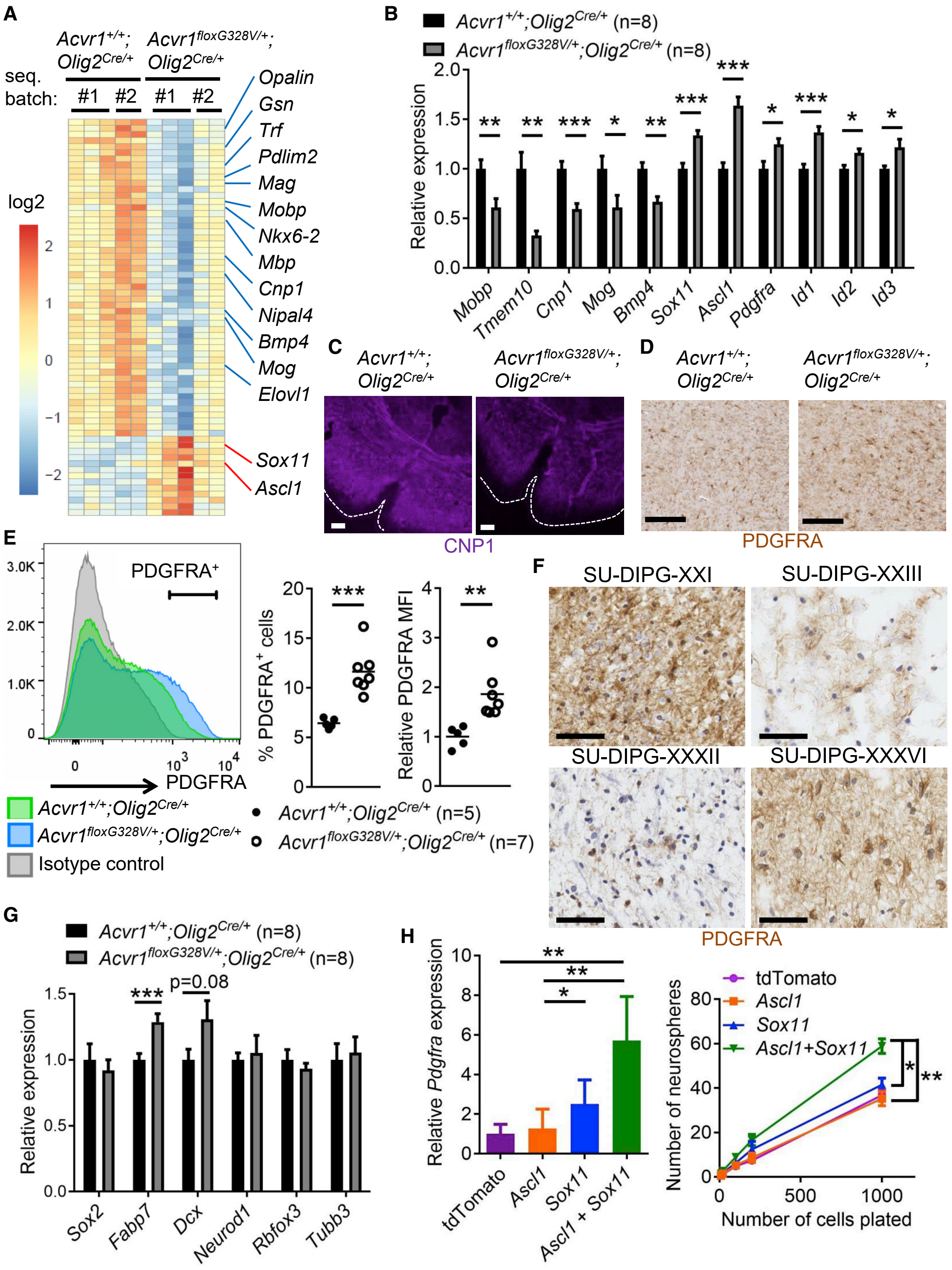

Figure 3. ***Acvr1*** ^{G328V} **Causes Differentiation Arrest of Oligodendroglial Lineage Cells**

(A) Heatmap depicting the relative expression of the top differentially expressed genes in the brainstem of postnatal day 7 (P7) pups.

(B) Expression of selected genes, measured by qPCR, in the brainstems from P7 pups.

(C) Immunofluorescence images showing CNP1 expression in the brainstem of mice at P21. Scale bars, 100 μm.

(D) Representative PDGFRA immunohistochemistry images in brainstem sections from mice at P14. Scale bars, 100 μm.

(legend continued on next page)

Id1/2/3 expression drives cell-cycle proliferation by enhancing the activity of E2F transcription factors (Figure S2G).

Acvr1^{G328V} Blocks Oligodendrocyte Differentiation and Upregulates PDGFRA

To more comprehensively delineate the molecular changes induced by the *Acvr1^{G328V}* mutation in the oligodendroglial lineage, we used RNA sequencing (RNA-seq) to profile the transcriptome of whole brainstems from postnatal day 7 *Acvr1$^{floxG328V/+}$;Olig2$^{Cre/+}$* and *Acvr1$^{+/+}$;Olig2$^{Cre/+}$* littermates. A total of 247 genes were differentially expressed between the genotypes, with a corrected p value < 0.05 (Table S1). Of these genes, 125 were upregulated, while 122 were downregulated. Several of the most downregulated genes in *Acvr1$^{floxG328V/+}$;Olig2$^{Cre/+}$* pups were markers of oligodendrocyte maturation, such as *Cnp1*, *Mobp*, *Mog*, and *Opalin* (*Tmem10*) (Figure 3A). We confirmed these results by qPCR for several genes (Figure 3B), and by immunostaining for CNP1 (Figure 3C). In the brainstems of *Acvr1$^{floxG328V/+}$;Nestin-Cre* pups, expression of these genes was either normal or mildly altered, while *Id1* and *Id3* were upregulated, likely reflecting *Acvr1^{G328V}* activation in non-oligodendrocyte lineage cells (Figure S3A). Gene set enrichment analysis confirmed downregulation of the oligodendrocyte differentiation program in *Acvr1$^{floxG328V/+}$;Olig2$^{Cre/+}$* brainstems, as well as upregulation of BMP signaling (Figures S3B and S3C).

Notably, expression of *Pdgfra*, an OPC marker, was upregulated in *Acvr1$^{floxG328V/+}$;Olig2$^{Cre/+}$* brainstems (Figure 3B; Table S1). Although the *PDGFRA* gene is amplified or mutated in some pediatric high-grade gliomas and DIPGs, these alterations are very rarely seen in *ACVR1*-mutant tumors (Mackay et al., 2017). Thus, the transcriptional upregulation of *Pdgfra* induced by *Acvr1^{G328}* may serve as an alternative mechanism to gene amplification for enhancing PDGF signaling. Immunohistochemistry indicated a higher density of PDGFRA^{+} cells in *Acvr1$^{floxG328V/+}$;Olig2$^{Cre/+}$* brainstems (Figures 3D and S3D). To confirm this phenotype, we used flow cytometry to measure PDGFRA protein on the surface of brainstem cells from *Acvr1$^{floxG328V/+}$;Olig2$^{Cre/+}$* and *Acvr1$^{+/+}$;Olig2$^{Cre/+}$* P7 littermates. The proportion of PDGFRA^{+} cells, as well as the relative intensity of the PDGFRA signal, were both increased in *Acvr1$^{floxG328V/+}$;Olig2$^{Cre/+}$* pups (Figure 3E). To assess the relevance of these observations for human tumors, we examined PDGFRA expression in histological sections from a panel of four *ACVR1* mutant DIPGs. In all cases, we observed prominent PDGFRA immunoreactivity in a proportion of cells, ranging from widespread (SU-DIPG-XXI, SU-DIPG-XXXVI) to more restricted (SU-DIPG-XXIII, SU-DIPG-XXXII) (Figure 3F). Such variability is expected from the heterogeneous nature of the tumors and tissue samples, and is consistent with the presence of a malignant cell population with oligodendroglial characteristics in most DIPGs (Filbin et al., 2018).

To assess whether gene expression changes induced by *Acvr1^{G328V}* extend to other *Acvr1* mutations, we analyzed *Acvr1$^{tnR206H/+}$;Pdgfra-Cre* mice. In this model, the DIPG-causing *Acvr1^{R206H}* mutation is targeted to *Pdgfra*-expressing cells, which includes oligodendrocyte progenitors throughout the brain (Carter et al., 2012; Lees-Shepard et al., 2018). We confirmed *Pdgfra-Cre* activity in the brainstem (Figure S3E). Genes altered in the brainstems of *Acvr1$^{floxG328V/+}$;Olig2$^{Cre/+}$* mice were similarly affected in *Acvr1$^{tnR206H/+}$;Pdgfra-Cre* pups at P7, albeit more modestly (Figure S3F). Therefore, distinct activating *Acvr1* mutations have overlapping cellular effects.

To characterize the mechanisms whereby the *Acvr1^{G328V}* mutation impairs differentiation, we focused on transcription factors that control oligodendrocyte maturation. *Ascl1* and *Sox11* were among the top upregulated genes encoding transcription factors in *Acvr1$^{floxG328V/+}$;Olig2$^{Cre/+}$* P7 brainstems (Figures 3A and 3B; Table S1). Expression of both genes can be induced by BMP signaling in other contexts (Gadi et al., 2013; Shah et al., 1996). *Ascl1* was also upregulated in the brainstems of *Acvr1$^{tnR206H/+}$;Pdgfra-Cre* and *Acvr1$^{floxG328V/+}$;Nestin-Cre* mice, the latter being likely due to activation of *Acvr1^{G328V}* in the neuronal lineage (Figures S3A and S3F). ASCL1 and SOX11 have been implicated in the control of oligodendroglial progenitor formation and maturation (Basak et al., 2018; Cahoy et al., 2008; Dugas et al., 2008; Nakatani et al., 2013; Swiss et al., 2011). Ectopic expression of ASCL1 in adult glioblastoma cells induces features of neuronal maturation and inhibition of glial cell differentiation (Park et al., 2017). Accordingly, RNA-seq analyses suggested that the neuroblast marker *Dcx* was upregulated in *Acvr1$^{floxG328V/+}$;Olig2$^{Cre/+}$* P7 brainstems, although only a moderate trend was detected by qPCR (Figure 3G; Table S1). Whereas expression of the neural stem cell marker *Sox2* was normal in *Acvr1$^{floxG328V/+}$;Olig2$^{Cre/+}$* pups, the neuroglial progenitor marker *Fabp7* was upregulated (Figure 3G). Expression levels of more mature neuronal markers were comparable between the genotypes (Figure 3G). In *Acvr1$^{+/+}$* neural stem cells, cultured in the presence of PDGF ligands, lentivirus-mediated ectopic expression of *Ascl1* and *Sox11*, but not either alone, increased the expression of *Pdgfra*, and enhanced neurosphere-forming ability (Figure 3H). Together, these results suggest that the *Acvr1^{G328V}* mutation blocks the differentiation of oligodendroglial lineage cells, and upregulates neuroglial progenitor markers.

An Endogenous *Hist1h3b^{K27M}* Mutation Cooperates with *Acvr1^{G328V}* to Induce the Expression of BMP Target Genes

The absence of tumors in *Acvr1$^{floxG328V/+}$;Olig2$^{Cre/+}$* mice suggested that additional genetic lesions are needed for gliomagenesis. Most *ACVR1*-mutant DIPGs carry *HIST1H3B^{K27M}* (Mackay

(E) Representative flow cytometry histograms, and quantification of the percentage of positive cells and mean fluorescence intensity, of PDGFRA antibody-stained brainstem cells from P7 pups. Each dot represents an individual animal; horizontal bars represent the mean.

(F) Representative immunohistochemistry images showing PDGFRA protein expression in four *ACVR1* mutant DIPG samples. Scale bars, 50 μm.

(G) Expression of selected genes, measured by qPCR, in the brainstems of P7 pups.

(H) Expression of *Pdgfra* (left), and number of neurospheres generated, plotted as a function of the number of cells plated (right) in *Acvr1$^{+/+}$* neural stem cells transduced with lentiviruses encoding tdTomato, *Ascl1*, *Sox11*, or both *Ascl1* and *Sox11*. n = 5 (left) and 3 (right) experiments.

In all panels, mean + SEM is shown. *p < 0.05, **p < 0.01, ***p < 0.001; assessed by unpaired t test (B, E, and G), repeated-measures ANOVA with Tukey multiple comparisons tests (H). See also Figure S3 and Table S1.

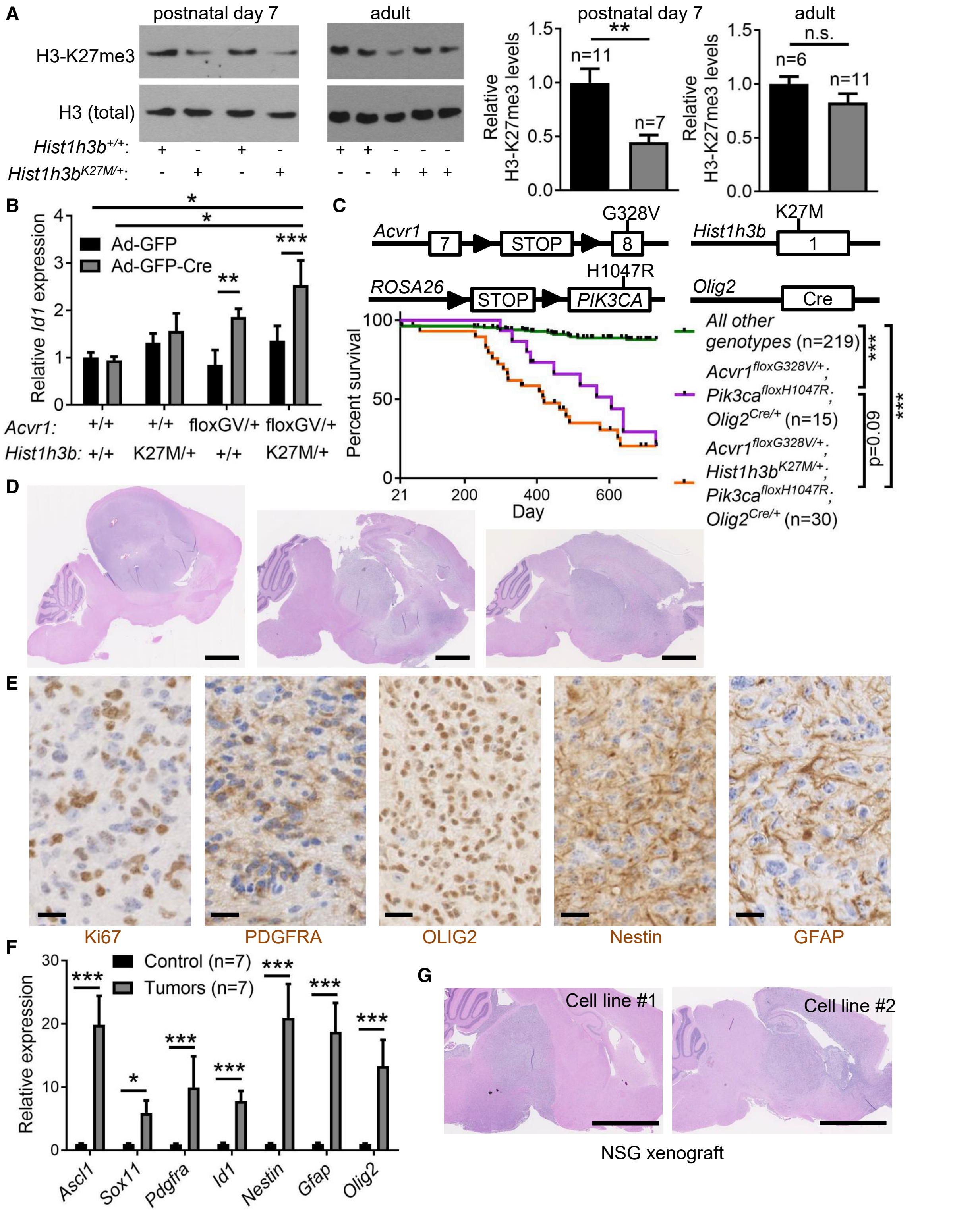

Figure 4. ***Acvr1*** ^{G328V} **and** ***Pik3cA*** ^{H1047R} **Cooperate to Induce High-Grade Diffuse Gliomas in Mice**

(A) Left: representative western blots of brainstem tissue lysates from postnatal day 7 or adult mice, probed with the indicated antibodies. Right: quantification.

(B) Expression of *Id1* measured by qPCR, in glial cells carrying the indicated combination of alleles ("floxGV": *Acvr1* ^{floxG328V}), transduced with Ad-GFP or Ad-GFP-Cre, and treated with 100 ng/mL noggin. n = 3 experiments.

(C) Schematic of the *Acvr1* ^{floxG328V}, *Hist1h3b* ^{K27M}, *Pik3ca* ^{floxH1047R}, and *Olig2* ^{Cre} knockin alleles, and survival curves of mice carrying the indicated combinations of alleles.

(legend continued on next page)

et al., 2017). To model this mutation, we generated a mouse *Hist1h3b*^{K27M} knockin allele. *Hist1h3b*$^{K27M/+}$ mice were viable and appeared to develop normally. Western blot analyses on postnatal day 7 brainstems revealed a global decrease in the level of H3 trimethylated at lysine 27 (H3-K27me3) in *Hist1h3b*$^{K27M/+}$ mice (Figure 4A), in accordance with the known cellular effects of H3-K27M. However, this decrease was no longer evident in young adult *Hist1h3b*$^{K27M/+}$ animals (Figure 4A), suggesting compensatory mechanisms or time-restricted effects. Addition of the *Hist1h3b*^{K27M} mutation in *Acvr1*$^{floxG328V/+}$;*Olig2*$^{Cre/+}$ mice did not substantially affect their partial early postnatal lethality and did not induce detectable brain tumors (Figure S4A).

Cells in primary brainstem glial cultures from *Hist1h3b*$^{K27M/+}$ pups showed reduced global H3-K27me3 levels (Figure S4B) and proliferated slightly faster than their *Hist1h3b*$^{+/+}$ counterparts (Figure S4C). In these cells, induction of *Id1/2/3* gene expression by Ad-GFP-Cre transduction was highest in the presence of both the *Acvr1*^{floxG328V} and *Hist1h3b*^{K27M} alleles, with *Hist1h3b*^{K27M} having a small effect by itself (Figures 4B and S4D). Accordingly, there was a higher proportion of proliferating cells in primary glial cultures carrying the *Acvr1*^{G328V} and/or the *Hist1h3b*^{K27M} mutations (Figure S4E). Concurrently, expression of *Acvr1*^{G328V} plus *Hist1h3b*^{K27M} additively stimulated E2F-dependent transcriptional activity (Figure S4F). To assess whether this was associated with epigenetic priming induced by H3-K27M, we performed chromatin immunoprecipitation experiments. When compared with the repressed *Hoxd8* promoter, the BMP-responsive elements (BREs) of the *Id1* gene promoter (Korchynskyi and ten Dijke, 2002) showed relatively low H3-k27me3 occupancy in *Hist1h3b*$^{+/+}$ glial cells (Figure S4G). Mutant H3-K27M was detected at the promoter of both genes, whereas SMAD1 was only bound to the BREs of *Id1* (Figure S4G). In *Hist1h3b*$^{K27M/+}$ cells, robust H3-K27me3 occupancy was maintained at the *Hoxd8* promoter, but reduced at the *Id1* promoter (Figure S4G), consistent with previously described effects of H3-K27M on H3-K27me3 deposition at repressed versus active loci (Harutyunyan et al., 2019; Mohammad et al., 2017; Piunti et al., 2017). Together, these results suggest that epigenetic changes driven by endogenous *Hist1h3b*^{K27M} may facilitate BMP target gene induction and cell proliferation stimulated by *Acvr1*^{G328V} (Figure S2G), but is not sufficient to drive tumor formation in our mouse model.

Acvr1^{G328V}, *Hist1h3b*^{K27M}, and *Pik3ca*^{H1047R} Cooperate to Induce High-Grade Diffuse Gliomas

In addition to *ACVR1* and *HIST1H3B*, several DIPGs harbor *PIK3CA* mutations (Carvalho et al., 2019; Mackay et al., 2017). To accurately model this combination, we generated *Acvr1*$^{floxG328V/+}$;*Hist1h3b*$^{K27M/+}$;*Pik3ca*$^{floxH1047R/+}$;*Olig2*$^{Cre/+}$ mice. Young animals carrying this genotype exhibited neurological symptoms that were comparable with those observed in *Acvr1*$^{floxG328V/+}$;*Olig2*$^{Cre/+}$ mice. Most of the *Acvr1*$^{floxG328V/+}$;*Hist1h3b*$^{K27M/+}$;*Pik3ca*$^{floxH1047R/+}$;*Olig2*$^{Cre/+}$ mice succumbed to spontaneous brain tumors, with a median survival of 419 days (Figure 4C). Separating mice according to the various mutation combinations indicated that *Hist1h3b*^{K27M} is not required for the appearance of the tumors, but may accelerate their development and/or increase their incidence (Figure 4C). Importantly, tumors were never seen in *Olig2*$^{Cre/+}$ mice carrying exclusively the *Acvr1*^{floxG328V} or *Pik3ca*^{floxH1047R} alleles, with or without *Hist1h3b*^{K27M}. Histopathological analyses indicated that the tumors were invariably high-grade diffuse gliomas (Figure 4D). The tumors often infiltrated throughout many parts of the brain, particularly in the midbrain and thalamic regions, and more rarely involved the brainstem (Figure S4H). The diffuse gliomas contained abundant mitotic figures and proliferating cells, as indicated by Ki67 immunohistochemistry (Figure 4E). The tumors also expressed PDGFRA and OLIG2 (Figure 4E), consistent with an oligodendroglial origin or phenotype, and contained a substantial population of cells that were positive for the glial/progenitor markers, Nestin and GFAP (Figure 4E). qPCR analyses confirmed upregulation of *Pdgfra*, *Olig2*, *Nestin*, and *Gfap* in gliomas, compared with matched normal brain tissue from control littermates (Figure 4F). The tumors also showed elevated expression of *Ascl1*, *Sox11*, and *Id1*, indicating that they preserve and amplify gene expression changes driven by *Acvr1*^{G328V} (Figure 4F). Cell lines derived from the tumors maintained elevated expression of these genes (Figure S4I), and could generate high-grade diffuse gliomas when transplanted in the brains of NOD SCID gamma (NSG) mice (Figure 4G). Overall, these results indicate that the *Acvr1*^{G328V} and *Pik3ca*^{H1047R} mutations cooperate to induce high-grade diffuse gliomas when targeted to the *Olig2*-expressing lineage.

ASCL1 and SOX11 Regulate Human DIPG Cell Fitness and Tumorigenicity

The above observations raised the possibility that ASCL1 and SOX11 mediate differentiation arrest and tumorigenesis in DIPGs. To evaluate the relevance of these factors in human DIPGs, we measured their expression in samples of normal human brain tissue and DIPG tumors. RNA-seq analyses revealed markedly increased expression of *ASCL1* and *SOX11* in DIPG samples compared with normal brain tissue, irrespective of the driver mutations (Figures 5A and S5A; Table S2). Furthermore, expression of these two genes was strongly positively correlated (Figures 5A and S5A). In *ACVR1* mutant DIPG cell lines, the BMP receptor inhibitor, LDN-193189, variably suppressed *ASCL1* and *SOX11* expression (Figure S5B).

To test the functional roles of ASCL1 and SOX11 in DIPG tumor cells, we used CRISPR/Cas9-mediated gene editing to

(D) Representative H&E-stained brain tissue sections showing diffuse high-grade gliomas in three mice carrying the *Acvr1*^{floxG328V}, *Pik3ca*^{floxH1047R}, and *Olig2*^{Cre} alleles without (left), or with (middle and right) *Hist1h3b*^{K27M}. Scale bars, 2.5 mm.

(E) Representative immunohistochemistry images showing expression of the indicated proteins in tumors. Scale bars, 20 μm.

(F) Expression of selected genes, measured by qPCR, in tumors and matched normal brain tissue from control littermates.

(G) Representative H&E-stained sagittal brain tissue sections of NOD SCID gamma (NSG) mice xenografted in the brainstem (left) or midbrain (right) with cell lines derived from mouse tumors, at humane endpoint. Scale bars, 2.5 mm.

In all panels, mean + SEM is shown.*$p < 0.05$, **$p < 0.01$, ***$p < 0.001$; assessed by unpaired t tests (A and F), repeated-measures ANOVA with Tukey multiple comparisons tests (B), or Gehan-Breslow-Wilcoxon test (C). See also Figure S4.

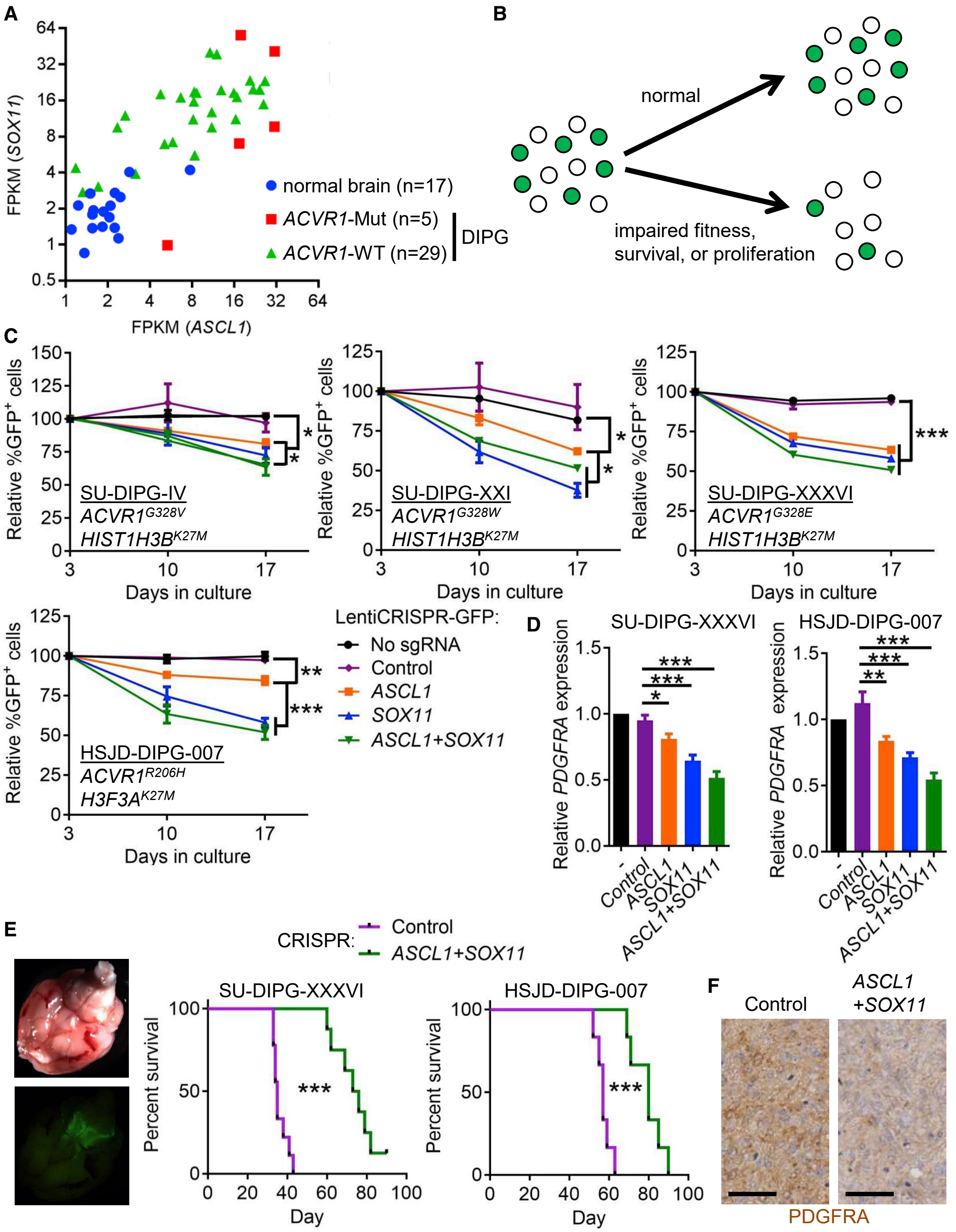

Figure 5. *ASCL1* and *SOX11* Inactivation Impairs DIPG Cell Fitness

(A) *ACSCL1* and *SOX11* expression in *ACVR1*-mutant and *ACVR1*-wild-type DIPG tumors, or in normal brain tissue, calculated as fragments per kilobase million (FPKM).

(B) Schematic depicting experiments measuring the relative fitness of tumor cells transduced with LentiCRISPRv2-GFP.

(C) Relative percentage of GFP-positive cells over time in the indicated cell lines, transduced with lentiviruses encoding the indicated sgRNAs. n = 3 experiments.

(D) Expression of *PDGFRA*, measured by qPCR, in GFP-positive SU-DIPG-XXXVI and HSJD-DIPG-007 cells transduced with lentiviruses encoding the indicated sgRNAs. n = 3 experiments.

(legend continued on next page)

inactivate them individually and in combination in *ACVR1* mutant (SU-DIPG-IV, SU-DIPG-XXI, SU-DIPG-XXXVI, HSJD-DIPG-007) or wild-type (SU-DIPG-VI) cells. We used LentiCRISPRv2-GFP lentiviruses encoding Cas9, GFP, and sgRNAs targeting *ASCL1* or *SOX11*, and confirmed proper editing of the targeted loci in infected cells (Figure S5C). We then compared the relative fitness of CRISPR-edited cells with their non-edited neighbors by tracking the proportion of GFP-expressing cells over time (Figure 5B). In cultures transduced with lentiviruses encoding sgRNAs targeting *ASCL1*, *SOX11*, or both, but not a control sgRNA, the representation of GFP^{+} cells decreased with successive passages (Figures 5C, S5D, and S5E).

Because our transcriptomic data indicated that *Ascl1* and *Sox11* upregulation in $Acvr1^{floxG328V/+}$;$Olig2^{Cre/+}$ mice occurred concurrently with oligodendrocyte differentiation arrest, we measured the effect of *ASCL1* and *SOX11* inactivation on *PDGFRA* expression in DIPG cells. Individual or combined CRISPR-mediated targeting of *ASCL1* and *SOX11* was associated with a reduction in *PDGFRA* expression in SU-DIPG-XXXVI and HSJD-DIPG-007 cells (Figure 5D). To assess the role of ASCL1 and SOX11 in regulating DIPG cell tumorigenicity *in vivo*, we xenografted CRISPR-edited SU-DIPG-XXXVI, or HSJD-DIPG-007, cells in the brainstem of newborn NSG mice. Compared with mice transplanted with cells transduced with LentiCRISPRv2-GFP encoding a control sgRNA, animals xenografted with *ASCL1* and *SOX11* gene-edited cells survived longer (Figure 5E), which was associated with reduced tumor PDGFRA expression at endpoint (Figure 5F). Here, these data demonstrate that ASCL1 and SOX11 control DIPG cell fitness and tumorigenicity.

Characterization of E6201 as an ACVR1 Inhibitor

Because differentiation-arrested cells frequently drive the growth of gliomas (Lan et al., 2017; Tirosh et al., 2016), ACVR1 might be a valuable therapeutic target. E6201 is a covalent inhibitor of MEK1/2, which are effectors of PDGFRA signaling (Goto et al., 2009). It is currently in a phase 1 clinical trial for CNS metastases in BRAF/MEK-mutant melanoma (Babiker et al., 2019; Tibes et al., 2018). Using a cellular NanoBRET target engagement assay, we unexpectedly identified binding between E6201 and ACVR1 (Figure S6A, half maximal inhibitory concentration [IC_{50}] $\approx$ 0.25 μM). In cells, E6201 dose-dependently inhibited the activation of a BMP-responsive reporter (BRE-Luc) by exogenous BMP2, BMP6, or BMP9 (Figures 6A and S6B). This effect was specific to the BMP pathway, as E6201 only poorly inhibited a transforming growth factor β (TGF-β)-dependent CAGA-Luc reporter (Figure S6C, IC_{50} > 10 μM). Consistently, E6201 blocked BMP ligand-stimulated phosphorylation of SMAD1 (Figure 6B). To identify which BMP receptors might be most effectively targeted by E6201, cells were transfected with constructs encoding constitutively active (ca-) versions of ACVR1, BMPR1A, or BMPR1B, all of which stimulated *BRE*-Luc activity and SMAD1 phosphorylation to comparable levels (Figures S6D and S6E). E6201 dose-dependently inhibited BMP pathway activation induced by ca-ACVR1, whereas its effects on ca-BMPR1A and ca-BMPR1B were more modest (Figures S6D and S6E). In addition, E6201 had a larger suppressive effect on pathway activation induced by mutant ACVR1 than by wild-type ACVR1 (Figure S6F). These data suggested that E6201 preferentially inhibits ACVR1 among type I BMP receptors and is effective against mutant ACVR1.

E6201 is an ATP-competitive inhibitor that targets MEK1 through covalent binding to Cys207 (PDB: 5HZE). In ACVR1, this cysteine residue is replaced by Ala353, preventing a comparable covalent interaction. Therefore, to obtain mechanistic insights into the inhibitory effect of E6201 on ACVR1, we solved the 1.5-Å structure of ACVR1 in complex with its interacting partner FKBP12 and E6201 by X-ray crystallography (Figure 6C; Table S3). E6201 occupies the ATP-binding pocket of ACVR1 with a binding position similar to that in the equivalent MEK1 complex (Figures S6G–S6J). Both MEK1 and ACVR1 form common hydrogen bonds to E6201 through the kinase hinge (for ACVR1, at His286) and catalytic loop regions (for ACVR1, at Lys340). The missing covalent linkage in ACVR1 is compensated for by a van der Waals interaction involving Ala353 as well as two additional hydrogen bonds: one involving the threonine gatekeeper residue (Thr283) and the other involving the catalytic lysine (Lys235), which is displaced in MEK1 by its distinct αC-out, DFG-out conformation. Further structures of BMPR1B suggest that this kinase favors a more collapsed conformation of the ATP-binding pocket that would disfavor the binding of E6201, potentially explaining the observed selectivity of E6201 for ACVR1 over other BMP receptors (Figure S6K).

In primary brainstem glial cells, E6201 blunted basal and $Acvr1^{G328V}$-stimulated *Id1* and *Id3*, but not *Id2*, gene expression (Figures 6D and S6L). In the presence of the BMP ligand antagonist noggin, which by itself did not prevent $Acvr1^{G328V}$-stimulated gene expression, E6201 completely blocked $Acvr1^{G328V}$-induced *Id2* upregulation (Figure S6L). Noggin and E6201 also had additive effects on *Id1* and *Id3* expression (Figures 6D and S6L). Taken together, these data suggest that E6201 can inhibit hyperactive BMP signaling downstream of mutant ACVR1.

E6201 Inhibits DIPG Cell Growth and Delays Tumor Progression *In Vivo*

Inhibition of both MEK1/2 and mutant ACVR1 suggested that E6201 may show activity against DIPG tumor cells. Indeed, E6201 dose dependently reduced the growth or viability of DIPG cell lines carrying *ACVR1* and *HIST1H3B* or *H3F3A* mutations (SU-DIPG-IV, SU-DIPG-XXI, SU-DIPG-XXXVI, HSJD-DIPG-007), while an $ACVR1^{WT}$ cell line, SU-DIPG-VI, was less sensitive (Figure 7A). E6201 had a similar effect on tumor cells derived from spontaneous $Acvr1^{G328V}$ mouse gliomas

(E) Left: bright field and GFP fluorescence stereoscopic microscope images of an NSG mouse brain xenografted with LentiCRISPRv2-GFP-transduced SU-DIPG-XXXVI cells. Right: survival curves of NSG mice xenografted with 2 × 10^5 SU-DIPG-XXXVI or HSJD-DIPG-007 cells transduced with lentiviruses encoding the indicated sgRNAs.

(F) PDGFRA immunohistochemistry in HSJD-DIPG-007 tumors at endpoint. Scale bars, 50 μm.

In all panels, mean + SEM is shown. *p < 0.05, **p < 0.01, ***p < 0.001; assessed by linear regression and slope comparisons (C), repeated-measures ANOVA with Tukey multiple comparisons test (D), or Mantel-Cox test (E). See also Figure S5 and Table S2.

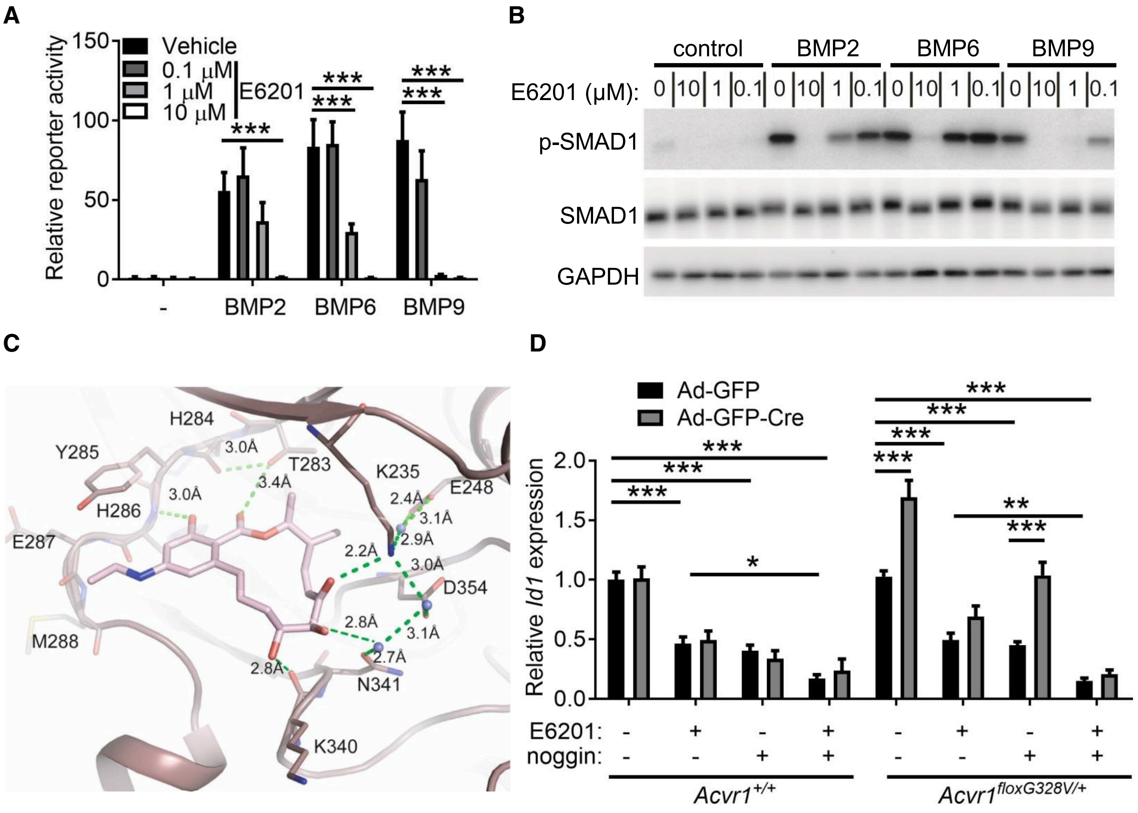

Figure 6. Characterization of E6201 as an ACVR1 Inhibitor

(A) Luciferase activity in lysates from C2C12 cells transfected with the BRE-Luc reporter and stimulated overnight with 25 ng/mL BMP2, 25 ng/mL BMP6, or 5 ng/mL BMP9, and the indicated concentrations of E6201. n = 3 experiments.

(B) Western blot of lysates from C2C12 cell stimulated for 45 min with 25 ng/mL BMP2, 25 ng/mL BMP6, or 5 ng/mL BMP9, with the indicated concentrations for E6201 applied 45 min before BMP ligand addition, probed with the indicated antibodies.

(C) X-ray crystal structure showing interactions of E6201 (pink) in the ATP-binding pocket of ACVR1 (brown). Bound waters are shown as blue spheres. Hydrogen bonds are indicated by green dashed lines. Parts of strands β1 and β2 are omitted for clarity.

(D) Expression of *Id1*, measured by qPCR, in *Acvr1*$^{+/+}$ or *Acvr1*$^{floxG328V/+}$ glial cells transduced with Ad-GFP or Ad-GFP-Cre, and treated where indicated with 100 ng/mL noggin and/or 1 μM E6201. n = 3 experiments.

In all panels, mean + SEM is shown. *p < 0.05, **p < 0.01, ***p < 0.001; assessed by repeated-measures ANOVA with Tukey multiple comparisons tests. See also Figure S6 and Table S3.

(Figure S7A). In *ACVR1* mutant DIPG cells, the effect of E6201 did not strictly correlate with that of the selective MEK inhibitor Trametinib, in agreement with the predicted distinct activities of the two compounds (Figure 7A). E6201 decreased endogenous p-SMAD1 and p-ERK1/2 levels, consistent with dual inhibition of ACVR1 and MEK1/2, whereas the ACVR1/BMPR1A/B inhibitor LDN-193189 affected only p-SMAD1, and Trametinib affected only p-ERK1/2 (Figure 7B). Accordingly, both E6201 and LDN-193189 robustly reduced *ID1* and *ID3* expression (Figure 7C), whereas *ID2* expression was largely unchanged, similar to the results in primary mouse glial cell cultures (Figure 6L). E6201 exerted its effects on DIPG cells at least in part by inducing apoptosis, unlike LDN-193189 (Figure 7D).

To assess the extent to which E6201 acts on DIPG cells by inhibiting ACVR1, we examined how the response to E6201 is affected by ablating the *ACVR1* gene. CRISPR/Cas9-mediated *ACVR1* gene inactivation impaired the growth or viability of SU-DIPG-XXXVI and HSJD-DIPG-007 cells (Figure S7B), but also blunted the effect of E6201 (Figure 7E). These results are consistent with E6201 acting partly through inhibition of ACVR1 in DIPG cells. Given the strong association between *ACVR1* and *PIK3CA* mutations in DIPG, we evaluated the effect of combined treatment with E6201 and the brain-penetrant PI3K inhibitor Buparlisib (de Gooijer et al., 2018). By itself, Buparlisib had comparable dose-dependent inhibitory effects on cell growth or viability on all the DIPG cell lines tested (Figure S7C). In SU-DIPG-XXXVI and HSJD-DIPG-007 cells, E6201 and Buparlisib had mostly additive effects, with modest synergy around the IC_{30}–IC_{50} concentrations for both compounds (Figure S7D).

E6201 shows good brain penetration, when administered peripherally in mice (Gampa et al., 2018). To further test the potential therapeutic utility of E6201, we examined its effect on survival in immuno-compromised mice xenografted with SU-DIPG-XXXVI or HSJD-DIPG-007 tumor cells. Mice were transplanted with 2×10^5 tumor cells in the brainstem area at postnatal day 2, and injected intraperitoneally with 40 mg/kg E6201, or vehicle control, every other day, starting at postnatal day 15 (Figure 7F). In both xenograft models, E6201 prolonged

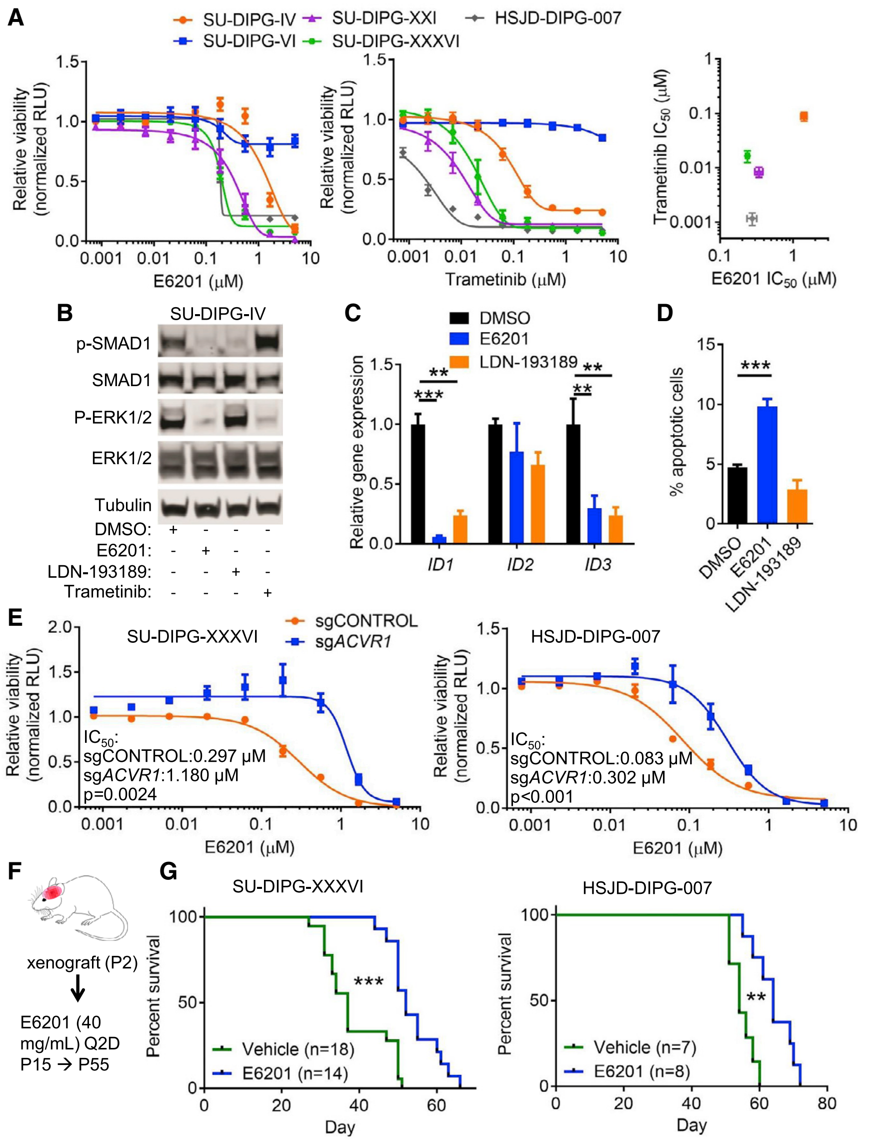

Figure 7. E6201 Impairs *ACVR1* Mutant DIPG Cell Viability and Tumorigenicity

(A) Relative ATP-dependent luminescence activity in the indicated cell lines, exposed to increasing concentrations of E6201 (left), or Trametinib (middle). n = 3 experiments. Right: E6201 and Trametinib IC_{50} in *ACVR1* mutant cell lines.

(B) Western blot of lysates from SU-DIPG-IV cells treated for 24 h with DMSO, 2 μM E6201, 1 μM LDN-193189, or 0.1 μM Trametinib, probed the indicated antibodies.

(C) Expression of *ID1*, *ID2*, and *ID3*, measured by qPCR, in SU-DIPG-IV cells treated for 24 h with DMSO, 2 μM E6201, or 1 μM LDN-193189. n = 3 experiments.

(D) Percentage of AnnexinV-positive cells in SU-DIPG-IV cells treated for 48 h with DMSO, 2 μM E6201, or 1 μM LDN-193189. n = 3 experiments.

(E) Relative ATP-dependent luminescence activity in SU-DIPG-XXXVI (left) or HSJD-DIPG-007 (right) cells transduced with lentiviruses encoding the indicated sgRNAs, and exposed to increasing concentrations of E6201. Data were normalized to the vehicle-treated condition for each sgRNA. n = 3 experiments.

(F) Experimental design and treatment protocol for assessment of E6201 in DIPG xenograft mouse models.

(G) Survival curves of NSG mice xenografted at postnatal day 2 with 2×10^5 SU-DIPG-XXXVI (left), or HSJD-DIPG-007 (right) cells, treated with E6201 or vehicle control (30% Captisol).

In all panels, mean + SEM is shown. $^*p < 0.05$, $^{**}p < 0.01$, $^{***}p < 0.001$; assessed by repeated-measures ANOVA with Tukey multiple comparisons tests (C and D), nonlinear regression analysis (E) or Mantel-Cox tests (G). See also Figure S7.

survival (Figure 7G), demonstrating efficacy of the drug toward DIPG tumor cells *in vivo*.

DISCUSSION

By showing that mutant ACVR1 is sufficient to arrest the differentiation of oligodendroglial lineage cells (Figure 8), our results provide mechanistic explanations for the presence of *ACVR1* mutations among the earliest oncogenic events in a subset of DIPG, and for the recent discovery that DIPG malignant cells harbor features of OPCs (Filbin et al., 2018). By itself, the differentiation block induced by mutant ACVR1 does not appear to be sufficient to induce tumors. Indeed, patients carrying germline *ACVR1* mutations that overlap with those found in DIPG develop fibrodysplasia ossificans progressiva (FOP), but not DIPG (Taylor et al., 2014b). Nevertheless, FOP patients can harbor brainstem lesions that resemble hamartomas (Severino et al., 2016), and focal demyelinating lesions (Kan et al., 2012). We observed that, similar to humans, the combination of $Acvr1^{G328V}$, $Hist1h3b^{K27M}$, and $Pik3ca^{H1047R}$ led to the development of high-grade diffuse gliomas in mice (Figure 8). These spontaneous tumors spread over large areas of the midbrain and forebrain, but rarely involved the brainstem. In contrast, in humans, tumors harboring the cognate mutations preferentially arise in the pons (Mackay et al., 2017). Still, our results clearly demonstrated collaboration between $Acvr1^{G328V}$ and $Pik3ca^{H1047R}$ in driving tumorigenesis, faithfully recapitulating the synergy predicted by human genetic data. The localization of $Acvr1^{G328V}$;$Pik3ca^{H1047R}$ mutant tumors in our mouse model may have been influenced by the cell type targeted, although lineage tracing indicated that the $Olig2^{Cre}$ allele is active in the ventral brainstem. Alternatively, interspecies biological differences may dictate anatomical preferences for tumor development.

In our mouse model, $Hist1h3b^{K27M}$ was not strictly necessary for the emergence of tumors. Although some studies have shown a role for H3-K27M in glioma occurrence and progression in mice, a stringent requirement for mutant histones for tumorigenesis has not been consistently demonstrated (Hoeman et al., 2019; Larson et al., 2019; Pathania et al., 2017). Therefore, despite clear genetic and functional evidence supporting a

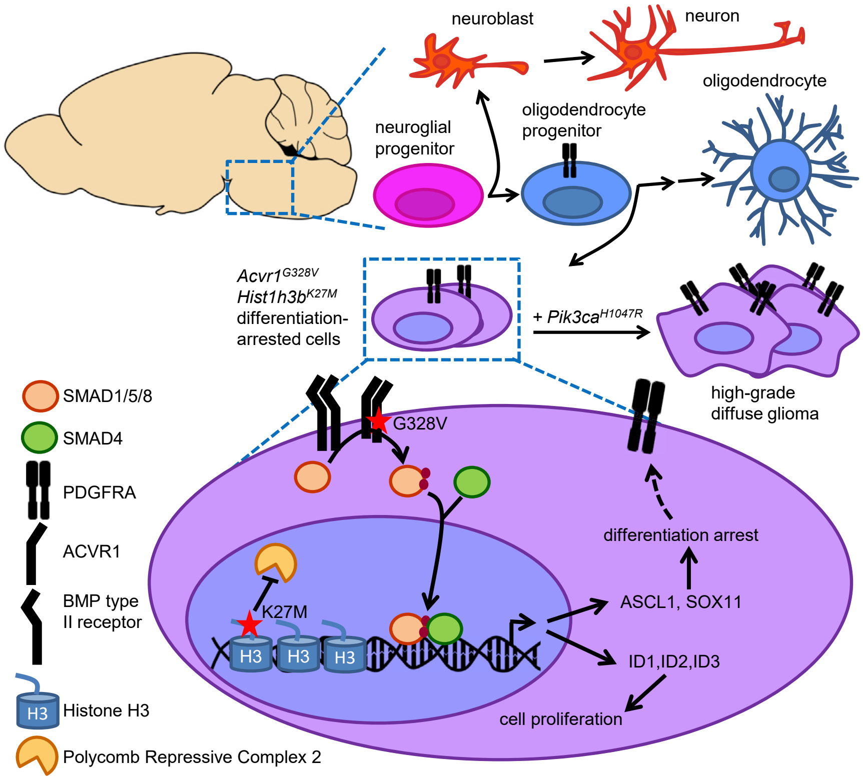

Figure 8. Mutant *ACVR1* Arrests Glial Cell Differentiation to Drive Tumorigenesis

Model depicting the effects of *Acvr1*^{G328V}, alone or together with *Hist1h3b*^{K27M}, on the expression of ID1/2/3, ASCL1, SOX11, and PDGFRA, and downstream consequences on cellular proliferation and differentiation. PDGFRA induction downstream of ASCL1 and SOX11 may be direct or indirect (broken arrow). The effect of combined *Acvr1*^{G328V}, *Hist1h3b*^{K27M}, and *Pik3ca*^{H1047R} on tumor emergence and progression is illustrated.

driving role for H3-K27M mutations in the formation, progression, and maintenance of high-grade diffuse gliomas in humans (Harutyunyan et al., 2019; Silveira et al., 2019), whether and how this can be recapitulated in mice remains to be clarified. In our knockin model, an effect of *Hist1h3b*^{K27M} on brainstem H3-K27me3 levels was seen soon after birth, but was largely abolished in young adult mice. *Hist1h3b* encodes a replication-dependent histone, and therefore the effects of *Hist1h3b*^{K27M} may depend on cell division. The robust expansion of OPCs in the mouse brainstem that occurs after birth largely subsides by the end of the second postnatal week (Lindquist et al., 2016), potentially explaining temporally restricted effects of the *Hist1h3b*^{K27M} mutation. In humans, the corresponding developmental "window of opportunity" may remain open for months or years, favoring tumor emergence. In our model, we observed very mild effects of *Hist1h3b*^{K27M} on gene expression and cell proliferation, consistent with observations reported in other *in vitro* contexts (Larson et al., 2019). The mechanisms whereby H3-K27M drives tumorigenesis undoubtedly go beyond effects on cell proliferation (Harutyunyan et al., 2019; Larson et al., 2019). Because cells with OPC characteristics are seen in both *ACVR1*-wild-type and *ACVR1*-mutant tumors (Filbin et al., 2018), H3-K27M mutations likely play a central role in differentiation arrest, as suggested previously (Funato et al., 2014; Weinberg et al., 2017). Understanding how H3-K27M and *ACVR1* mutations cooperate to impose an OPC-like phenotype on DIPG cells is an important topic for future investigations.

Our study has uncovered several potential effectors of hyperactive signaling downstream of mutant ACVR1, including ID1/2/3, as well as the transcription factors ASCL1 and SOX11 (Figure 8). ID2 has previously been identified as a key mediator of glioblastoma cell "stemness" (Lee et al., 2016). Furthermore, a subset of *ACVR1*-wild-type DIPG tumors harbors amplifications of the *ID2* gene (Buczkowicz et al., 2014; Mackay et al., 2017), positioning ID2 as a possible key effector of mutant ACVR1. Our identification of ASCL1 as a candidate mediator of *Acvr1*^{G328V}-induced oligodendroglial lineage differentiation arrest was particularly intriguing. ASCL1 has well-described pro-neural functions (Bertrand et al., 2002), and enhanced ASCL1 expression can impair adult glioblastoma tumorigenicity by promoting neuronal-like differentiation (Park et al., 2017). Our data show that increased *Ascl1* expression in *Acvr1*^{floxG328V};*Olig2*^{Cre} mice is associated with upregulation of certain neuroblast, but not mature neuronal, markers. Furthermore, ASCL1 is required for OPC formation (Nakatani et al., 2013), is robustly downregulated during oligodendroglial cell maturation (Cahoy et al., 2008; Dugas et al., 2008; Swiss et al., 2011), and is highly expressed in most DIPG cells (Filbin et al., 2018). Moreover, ectopic ASCL1 expression in adult glioblastoma inhibits some aspects of glial differentiation (Park et al., 2017). Therefore, ASCL1's function in gliomas likely depends on its expression levels and on the presence of other factors that modulate its activity, and could contribute to locking cells into a state characterized by properties of both neuronal and glial progenitors.

Understanding the molecular mechanisms of tumor initiation and progression is crucial to design therapeutic strategies that can efficiently inhibit or reverse tumor growth. Our data suggest that pharmacological targeting of mutant ACVR1 and/or processes related to OPC differentiation arrest, such as PDGFRA signaling, may be promising therapeutic strategies to suppress or reverse the fundamental processes that drive DIPGs. In this context, our characterization of E6201, a drug already known to target MEK1, as an ACVR1 inhibitor that can impair DIPG cell growth and viability, may be particularly interesting. Notably, E6201 can achieve good brain exposure in mice (Gampa et al., 2018), and we demonstrated here that it prolongs survival in DIPG brain xenograft models. In addition, E6201 has been well tolerated in phase I trials in human patients with solid tumors (Tibes et al., 2018). Our results support further exploration of E6201, and similar drugs that can target ACVR1 and/or PDGFRA signaling, as agents for the treatment of DIPGs.

STAR★METHODS

Detailed methods are provided in the online version of this paper and include the following:

- KEY RESOURCES TABLE
- LEAD CONTACT AND MATERIALS AVAILABILITY
- EXPERIMENTAL MODEL AND SUBJECT DETAILS
  - *In Vivo* Animal Studies
  - Human Studies
  - Cell Lines and Primary Cultures
  - Studies Using Organisms as Source for Materials Used in Experiments
- METHOD DETAILS
  - Mice
  - Patient-Derived DIPG Cell Lines
  - CRISPR/Cas9 Gene Editing in Patient-Derived DIPG Cells Lines
  - Neural Stem Cell Culture and Derivation of Mouse Tumor Cell Lines
  - Lentivirus Transduction of Neural Stem Cells, and Neurosphere-Forming Assays
  - Primary Brainstem Glial Cell Culture and Adenovirus Infection
  - C2C12 and HEK-293 Cells Culture
  - Drugs
  - Xenograft Models
  - RNA and Protein Extraction
  - Western Blotting
  - cDNA Synthesis and Quantitative PCR
  - RNA-Sequencing and Analysis
  - Cell Growth and Viability Assays
  - NanoBRET Assays
  - Reporter Assays
  - X-Ray Crystallography
  - Histology
  - Chromatin Immunoprecipitation
  - Flow Cytometry and Cell Sorting
- QUANTIFICATION AND STATISTICAL ANALYSIS
- DATA AND CODE AVAILABILITY

SUPPLEMENTAL INFORMATION

Supplemental Information can be found online at https://doi.org/10.1016/j.ccell.2020.02.002.

ACKNOWLEDGMENTS

This study was supported by a grant from The Cure Starts Now (United States) to T.W.M., J.F., D.S., and C.H., a Canadian Institutes of Health Research (CIHR, Canada) Foundation Grant to T.W.M., and a Young Investigator Grant from the Alex's Lemonade Stand Foundation (United States) to J.F. J.F. was supported by fellowships from the Fonds de Recherche du Québec - Santé (FRSQ, Canada), and from the CIHR, as well as a Scholarships for the Next Generation of Scientists from the Cancer Research Society (Canada). E6201 was generously provided by Thomas Myers and Spirita Oncology (United States). D.S. is supported by an OICR Brain Translational Research Initiative (Canada). P.t.D. is supported by Cancer Genomics Center Netherlands (Netherlands). G.S.-D. is sponsored by the Netherlands Cardiovascular Research Initiative (Netherlands): the Dutch Heart Foundation, Dutch Federation of University Medical Centers, the Netherlands Organization for Health Research and Development, and the Royal Netherlands Academy of Sciences (RECONNECT consortium). E.W. and A.N.B. acknowledge funding from FOP Friends (United Kingdom). J.F.W. and A.N.B. acknowledge funding from The Brain Tumour Charity (United Kingdom). The authors would also like to thank Diamond Light Source for beamtime (proposal mx15433), as well as the staff of beamline I03 for assistance with crystal testing and data collection. The SGC is a registered charity (number 1097737) that receives funds from AbbVie (United States), Bayer Pharma AG (Germany), Boehringer Ingelheim (Germany), Canada Foundation for Innovation (Canada), Eshelman Institute for Innovation (United States), Genome Canada (Canada), Innovative Medicines Initiative (EU/EFPIA) (ULTRA-DD grant no. 115766) (European Union), Janssen (Belgium), Merck KGaA (Germany), MSD (United States), Novartis Pharma AG (Switzerland), Ontario Ministry of Economic Development and Innovation (Canada), Pfizer, São Paulo Research Foundation-FAPESP (Brazil), Takeda (Japan), and Wellcome (106169/ZZ14/Z) (United Kingdom). The authors thank the Princess Margaret Genomics Centre, the HistoCore, the Advanced Optical Microscopy Facility, and the Animal Resource Centre at Princess Margaret Hospital, for support on various aspects of the studies. The authors also thank the laboratories of Drs Michelle Monje and Ángel Montero Carcaboso for the gift of DIPG cell lines and patient samples for histology.

AUTHOR CONTRIBUTIONS

J.F. conceived and supervised most of the study, generated mice, designed and performed experiments, analyzed the results, and wrote the manuscript with inputs from all the authors. R.T., G.H., I.Z., and A.W. performed experiments and analyzed the results. A.N.B. supervised the crystallography and NanoBRET experiments. E.W. and A.N.B. performed X-ray crystallography studies, analyzed the results, and contributed to writing the manuscript. G.S.-D., M.T., and P.t.D. characterized the inhibitory effect of E6201 on ACVR1, analyzed the results, and contributed to writing the manuscript. J.F.W. performed the NanoBRET and TGF-β inhibition experiments, and analyzed the results. P.R. analyzed RNA-seq results from mice. R.S. and C.H. acquired and analyzed RNA-seq data from human tumors. L.N.A. and D.J.G. generated and analyzed *Acvr1*^{floxR206H}*;Pdgfra-Cre* mutant mice. A.W., J.H., A.Y.-T., and B.E.S. assisted with designing mutant alleles and generated mouse lines. D.S. and C.H. provided expertize, guidance, and novel tools and reagents for many aspects of the project, and contributed to writing the manuscript. T.W.M. supervised the study and contributed to writing the manuscript. All the authors revised and approved the manuscript.

DECLARATIONS OF INTERESTS

The authors declare no competing interests.

Received: July 10, 2019
Revised: December 2, 2019
Accepted: February 4, 2020
Published: March 5, 2020

REFERENCES

Adams, P.D., Afonine, P.V., Bunkoczi, G., Chen, V.B., Davis, I.W., Echols, N., Headd, J.J., Hung, L.W., Kapral, G.J., Grosse-Kunstleve, R.W., et al. (2010). PHENIX: a comprehensive Python-based system for macromolecular structure solution. Acta Crystallogr. D Biol. Crystallogr. *66*, 213–221.

Babiker, H.M., Byron, S.A., Hendricks, W.P.D., Elmquist, W.F., Gampa, G., Vondrak, J., Aldrich, J., Cuyugan, L., Adkins, J., De Luca, V., et al. (2019). E6201, an intravenous MEK1 inhibitor, achieves an exceptional response in BRAF V600E-mutated metastatic malignant melanoma with brain metastases. Invest. New Drugs *37*, 636–645.

Bardeesy, N., Sinha, M., Hezel, A.F., Signoretti, S., Hathaway, N.A., Sharpless, N.E., Loda, M., Carrasco, D.R., and DePinho, R.A. (2002). Loss of the Lkb1 tumour suppressor provokes intestinal polyposis but resistance to transformation. Nature *419*, 162–167.

Basak, O., Krieger, T.G., Muraro, M.J., Wiebrands, K., Stange, D.E., Frias-Aldeguer, J., Rivron, N.C., van de Wetering, M., van Es, J.H., et al. (2018). Troy+ brain stem cells cycle through quiescence and regulate their number by sensing niche occupancy. Proc. Natl. Acad. Sci. U S A *115*, E610–E619.

Bender, S., Tang, Y., Lindroth, A.M., Hovestadt, V., Jones, D.T., Kool, M., Zapatka, M., Northcott, P.A., Sturm, D., Wang, W., et al. (2013). Reduced H3K27me3 and DNA hypomethylation are major drivers of gene expression in K27M mutant pediatric high-grade gliomas. Cancer Cell *24*, 660–672.

Bertrand, N., Castro, D.S., and Guillemot, F. (2002). Proneural genes and the specification of neural cell types. Nat. Rev. Neurosci. *3*, 517–530.

Bolger, A., M., Lohse, M., and Usadel, B. (2014). Trimmomatic: a flexible trimmer for illumina sequence data. Bioinformatics *30*, 2114–2120.

Buczkowicz, P., Hoeman, C., Rakopoulos, P., Pajovic, S., Letourneau, L., Dzamba, M., Morrison, A., Lewis, P., Bouffet, E., Bartels, U., et al. (2014). Genomic analysis of diffuse intrinsic pontine gliomas identifies three molecular subgroups and recurrent activating ACVR1 mutations. Nat. Genet. *46*, 451–456.

Cahoy, J.D., Emery, B., Kaushal, A., Foo, L.C., Zamanian, J.L., Christopherson, K.S., Xing, Y., Lubischer, J.L., Krieg, P.A., Krupenko, S.A., et al. (2008). A transcriptome database for astrocytes, neurons, and oligodendrocytes: a new resource for understanding brain development and function. J. Neurosci. *28*, 264–278.

Carter, C.S., Vogel, T.W., Zhang, Q., Seo, S., Swiderski, R.E., Moninger, T.O., Cassell, M.D., Thedens, D.R., Keppler-Noreuil, K.M., Nopoulos, P., et al. (2012). Abnormal development of NG2+PDGFR-alpha+ neural progenitor cells leads to neonatal hydrocephalus in a ciliopathy mouse model. Nat. Med. *18*, 1797–1804.

Carvalho, D., Taylor, K.R., Olaciregui, N.G., Molinari, V., Clarke, M., Mackay, A., Ruddle, R., Henley, A., Valenti, M., Hayes, A., et al. (2019). ALK2 inhibitors display beneficial effects in preclinical models of ACVR1 mutant diffuse intrinsic pontine glioma. Commun. Biol. *2*, 156.

Chaikuad, A., Alfano, I., Kerr, G., Sanvitale, C.E., Boergermann, J.H., Triffitt, J.T., von Delft, F., Knapp, S., Knaus, P., and Bullock, A.N. (2012). Structure of the bone morphogenetic protein receptor ALK2 and implications for fibrodysplasia ossificans progressiva. J. Biol. Chem. *287*, 36990–36998.

Chan, K.M., Fang, D., Gan, H., Hashizume, R., Yu, C., Schroeder, M., Gupta, N., Mueller, S., James, C.D., Jenkins, R., et al. (2013). The histone H3.3K27M mutation in pediatric glioma reprograms H3K27 methylation and gene expression. Genes Dev. *27*, 985–990.

Davis, I.W., Leaver-Fay, A., Chen, V.B., Block, J.N., Kapral, G.J., Wang, X., Murray, L.W., Arendall, W.B., 3rd, Snoeyink, J., Richardson, J.S., and Richardson, D.C. (2007). MolProbity: all-atom contacts and structure validation for proteins and nucleic acids. Nucleic Acids Res. *35*, W375–W383.

de Gooijer, M.C., Zhang, P., Buil, L.C.M., Citirikkaya, C.H., Thota, N., Beijnen, J.H., and van Tellingen, O. (2018). Buparlisib is a brain penetrable pan-PI3K inhibitor. Sci. Rep. *8*, 10784.

Dobin, A., Davis, C.A., Schlesinger, F., Drenkow, J., Zaleski, C., Jha, S., Batut, P., Chaisson, M., and Gingeras, T.R. (2013). STAR: ultrafast universal RNA-seq aligner. Bioinformatics *29*, 15–21.

Dugas, J.C., Mandemakers, W., Rogers, M., Ibrahim, A., Daneman, R., and Barres, B.A. (2008). A novel purification method for CNS projection neurons leads to the identification of brain vascular cells as a source of trophic support for corticospinal motor neurons. J. Neurosci. *28*, 8294–8305.

Emsley, P., and Cowtan, K. (2004). Coot: model-building tools for molecular graphics. Acta Crystallogr. D Biol. Crystallogr. *60*, 2126–2132.

Ewels, P., Magnusson, M., Lundin, S., and Kaller, M. (2016). MultiQC: summarize analysis results for multiple tools and samples in a single report. Bioinformatics *32*, 3047–3048.

Filbin, M.G., Tirosh, I., Hovestadt, V., Shaw, M.L., Escalante, L.E., Mathewson, N.D., Neftel, C., Frank, N., Pelton, K., Hebert, C.M., et al. (2018). Developmental and oncogenic programs in H3K27M gliomas dissected by single-cell RNA-seq. Science *360*, 331–335.

Fontebasso, A.M., Papillon-Cavanagh, S., Schwartzentruber, J., Nikbakht, H., Gerges, N., Fiset, P.O., Bechet, D., Faury, D., De Jay, N., Ramkissoon, L.A., et al. (2014). Recurrent somatic mutations in ACVR1 in pediatric midline high-grade astrocytoma. Nat. Genet. *46*, 462–466.

Fujii, M., Takeda, K., Imamura, T., Aoki, H., Sampath, T.K., Enomoto, S., Kawabata, M., Kato, M., Ichijo, H., and Miyazono, K. (1999). Roles of bone morphogenetic protein type I receptors and Smad proteins in osteoblast and chondroblast differentiation. Mol. Biol. Cell *10*, 3801–3813.

Funato, K., Major, T., Lewis, P.W., Allis, C.D., and Tabar, V. (2014). Use of human embryonic stem cells to model pediatric gliomas with H3.3K27M histone mutation. Science *346*, 1529–1533.

Gadi, J., Jung, S.H., Lee, M.J., Jami, A., Ruthala, K., Kim, K.M., Cho, N.H., Jung, H.S., Kim, C.H., and Lim, S.K. (2013). The transcription factor protein Sox11 enhances early osteoblast differentiation by facilitating proliferation and the survival of mesenchymal and osteoblast progenitors. J. Biol. Chem. *288*, 25400–25413.

Gampa, G., Kim, M., Cook-Rostie, N., Laramy, J.K., Sarkaria, J.N., Paradiso, L., DePalatis, L., and Elmquist, W.F. (2018). Brain distribution of a novel MEK inhibitor E6201: implications in the treatment of melanoma brain metastases. Drug Metab. Dispos. *46*, 658–666.

Goto, M., Chow, J., Muramoto, K., Chiba, K., Yamamoto, S., Fujita, M., Obaishi, H., Tai, K., Mizui, Y., Tanaka, I., et al. (2009). E6201 [(3S,4R,5Z,8S,9S,11E)-14-(ethylamino)-8, 9,16-trihydroxy-3,4-dimethyl-3,4,9,19-tetrahydro-1H-2-benzoxacyclotetradecine-1,7 (8H)-dione], a novel kinase inhibitor of mitogen-activated protein kinase/extracellular signal-regulated kinase kinase (MEK)-1 and MEK kinase-1: in vitro characterization of its anti-inflammatory and antihyperproliferative activities. J. Pharmacol. Exp. Ther. *331*, 485–495.

Grasso, C.S., Tang, Y., Truffaux, N., Berlow, N.E., Liu, L., Debily, M.A., Quist, M.J., Davis, L.E., Huang, E.C., Woo, P.J., et al. (2015). Functionally defined therapeutic targets in diffuse intrinsic pontine glioma. Nat. Med. *21*, 555–559.

Harutyunyan, A.S., Krug, B., Chen, H., Papillon-Cavanagh, S., Zeinieh, M., De Jay, N., Deshmukh, S., Chen, C.C.L., Belle, J., Mikael, L.G., et al. (2019). H3K27M induces defective chromatin spread of PRC2-mediated repressive H3K27me2/me3 and is essential for glioma tumorigenesis. Nat. Commun. *10*, 1262.

Hatsell, S.J., Idone, V., Wolken, D.M., Huang, L., Kim, H.J., Wang, L., Wen, X., Nannuru, K.C., Jimenez, J., Xie, L., et al. (2015). ACVR1R206H receptor mutation causes fibrodysplasia ossificans progressiva by imparting responsiveness to activin A. Sci. Transl. Med. *7*, 303ra137.

Hino, K., Ikeya, M., Horigome, K., Matsumoto, Y., Ebise, H., Nishio, M., Sekiguchi, K., Shibata, M., Nagata, S., Matsuda, S., and Toguchida, J. (2015). Neofunction of ACVR1 in fibrodysplasia ossificans progressiva. Proc. Natl. Acad. Sci. U S A *112*, 15438–15443.

Hoeman, C.M., Cordero, F.J., Hu, G., Misuraca, K., Romero, M.M., Cardona, H.J., Nazarian, J., Hashizume, R., McLendon, R., Yu, P., et al. (2019). ACVR1 R206H cooperates with H3.1K27M in promoting diffuse intrinsic pontine glioma pathogenesis. Nat. Commun. *10*, 1023.

Hoffman, L.M., DeWire, M., Ryall, S., Buczkowicz, P., Leach, J., Miles, L., Ramani, A., Brudno, M., Kumar, S.S., Drissi, R., et al. (2016). Spatial genomic heterogeneity in diffuse intrinsic pontine and midline high-grade glioma: implications for diagnostic biopsy and targeted therapeutics. Acta Neuropathol. Commun. *4*, 1.

Ianevski, A., He, L., Aittokallio, T., and Tang, J. (2017). SynergyFinder: a web application for analyzing drug combination dose-response matrix data. Bioinformatics *33*, 2413–2415.

Jones, C., and Baker, S.J. (2014). Unique genetic and epigenetic mechanisms driving paediatric diffuse high-grade glioma. Nat. Rev. Cancer *14*, https://doi.org/10.1038/nrc3811.

Jones, C., Karajannis, M.A., Jones, D.T.W., Kieran, M.W., Monje, M., Baker, S.J., Becher, O.J., Cho, Y.J., Gupta, N., Hawkins, C., et al. (2017). Pediatric high-grade glioma: biologically and clinically in need of new thinking. Neurooncology *19*, 153–161.

Kan, L., Kitterman, J.A., Procissi, D., Chakkalakal, S., Peng, C.Y., McGuire, T.L., Goldsby, R.E., Pignolo, R.J., Shore, E.M., Kaplan, F.S., and Kessler, J.A. (2012). CNS demyelination in fibrodysplasia ossificans progressiva. J. Neurol. *259*, 2644–2655.

Korchynskyi, O., and ten Dijke, P. (2002). Identification and functional characterization of distinct critically important bone morphogenetic protein-specific response elements in the Id1 promoter. J. Biol. Chem. *277*, 4883–4891.

Kron, K.J., Murison, A., Zhou, S., Huang, V., Yamaguchi, T.N., Shiah, Y.J., Fraser, M., van der Kwast, T., Boutros, P.C., Bristow, R.G., and Lupien, M. (2017). TMPRSS2-ERG fusion co-opts master transcription factors and activates NOTCH signaling in primary prostate cancer. Nat. Genet. *49*, 1336–1345.

Lan, X., Jorg, D.J., Cavalli, F.M.G., Richards, L.M., Nguyen, L.V., Vanner, R.J., Guilhamon, P., Lee, L., Kushida, M.M., Pellacani, D., et al. (2017). Fate mapping of human glioblastoma reveals an invariant stem cell hierarchy. Nature *549*, 227–232.

Larson, J.D., Kasper, L.H., Paugh, B.S., Jin, H., Wu, G., Kwon, C.H., Fan, Y., Shaw, T.I., Silveira, A.B., Qu, C., et al. (2019). Histone H3.3 K27M accelerates spontaneous brainstem glioma and drives restricted changes in bivalent gene expression. Cancer Cell *35*, 140–155.e7.

Lasorella, A., Benezra, R., and Iavarone, A. (2014). The ID proteins: master regulators of cancer stem cells and tumour aggressiveness. Nat. Rev. Cancer *14*, 77–91.

Lee, S.B., Frattini, V., Bansal, M., Castano, A.M., Sherman, D., Hutchinson, K., Bruce, J.N., Califano, A., Liu, G., Cardozo, T., et al. (2016). An ID2-dependent mechanism for VHL inactivation in cancer. Nature *529*, 172–177.

Lees-Shepard, J.B., Yamamoto, M., Biswas, A.A., Stoessel, S.J., Nicholas, S.E., Cogswell, C.A., Devarakonda, P.M., Schneider, M.J., Jr., Cummins, S.M., Legendre, N.P., et al. (2018). Activin-dependent signaling in fibro/adipogenic progenitors causes fibrodysplasia ossificans progressiva. Nat. Commun. *9*, 471.

Leslie, A.G. (2006). The integration of macromolecular diffraction data. Acta Crystallogr. D Biol. Crystallogr. *62*, 48–57.

Lewis, P.W., Muller, M.M., Koletsky, M.S., Cordero, F., Lin, S., Banaszynski, L.A., Garcia, B.A., Muir, T.W., Becher, O.J., and Allis, C.D. (2013). Inhibition of PRC2 activity by a gain-of-function H3 mutation found in pediatric glioblastoma. Science *340*, 857–861.

Li, B., and Dewey, C.N. (2011). RSEM: accurate transcript quantification from RNA-seq data with or without a reference genome. BMC Bioinformatics *12*, 323.

Lindquist, R.A., Guinto, C.D., Rodas-Rodriguez, J.L., Fuentealba, L.C., Tate, M.C., Rowitch, D.H., and Alvarez-Buylla, A. (2016). Identification of proliferative progenitors associated with prominent postnatal growth of the pons. Nat. Commun. *7*, 11628.

Love, M.I., Huber, W., and Anders, S. (2014). Moderated estimation of fold change and dispersion for RNA-seq data with DESeq2. Genome Biol. *15*, 550.

Mackay, A., Burford, A., Carvalho, D., Izquierdo, E., Fazal-Salom, J., Taylor, K.R., Bjerke, L., Clarke, M., Vinci, M., Nandhabalan, M., et al. (2017). Integrated molecular meta-analysis of 1,000 pediatric high-grade and diffuse intrinsic pontine glioma. Cancer Cell *32*, 520–537.e5.

Marzluff, W.F., Gongidi, P., Woods, K.R., Jin, J., and Maltais, L.J. (2002). The human and mouse replication-dependent histone genes. Genomics *80*, 487–498.

McCoy, A.J., Grosse-Kunstleve, R.W., Adams, P.D., Winn, M.D., Storoni, L.C., and Read, R.J. (2007). Phaser crystallographic software. J. Appl. Crystallogr. *40*, 658–674.

Mohammad, F., and Helin, K. (2017). Oncohistones: drivers of pediatric cancers. Genes Dev. *31*, 2313–2324.

Mohammad, F., Weissmann, S., Leblanc, B., Pandey, D.P., Hojfeldt, J.W., Comet, I., Zheng, C., Johansen, J.V., Rapin, N., Porse, B.T., et al. (2017). EZH2 is a potential therapeutic target for H3K27M-mutant pediatric gliomas. Nat. Med. *23*, 483–492.

Monje, M., Mitra, S.S., Freret, M.E., Raveh, T.B., Kim, J., Masek, M., Attema, J.L., Li, G., Haddix, T., Edwards, M.S., et al. (2011). Hedgehog-responsive candidate cell of origin for diffuse intrinsic pontine glioma. Proc. Natl. Acad. Sci. U S A *108*, 4453–4458.

Mucha, B.E., Hashiguchi, M., Zinski, J., Shore, E.M., and Mullins, M.C. (2018). Variant BMP receptor mutations causing fibrodysplasia ossificans progressiva (FOP) in humans show BMP ligand-independent receptor activation in zebrafish. Bone *109*, 225–231.

Nagaraja, S., Quezada, M.A., Gillespie, S.M., Arzt, M., Lennon, J.J., Woo, P.J., Hovestadt, V., Kambhampati, M., Filbin, M.G., Suva, M.L., et al. (2019). Histone variant and cell context determine H3K27M reprogramming of the enhancer landscape and oncogenic state. Mol. Cell *76*, 965–980.e12.

Nakatani, H., Martin, E., Hassani, H., Clavairoly, A., Maire, C.L., Viadieu, A., Kerninon, C., Delmasure, A., Frah, M., Weber, M., et al. (2013). Ascl1/Mash1 promotes brain oligodendrogenesis during myelination and remyelination. J. Neurosci. *33*, 9752–9768.

Nikbakht, H., Panditharatna, E., Mikael, L.G., Li, R., Gayden, T., Osmond, M., Ho, C.Y., Kambhampati, M., Hwang, E.I., Faury, D., et al. (2016). Spatial and temporal homogeneity of driver mutations in diffuse intrinsic pontine glioma. Nat. Commun. *7*, 11185.

Painter, J., and Merritt, E.A. (2006). Optimal description of a protein structure in terms of multiple groups undergoing TLS motion. Acta Crystallogr. D Biol. Crystallogr. *62*, 439–450.

Park, N.I., Guilhamon, P., Desai, K., McAdam, R.F., Langille, E., O'Connor, M., Lan, X., Whetstone, H., Coutinho, F.J., Vanner, R.J., et al. (2017). ASCL1 reorganizes chromatin to direct neuronal fate and suppress tumorigenicity of glioblastoma stem cells. Cell Stem Cell *21*, 209–224.e7.

Pathania, M., De Jay, N., Maestro, N., Harutyunyan, A.S., Nitarska, J., Pahlavan, P., Henderson, S., Mikael, L.G., Richard-Londt, A., Zhang, Y., et al. (2017). H3.3(K27M) cooperates with Trp53 loss and PDGFRA gain in mouse embryonic neural progenitor cells to induce invasive high-grade gliomas. Cancer Cell *32*, 684–700.e9.

Piunti, A., Hashizume, R., Morgan, M.A., Bartom, E.T., Horbinski, C.M., Marshall, S.A., Rendleman, E.J., Ma, Q., Takahashi, Y.H., Woodfin, A.R., et al. (2017). Therapeutic targeting of polycomb and BET bromodomain proteins in diffuse intrinsic pontine gliomas. Nat. Med. *23*, 493–500.

Schildge, S., Bohrer, C., Beck, K., and Schachtrup, C. (2013). Isolation and culture of mouse cortical astrocytes. J. Vis. Exp. https://doi.org/10.3791/50079.

Schmittgen, T.D., and Livak, K.J. (2008). Analyzing real-time PCR data by the comparative C(T) method. Nat. Protoc. *3*, 1101–1108.

Schwartzentruber, J., Korshunov, A., Liu, X.Y., Jones, D.T., Pfaff, E., Jacob, K., Sturm, D., Fontebasso, A.M., Quang, D.A., Tonjes, M., et al. (2012). Driver mutations in histone H3.3 and chromatin remodelling genes in paediatric glioblastoma. Nature *482*, 226–231.

Severino, M., Bertamino, M., Tortora, D., Morana, G., Uccella, S., Bocciardi, R., Ravazzolo, R., Rossi, A., and Di Rocco, M. (2016). Novel asymptomatic CNS findings in patients with ACVR1/ALK2 mutations causing fibrodysplasia ossificans progressiva. J. Med. Genet. *53*, 859–864.

Shah, N.M., Groves, A.K., and Anderson, D.J. (1996). Alternative neural crest cell fates are instructively promoted by TGFbeta superfamily members. Cell *85*, 331–343.

Silveira, A.B., Kasper, L.H., Fan, Y., Jin, H., Wu, G., Shaw, T.I., Zhu, X., Larson, J.D., Easton, J., Shao, Y., et al. (2019). H3.3 K27M depletion increases differentiation and extends latency of diffuse intrinsic pontine glioma growth in vivo. Acta Neuropathol. *137*, 637–655.

Swiss, V.A., Nguyen, T., Dugas, J., Ibrahim, A., Barres, B., Androulakis, I.P., and Casaccia, P. (2011). Identification of a gene regulatory network necessary for the initiation of oligodendrocyte differentiation. PLoS One *6*, e18088.

Tamaki, K., Souchelnytskyi, S., Itoh, S., Nakao, A., Sampath, K., Heldin, C.H., and ten Dijke, P. (1998). Intracellular signaling of osteogenic protein-1 through Smad5 activation. J. Cell. Physiol. *177*, 355–363.

Taylor, K.R., Mackay, A., Truffaux, N., Butterfield, Y.S., Morozova, O., Philippe, C., Castel, D., Grasso, C.S., Vinci, M., Carvalho, D., et al. (2014a). Recurrent activating ACVR1 mutations in diffuse intrinsic pontine glioma. Nat. Genet. *46*, 457–461.

Taylor, K.R., Vinci, M., Bullock, A.N., and Jones, C. (2014b). ACVR1 mutations in DIPG: lessons learned from FOP. Cancer Res. *74*, 4565–4570.

Tibes, R., Borad, M.J., Dutcus, C.E., Reyderman, L., Feit, K., Eisen, A., Verbel, D.A., and Von Hoff, D.D. (2018). Safety, pharmacokinetics, and preliminary efficacy of E6201 in patients with advanced solid tumours, including melanoma: results of a phase 1 study. Br. J. Cancer *118*, 1580–1585.

Tirosh, I., Venteicher, A.S., Hebert, C., Escalante, L.E., Patel, A.P., Yizhak, K., Fisher, J.M., Rodman, C., Mount, C., Filbin, M.G., et al. (2016). Single-cell RNA-seq supports a developmental hierarchy in human oligodendroglioma. Nature *539*, 309–313.

van Dinther, M., Visser, N., de Gorter, D.J., Doorn, J., Goumans, M.J., de Boer, J., and ten Dijke, P. (2010). ALK2 R206H mutation linked to fibrodysplasia ossificans progressiva confers constitutive activity to the BMP type I receptor and sensitizes mesenchymal cells to BMP-induced osteoblast differentiation and bone formation. J. Bone Miner. Res. *25*, 1208–1215.

Vinci, M., Burford, A., Molinari, V., Kessler, K., Popov, S., Clarke, M., Taylor, K.R., Pemberton, H.N., Lord, C.J., Gutteridge, A., et al. (2018). Functional diversity and cooperativity between subclonal populations of pediatric glioblastoma and diffuse intrinsic pontine glioma cells. Nat. Med. *24*, 1204–1215.

Walter, D.M., Venancio, O.S., Buza, E.L., Tobias, J.W., Deshpande, C., Gudiel, A.A., Kim-Kiselak, C., Cicchini, M., Yates, T.J., and Feldser, D.M. (2017). Systematic in vivo inactivation of chromatin-regulating enzymes identifies Setd2 as a potent tumor suppressor in lung adenocarcinoma. Cancer Res. *77*, 1719–1729.

Weinberg, D.N., Allis, C.D., and Lu, C. (2017). Oncogenic mechanisms of histone H3 mutations. Cold Spring Harb. Perspect. Med. *7*, https://doi.org/10.1101/cshperspect.a026443.

Winn, M.D., Ballard, C.C., Cowtan, K.D., Dodson, E.J., Emsley, P., Evans, P.R., Keegan, R.M., Krissinel, E.B., Leslie, A.G.W., McCoy, A., et al. (2011). Overview of the CCP4 suite and current developments. Acta Crystallogr. D Biol. Crystallogr. *67*, 235–242.

Wu, G., Broniscer, A., McEachron, T.A., Lu, C., Paugh, B.S., Becksfort, J., Qu, C., Ding, L., Huether, R., Parker, M., et al. (2012). Somatic histone H3 alterations in pediatric diffuse intrinsic pontine gliomas and non-brainstem glioblastomas. Nat. Genet. *44*, 251–253.

Wu, G., Diaz, A.K., Paugh, B.S., Rankin, S.L., Ju, B., Li, Y., Zhu, X., Qu, C., Chen, X., Zhang, J., et al. (2014). The genomic landscape of diffuse intrinsic pontine glioma and pediatric non-brainstem high-grade glioma. Nat. Genet. *46*, 444–450.

Yamamoto, M., Shook, N.A., Kanisicak, O., Yamamoto, S., Wosczyna, M.N., Camp, J.R., and Goldhamer, D.J. (2009). A multifunctional reporter mouse line for Cre- and FLP-dependent lineage analysis. Genesis *47*, 107–114.

STAR★METHODS

KEY RESOURCES TABLE

REAGENT or RESOURCE	SOURCE	IDENTIFIER
Antibodies		
p-SMAD1/5/8	Cell Signaling	13820; RRID:AB_2493181
p-SMAD1/5/8	Peter ten Dijke's laboratory	N/A
SMAD1	Cell Signaling	6944; RRID:AB_10858882
p-SMAD2	Cell Signaling	3108; RRID:AB_490941
SMAD2	Cell Signaling	3103; RRID:AB_490816
Id1	Santa Cruz	sc-133104; RRID:AB_2122863
Id2	Santa Cruz	sc-398104
Id3	Santa Cruz	sc-56712; RRID:AB_783921
p-ERK1/2	Cell Signaling	9101; RRID:AB_331646
ERK1/2	Cell Signaling	9102; RRID:AB_330744
H3-K27me3	Millipore	07-449; RRID:AB_310624
Histone H3	Abcam	ab10799; RRID:AB_470239
Rb	Cell Signaling	9309; RRID:AB_2297442
p-Rb	Cell Signaling	9308; RRID:AB_2141156
β-Actin	Sigma	A2066; RRID:AB_476693
α-Tubulin	Sigma	T6199; RRID:AB_477583
PDGFRA (mouse)	R&D	AF1062; RRID:AB_2236897
PDGFRA (human)	Cell Signaling	5241; RRID:AB_10692773
CNPase (CNP1)	Cell Signaling	D83E10; RRID:AB_10705455
Ki67	Abcam	ab156956; RRID:AB_2732028
GFAP	Dako	Z0334; RRID:AB_10013382
OLIG2	Millipore	MABN50; RRID:AB_10807410
Nestin	BD Pharmigen	556309; RRID:AB_396354
Biotinylated PDGFRA (mouse)	Thermo Fisher	13-1401-82; RRID:AB_466607
Anti-mouse-HRP	Amersham/GE Healthcare	NA9310; RRID:AB_772193
Anti-Rabbit-HRP	Amersham/GE Healthcare	NA934; RRID:AB_772206
Anti-rabbit-Alexa Fluor 680	Thermo Fisher	A21109; RRID:AB_2535758
Anti-mouse-IRDye 800CW	LI-COR	926-32210; RRID:AB_621842
Biotinylated rabbit anti-goat	Vector Laboratories	BA-5000; RRID:AB_2336126
Biotinylated rabbit anti-rat	Vector Laboratories	BA-4001; RRID:AB_10015300
Biotinylated goat anti-mouse	Vector Laboratories	BA-9200; RRID:AB_2336171
Biotinylated goat anti-rabbit	Vector Laboratories	BA-1000; RRID:AB_2313606
Cy5-conjugated donkey anti-rabbit	Jackson ImmunoResearch	711-175-152; RRID:AB_2340607
Streptavidin-FITC	BD Biosciences	554060; RRID:AB_10053373
Ki67-APC	Thermo Fisher	50-5698-82; RRID:AB_2574235
Bacterial and Virus Strains		
Bacterial strain for cloning: NEB® 5-alpha	New England BioLabs	C2987
Adenovirus: Ad-GFP	Vector Biolabs	1060
Adenovirus: Ad-GFP-Cre	Vector Biolabs	1700
Biological Samples		
Human DIPG tumor samples – frozen blocks	Dr. Michelle Monje's laboratory	N/A
Human DIPG tumor samples and normal brain tissue - RNAseq	Dr. Cynthia Hawkin's laboratory	N/A

(*Continued on next page*)

Continued		
REAGENT or RESOURCE	SOURCE	IDENTIFIER
Chemicals, Peptides, and Recombinant Proteins		
Neurobasal-A medium	Thermo Fisher	10888-022
D-MEM/F-12	Thermo Fisher	11330-032
HEPES solution for cell culture	Thermo Fisher	15630-080
Sodium pyruvate solution for tissue culture	Thermo Fisher	11360-070
MEM non-essential amino acids	Thermo Fisher	11140-050
GlutaMAX-I supplement	Thermo Fisher	35050-061
Antibiotic/antimycotic solution	Thermo Fisher	15240-096
TSM Base medium	Wisent	305-485-CL
Hanks' Balanced Salt solution	Wisent	311-510-CL
TrypLE Express Enzyme solution	Thermo Fisher	12604021
Trypsin solution	Wisent	325-542-EL
DMEM, high glucose, pyruvate	Thermo Fisher	11995-065
OPTI-MEM	Thermo Fisher	31985062
FuGENE HD	Promega	E2311
Fetal Bovine Serum	Wisent	098150
Fetal Bovine Serum	Thermo Fisher	26140079
Phosphate-buffered saline solution	Wisent	311-010
Heparin for tissue culture	StemCell Technologies	07980
Lenti-X Concentrator	Takara	631232
B-27 supplement without vitamin A	Thermo Fisher	12587-010
Recombinant H-EGF	Shenandoah Biotech	100-26
Recombinant H-FGF-basic-154	Shenandoah Biotech	100-146
Recombinant H-PDGF-AA	Shenandoah Biotech	100-16
Recombinant H-PDGF-BB	Shenandoah Biotech	100-18
Recombinant mouse noggin	Preprotech	250-38
Recombinant mouse follistatin	Shenandoah Biotech	200-24
Recombinant Mouse BMP-2	R&D	355-BM-010
Recombinant Mouse BMP-6	R&D	6325-BM-020
Recombinant Mouse BMP-9	R&D	5566-BP-010
Recombinant Mouse TGFβ	R&D	7666-MB-005
LDN-193189	Sigma-Aldrich	SML0559
Trametinib	MedKoo Biosciences	201458
Buparlisib	MedKoo Biosciences	204690
E6201	Spirita Oncology	N/A
Complete, Mini, EDTA-free Protease Inhibitor Cocktail	Roche	11836170001
PhosSTOP phosphatase inhibitor tablets	Roche	4906845001
4X Bolt LDS Sample Buffer	Thermo Fisher	B0007
10X Bolt Sample Reducing Agent	Thermo Fisher	B0009
Bolt 4-12% Bis-Tris Plus gels	Thermo Fisher	NW04120BOX
BioTrace NT nitrocellulose membrane for western blot	PALL Life Sciences	66485
PVDF membrane for western blot	Roche	03010040001
Immobilon PVDF membrane for western blot	Millipore	IPFL00010
20X Bolt MES SDS Running Buffer	Thermo Fisher	B0002
20X Bolt Transfer Buffer	Thermo Fisher	BT0006
Luminata Crescendo Western HRP Substrate	Millipore	WBLUR0100
Odyssey Blocking Buffer	LI-COR	927-50000
2X Power SYBR Green PCR Sample Mix	Thermo Fisher	4368708
Tracer-6908	Promega	https://zenodo.org/record/1308267#.Xd1vja8VixA

(*Continued on next page*)

Continued

REAGENT or RESOURCE	SOURCE	IDENTIFIER
Intracellular TE Nano-Glo Substrate/Inhibitor	Promega	N2160
Lipofectamine 3000 Transfection Reagent	Thermo Fisher	L3000015
DharmaFECT Duo	Thermo Fisher	T-2010-03
Optimal Cutting Temperature (O.C.T.) compound	VWR	25608-930
Entellan	Sigma Aldrich	1079600500
Vectashield Antifade Mounting Medium	Vector Laboratories	H-1000
TBS LC-A /Mount Low Viscosity Mounting Medium	Cole-Parmer	RK-48579-05
Dynabeads A	Thermo Fisher	10002D
Dynabeads G	Thermo Fisher	10003D
Cytofix/Cytoperm Buffer	BD Biosciences	554722
Perm/Wash Buffer	BD Biosciences	554723
AnnexinV-FITC	BioLegend	640906
7-AAD	BioLegend	420404
Critical Commercial Assays		
NucleoSpin Tissue Kit	Macherey-Nagel	740952
NucleoSpin Plasmid Kit	Macherey-Nagel	740588
NucleoSpin RNA Plus kit	Macherey-Nagel	740984
NucleoSpin TriPrep kit	Macherey-Nagel	740966
MinElute PCR purification kit	Qiagen	28004
TOPO-TA cloning kit	Thermo Fisher	451641
iScript cDNA synthesis kit	Bio-Rad	1708891
TruSeq Stranded Total RNA kit	Illumina	20020596
PrestoBlue Cell Viability Reagent	Thermo Fisher	A13261
ATPlite Luminescence Assay System	Perkin Elmer	6016947
Dual-Luciferase Reporter Assay System	Promega	E1910
VECTASTAIN Elite ABC HRP Kit	Vector Laboratories	PK-6100
Click-iT EdU Alexa Fluor 647 Flow Cytometry kit	Thermo Fisher	C10424
Deposited Data		
RNA sequencing data, $Acvr1^{+/+}$;$Olig2^{Cre/+}$ and $Acvr1^{floxG328V/+}$;$Olig2^{Cre/+}$ postnatal day 7 brainstems	This paper	GEO: GSE142776
X-ray crystal structure of the ACVR1-FKBP12 complex bound to E6201	This paper	PDB: 6I1S
Experimental Models: Cell Lines		
SU-DIPG-IV	Dr. Michelle Monje's laboratory	RRID: CVCL_IT39
SU-DIPG-VI	Dr. Michelle Monje's laboratory	RRID:CVCL_IT40
SU-DIPG-XXI	Dr. Michelle Monje's laboratory	N/A
SU-DIPG-XXXVI	Dr. Michelle Monje's laboratory	N/A
HSJD-DIPG-007	Dr. Ángel Montero Carcaboso's laboratory	RRID:CVCL_VU70
HEK293T	ATCC	RRID:CVCL_0063
C2C12	ATCC	RRID:CVCL_0188
293FT	Thermo Fisher	R70007
Experimental Models: Organisms/Strains		
Mouse: $Acvr1^{floxG328V}$	This paper	N/A
Mouse: $Hist1h3b^{K27M}$	This paper	N/A
Mouse: $Pik3ca^{floxH1047R}$ (FVB.129S6-*Gt(ROSA)* $26Sor^{tm1(Pik3ca*H1047R)Egan}$/J)	The Jackson Laboratory	016977; RRID:IMSR_JAX:016977
Mouse: $Olig2^{Cre}$ (B6.129-$Olig2^{tm1.1(cre)Wdr}$/J)	The Jackson Laboratory	025567; RRID:MGI:4844109
Mouse: B6.FVB-Tg(EIIa-cre)C5379Lmgd/J	The Jackson Laboratory	003724; RRID:IMSR_JAX:003724

(*Continued on next page*)

Continued

REAGENT or RESOURCE	SOURCE	IDENTIFIER
Mouse: *ROSA26*$^{LSL\text{-}tdTomato}$ (B6;129S6-*Gt(ROSA)26Sor*$^{tm9(CAG\text{-}tdTomato)Hze}$/J)	The Jackson Laboratory	007905; RRID:IMSR_JAX:007905
Mouse: *Acvr1*^{tnR206H}	Lees-Shepard et al., 2018	N/A
Mouse: *Pdgfra-Cre* (C57BL/6-Tg(Pdgfra-cre)1Clc/J)	The Jackson Laboratory	013148; RRID:IMSR_JAX:013148
Mouse: *R26*^{NG} (*Gt(ROSA)26Sor*$^{tm1.2(CAG\text{-}EGFP)Glh}$)	Yamamoto et al., 2009	RRID:IMSR_JAX:012429
Mouse : NSG NOD.Cg-*Prkdc*^{scid} *Il2rg*^{tm1Wjl}/SzJ)	The Jackson Laboratory	005557; RRID:IMSR_JAX:005557
Oligonucleotides		
Oligonucleotides for cloning	This paper	See Table S4
Oligonucleotides for quantitative PCR	This paper	See Table S4
Oligonucleotides for sgRNA cloning	This paper	See Table S4
Recombinant DNA		
pKOII	Bardeesy et al., 2002	N/A
LentiCRISPRv2GFP	Addgene; Laboratory of Dr. David Feldser	82416
psPAX2	Addgene; Laboratory of Dr. Didier Trono	2260
pMD2.G	Addgene; Laboratory of Dr. Didier Trono	12259
ca-ACVR1 (human - Q207D)	Laboratory of Dr. Kohei Miyazono	N/A
ca-BMPR1A (human - Q233D)	Laboratory of Dr. Kohei Miyazono	N/A
ca-BMPR1B (mouse - Q203D)	Laboratory of Dr. Kohei Miyazono	N/A
pLEX306	Addgene; Laboratory of Dr. David Root	41391
pLEX306-iCre	Laboratory of Dr. Daniel Schramek	N/A
pLEX306-tdTomato-iCre	Laboratory of Dr. Daniel Schramek	N/A
pLEX306-SOX11-iCre	Laboratory of Dr. Daniel Schramek	N/A
pLEX306-ASCL1-iCre	This paper	N/A
NanoLuc Protein Fusion MCS Vector	Promega	N1361
Renilla Luciferase Control Reporter Vector	Promega	E2261
6XE2F-Luciferase	Laboratory of Dr. Kristian Helin	N/A
BRE-Luciferase	Laboratory of Dr. Peter ten Dijke	N/A
CAGA-Luciferase	Laboratory of Dr. Petra Knaus	N/A
Software and Algorithms		
Image J	National Institutes of Health	https://imagej.nih.gov/ij/
Image Studio Ver.5.0	Li-COR	N/A
FASTQC v0.11.5	Babraham Bioinformatics	https://www.bioinformatics.babraham.ac.uk/projects/fastqc/
MultiQC v0.8	Ewels et al., 2016	N/A
STAR aligner v2.5.2b	Dobin et al., 2013	N/A
RSEM v1.3.0	Li and Dewey, 2011	N/A
DESeq2 R package v1.20.0	Love et al., 2014	N/A
Trimmomatic v0.35	Bolger et al., 2014	N/A
GenePattern	Broad Institute	https://software.broadinstitute.org/cancer/software/genepattern/
SynergyFinder	Ianevski et al., 2017	https://synergyfinder.fimm.fi

(*Continued on next page*)

Continued		
REAGENT or RESOURCE	SOURCE	IDENTIFIER
MOSFLM	Leslie, 2006	N/A
AIMLESS (CCP4 suite)	Winn et al., 2011	N/A
PHASER	McCoy et al., 2007	N/A
Phenix Refine	Adams et al., 2010	N/A
COOT	Emsley and Cowtan 2004	N/A
TLSMD	Painter and Merritt 2006	N/A
MolProbity	Davis et al., 2007	N/A
ZEN pro imaging software	Zeiss	N/A
NDP.view2 imaging software	Hamamatsu	N/A
FlowJo version 10	Becton, Dickinson & Company	N/A
Prism version 7.03	GraphPad	N/A

LEAD CONTACT AND MATERIALS AVAILABILITY

Further information and requests for resources and reagents should be directed to and will be fulfilled by the Lead Contact, Tak Mak (tmak@uhnresearch.ca). All unique/stable reagents generated in this study are available from the Lead Contact with a completed Materials Transfer Agreement.

EXPERIMENTAL MODEL AND SUBJECT DETAILS

In Vivo Animal Studies

For mouse studies, male and female animals were used. Depending on the experiment, mice were analyzed at specific postnatal days or in adulthood, as indicated in the text and in figure legends. Mice were on a mixed genetic background, and housed in temperature-controlled facilities under 12-hour light/12-hour dark conditions with access to food and water *ad libitum*. Littermates carrying appropriate genotype(s) were randomly assigned to experimental groups. All animal experiments were performed in accordance with institutional and federal guidelines, and approved by Animal Care Committees (Princess Margaret Cancer Centre: protocol #985; Toronto Centre for Phenogenomics: protocol #22-0272H).

Human Studies

The RNA sequencing data from human tumor samples and normal brain tissue used in this paper were generated as part of a study being conducted at the Hospital for Sick Children (Toronto, Canada). Patients provided informed consent, and ethical approval was obtained from the Hospital for Sick Children Research Ethics Board (#1000055059).

Cell Lines and Primary Cultures

All cell lines and primary cultures were maintained in humidified cell culture incubators at 37°C under 5% CO_2. HEK293T and C2C12 cells were obtained from ATCC. 293FT cells were purchased from Thermo Fisher. For mouse primary cell cultures, female and male littermates were used. Specific culture media varied depending on the cell lines or primary cell type, as described in the "Method Details" section. SU-DIPG-IV, SU-DIPG-VI, SU-DIPG-XXI, and SU-DIPG-XXXVI were a gift from Dr Michelle Monje (Stanford University, CA, USA). HSJD-DIPG-007 cells were a gift from Dr Ángel Montero Carcaboso (Hospital Sant Joan de Déu, Barcelona, Spain). Authentication of the DIPG cell lines was performed by Short Tandem Repeat profiling at The Centre for Applied Genomics (SickKids, Toronto, Canada).

Studies Using Organisms as Source for Materials Used in Experiments

For X-ray crystallography studies, the ACVR1 kinase domain (residues 172–499) proteins were prepared from Sf9 insect cells, and the FKBP12 proteins were prepared from *E coli* strain BL21(DE3)R3-pRARE2.

METHOD DETAILS

Mice

Acvr1^{floxG328V} Allele

For the *Acvr1^{floxG328V}* allele, a targeting vector comprising the following elements was constructed: 1) an upstream homology arm comprising 3 kb of intron 7 of the *Acvr1* gene, amplified by PCR as an *HpaI-KpnI* fragment; 2) a *loxP*-flanked cassette comprising a minigene encoding exons 7-11 and the 3' untranslated region of *Acvr1* amplified by PCR as a *KnpI-NheI* fragment, as well as a

NheI-SalI flanked transcriptional stop cassette comprising three copies of the SV40 polyadenylation sequence; 3) an *Frt*-flanked neomycin resistance cassette; 4) a downstream homology arm comprising 6 kb of the *Acvr1* gene, including exon 8, all of intron 8, exon 9, and a portion of intron 9, amplified by PCR as a *XmaI-NotI* fragment. A missense mutation converting glycine 328 to valine in exon 8 was introduced by site-directed mutagenesis. The targeting vector was assembled in the pKOII backbone (Bardeesy et al., 2002), downstream of a negative selection cassette encoding the diphtheria toxin A chain. The targeting vector was verified by sequencing (ACGT Corporation, Toronto, Canada) and linearized with *NotI* prior to electroporation in E14K embryonic stem cells.

Hist1h3b^{K27M} Allele

In mice, *Hist1h3b*, the orthologous gene to human *HIST1H3B*, is located within a histone gene cluster whose organization is perfectly conserved with humans (Marzluff et al., 2002), although in mice it encodes H3.2, which differs from H3.1 by a single amino acid and can also be mutated in DIPGs. A targeting vector comprising the following elements was constructed: 1) an upstream homology arm comprising 4 kb of sequence upstream of the promoter of the single-exon *Hist1h3b* gene, amplified by PCR as a *KpnI-XhoI* fragment; 2) an *Frt*-flanked neomycin resistance cassette; 3) a downstream homology arm comprising 2.3 kb including the *Hist1h3b* gene promoter, coding sequence, and 3' region, amplified by PCR as a *BamHI-NotI* fragment. A missense mutation converting lysine 27 (proper nomenclature is in fact lysine 28) to methionine was introduced by site-directed mutagenesis. The targeting vector was assembled in the pKOII backbone(Bardeesy et al., 2002), downstream of a negative selection cassette encoding the diphtheria toxin A chain. The targeting vector was verified by sequencing (ACGT Corporation, Toronto, Canada) and linearized with *NotI* prior to electroporation in E14K embryonic stem cells.

For both alleles, 125 μg of linearized targeting vector were electroporated in 30 million E14K ES cells. The electroporated cells were plated in a total of ten 10-cm cell culture dishes coated with 1% gelatin, and cultured in ES cell medium supplemented with 0.325 mg/mL G418. After 9 days of selection, 480 clones were picked and amplified in 96-wells plates. Proper targeting was verified by long-range PCR (Terra Taq, Clontech) on genomic DNA extracted from the ES cells, using a combination of primers in the selection cassette and outside of the homology arms. Presence of the *loxP* sites and point mutations was assessed by sequencing of the PCR products. Correctly targeted ES cells were microinjected in C57Bl/6 blastocysts, and transferred into the uterine horns of pseudopregnant females. Highly chimeric mice were backcrossed to C57Bl/6 mice, and germline transmission of the mutant alleles was tested by PCR screening of brown pups. Excision of the neomycin resistance cassette by breeding heterozygous *Acvr1*^{floxG328V} animals to "flp deleter" mice (*B6.129S4-Gt(ROSA)26Sor*$^{tm1(FLP1)Dym/RainJ}$; Jax #009086) resulted in sporadic leaky activation of the mutation, compromising the viability of a substantial proportion of the animals. Therefore, experiments were performed using mice that retained the selection cassette, which did not interfere with expression of the mutant *Acvr1* allele.

Acvr1$^{tnR206H/+}$*;Pdgfra-Cre* mice (Lees-Shepard et al., 2018) and the *R26*^{NG} *Cre*-dependent GFP reporter allele (Yamamoto et al., 2009) have been described previously. *Olig2*^{Cre} (B6.129-Olig2$^{tm1.1(cre)Wdr}$/J, Jax #025567), *EIIa-Cre* (B6.FVB-Tg(EIIa-cre) C5379Lmgd/J, Jax #003724), *Nestin-Cre* (B6.Cg-Tg(Nes-cre)1Kln/J, Jax #003771), *Pik3ca*^{floxH1047R} (Gt(ROSA)26Sor$^{tm1\ (Pik3ca*H1047R)Egan}$, Jax #016977) and *ROSA26*$^{LSL-tdTomato}$ (B6.Cg-Gt(ROSA)26Sor$^{tm14(CAG-tdTomato)Hze}$/J; Jax #007914) mice were obtained from The Jackson Laboratory. NSG (NOD.Cg-*Prkdc*^{scid} *Il2rg*^{tm1Wjl}/SzJ) mice were obtained from in-house breeding colonies at the Toronto Center for Phenogenomics.

Patient-Derived DIPG Cell Lines

SU-DIPG-IV, SU-DIPG-VI, SU-DIPG-XXI, and SU-DIPG-XXXVI were obtained from the laboratory of Dr Michelle Monje (Stanford University, CA, USA). HSJD-DIPG-007 cells were a generous gift from Dr Ángel Montero Carcaboso (Hospital Sant Joan de Déu, Barcelona, Spain). Cells were cultured as described (Grasso et al., 2015). Cells were maintained in Tumor Stem Medium (TSM) (1:1 mixture of Neurobasal-A medium (Thermo Fisher #10888-022) and D-MEM/F-12 (Thermo Fisher #11330-032) supplemented with 10 mM HEPES (Thermo Fisher #15630-080), 1 mM sodium pyruvate (Thermo Fisher #11360-070), 0.1 mM MEM non-essential amino acids (Thermo Fisher #11140-050), 1X GlutaMAX-I supplement (Thermo Fisher #35050-061), 1X antibiotic/antimycotic (Thermo Fisher #15240-096), 1X B-27 supplement without vitamin A (Thermo Fisher #12587-010), 20 ng/mL recombinant H-EGF (Shenandoah Biotech #100-26), 20 ng/mL recombinant H-FGF-basic-154 (Shenandoah Biotech #100-146), 10 ng/mL recombinant H-PDGF-AA (Shenandoah Biotech #100-16), 10 ng/mL recombinant H-PDGF-BB (Shenandoah Biotech #100-18), and 2 ug/mL heparin (StemCell Technologies #07980). TSM medium, containing the above components without B-27 and recombinant ligands, was also custom-formulated by Wisent (#305-485-CL). For routine culture, cells were maintained in a humidified cell culture incubator at 37°C under 5% CO_2. Cells were passaged and medium was changed every 7 days. Typically, 0.2 x 10^6 SU-DIPG-IV cells, 2 x 10^6 SU-DIPG-VI cells, 1 x 10^6 SU-DIPG-XXI, 0.2 x 10^6 SU-DIPG-XXXVI, and 0.4 x 10^6 HSJD-DIPG-007 cells were plated in T-75 flasks to achieve confluence after 7 days of culture.

The DIPG cell lines were authenticated by Short Tandem Repeat profiling at The Centre for Applied Genomics (SickKids, Toronto, Canada). Furthermore, the presence and expression of the *ACVR1* mutations were confirmed by PCR amplification and sequencing of *ACVR1* coding regions from genomic DNA, and of the whole *ACVR1* coding sequence from cDNA prepared from the cell lines, using the primers listed in Table S4.

CRISPR/Cas9 Gene Editing in Patient-Derived DIPG Cells Lines

sgRNA-encoding oligonucleotides were designed using ZiFiT and CRISPR Design (MIT) and synthesized by Eurofins Genomics. Oligos were phosphorylated by incubating 100 nmol of the sense and antisense oligonucleotides in a 20 μl reaction mixture containing 1 mM ATP, 1X T4 reaction buffer, and 10 units T4 PNK (Promega) for 1 hour at 37°C. Oligos were then annealed by adding 70 μl H_2O

and 10 μl of 10X annealing buffer (100 mM Tris-HCL pH7.5, 10 mM EDTA, 500 mM NaCl, 20 mM $MgCl_2$), and incubating at 95°C for 5 minutes, 85°C for 4 minutes, and ramping down the temperature by 0.5°C per minute from 80°C to 10°C in a PCR thermocycler. The annealed oligos were then cloned into the *Bsmb1* site of the LentiCRISPRv2GFP lentiviral vector (Walter et al., 2017). Oligonucleotide sequences are provided in Table S4.

For lentivirus production, 3 x 10^6 HEK293FT cells were transfected with 8 μg lentiviral plasmid, 6 μg psPAX2 packaging plasmid, and 2 μg pMD2.G envelope plasmid (laboratory of Didier Trono) using Lipofectamine 3000 in 10-cm dishes. The next day, growth medium was replaced with 10mL fresh growth medium containing 20% FBS. 24 hours later, the medium was collected, spun at 300 x g to remove cells and debris and passed through a 22 μm MCE membrane syringe filter (Millipore). Lentiviral particles were precipitated by adding 1:3 parts Lenti-X Concentrator (Takara) and incubating overnight at 4°C. Precipitated viral particles were pelleted by centrifugation at 1500 x g for 45 minutes, and the pellet resuspended in 300 μl PBS. Viral particles were immediately used, or stored at -80°C.

For infection of DIPG cell lines, 1.5 x 10^5 cells were transduced by mixing the appropriate lentiviral particles with 2 μg/mL polybrene in TSM culture medium, and incubated in a humidified cell culture incubator at 37°C under 5% CO_2. In all experiments, a pool of two lentiviruses encoding distinct sgRNAs was used, to maximize target disruption in the non-clonal edited cell population. The amount of viral particles was balanced across conditions. The next day, cells were pelleted by 300 x g centrifugation, washed once in 1X HBSS, resuspended in TSM culture medium, and returned to the cell culture incubator until analysis.

To verify target editing, genomic DNA was extracted from GFP-positive sorted cells using the NucleoSpin Tissue kit (Macherey-Nagel #740952), according to the manufacturer's instructions. The genomic regions comprising the targeted loci were amplified by PCR using primers listed in Table S4, and cloned into the pCR2.1-TOPO vector using the TOPO-TA cloning kit (Thermo Fisher). Ligation products were transformed into DH5α bacteria (New England BioLabs) and plated on ampicillin-coated agar plates. Plasmid DNA was extracted from randomly selected bacterial colonies using the NucleoSpin Plasmid kit (Macherey-Nagel #740588), and sequenced (ACGT Corporation, Toronto, Canada).

Neural Stem Cell Culture and Derivation of Mouse Tumor Cell Lines

To derive tumor cell lines and normal neural stem cell cultures, the thalamic, midbrain and rostral hindbrain regions from postnatal day 2 pups (for normal neural stem cells), or brain tumors from mice at humane endpoint (for tumor cell lines), were dissected, cut in small pieces, and incubated at 37°C in 5 mL TrypLE reagent (Thermo Fisher) for 10 minutes. The tissue was dissociated by repeated pipetting, washed with 20 mL 1X HBSS, and filtered through a 100 μM nylon mesh. Cells were centrifuged at 300 x g for 5 minutes, resuspended in 2 mL TSM growth medium (described above), and plated in one well of a 6-wells cell culture plate. Cells were maintained in a humidified cell culture incubator at 37°C under 5% CO_2, typically reaching confluence after 5-7 days. At that point, cells were routinely passaged and expanded as described for DIPG cell lines. All experiments with normal neural stem cells were performed on cells between the first and fourth passage.

Lentivirus Transduction of Neural Stem Cells, and Neurosphere-Forming Assays

For ectopic expression experiments, the pLEX306 vector, a gift from David Root (Addgene plasmid # 41391) was modified to encode Cre recombinase instead of a puromycin resistance cassette, by conventional cloning using *KpnI* and *HpaI*, generating pLEX306-iCre. The coding sequences for tdTomato or mouse SOX11 were inserted in-frame with a C-terminal V5 tag into pLEX306-iCre using gateway cloning. For ASCL1, the full-length coding sequence of the mouse *Ascl1* gene was amplified from cDNA generated from total brain RNA, using the primers listed in Table S4, and cloned between the *NheI* and *EcoRV* restriction sites of pLEX306-iCre. Lentiviral particles were generated, and neural stem cells were processed for lentiviral transduction, as described above for DIPG cell lines.

For neurosphere-forming assays, 0, 10, 20, 100, 200 or 1000 cells were plated in individual wells in ultra-low attachment polystyrene 96-wells plates (Corning) (6 replicate wells per condition). The number of neurospheres in each well was counted 7 days after plating.

Primary Brainstem Glial Cell Culture and Adenovirus Infection

Primary brainstem glial cells were prepared by adapting previously published procedure developed for cortical cultures (Schildge et al., 2013). Brainstems were dissected from postnatal day 3 pups under a Leica MZ75 dissection microscope in cold 1X HBSS. The brainstem tissue was chopped in small pieces, and transferred to a 50-mL conical tube containing 1X HBSS supplemented with 0.05% Trypsin. The tissue was incubated in a 37°C water bath for 30 minutes, with occasional vortexing, and centrifuged at 300 x g. The liquid was aspirated, and the tissue resuspended in 10mL warm growth medium (DMEM supplemented with 1X antibiotic/antimycotic and 10% heat-inactivated fetal bovine serum). A single-cell suspension was prepared by pipetting up-and-down several times, followed by passing through a 100 μm nylon mesh. Additional growth medium was added to a final volume of 20 mL, and the cell suspension plated in a T-75 flask coated with 50ug/mL poly-D-lysine. Cells were maintained in a humidified cell culture incubator at 37°C under 5% CO_2. 9 days after plating (DIV9), confluent cells were split for experiments. Medium was aspirated, cells were washed with 1X phosphate-buffered saline (PBS), and dissociated in 0.05% trypsin. For adenovirus infection, 0.75 x 10^6 cells were plated in 10-cm dishes. The next day, 20 x 10^6 PFU of the appropriate adenoviruses (Ad-GFP; Vector Biolabs #1060, or Ad-GFP-Cre, Vector Biolabs #1700) were added to the plates in 8mL growth medium. 24 hours later, the viral transduction medium was replaced with 8 mL fresh growth medium. The following day, cells were washed with PBS and incubated with serum-free medium supplemented with drugs or recombinant ligands as indicated. For gene expression and protein analyses, cells were collected in trypsin 20 hours later.

C2C12 and HEK-293 Cells Culture

C2C12 and KEK-293 cells were maintained in DMEM medium (Gibco, Thermo Fisher) supplemented with 10% fetal bovine serum (FBS) (Thermo Fisher), and penicillin/ streptomycin (Thermo Fisher).

Drugs

E6201 was obtained from Spirita Oncology. LDN-193189 was purchased from Sigma-Aldrich. Trametinib and Buparlisib were purchased from MedKoo Biosciences. Recombinant mouse noggin was from Preprotech, and mouse follistatin was from Shenandoah Biotechnology. For *in vivo* administration, E6201 was supplied by Spirita Oncology in lyophilized form, pre-weighted in sealed vials, and reconstituted with sterile water for injection, yielding a final concentration of 6 mg/mL in 30% Captisol. Vehicle control solution was prepared by dissolving Captisol in sterile water for injection. The drug was dissolved freshly before each injection. The drug was administered by intraperitoneal injections, using 27 Gauge needles fitted to 0.5 mL syringes.

Xenograft Models

DIPG tumor xenografts were performed following previously-described guidelines (Grasso et al., 2015). 2×10^5 cells, in a volume of 2 μL phosphate-buffered saline, were injected in the brainstem (3 mm posterior to lambda suture; 3 mm deep) of cold-anesthetized, postnatal day 2 NSG mice, using a 27 Gauge Hamilton syringe fitted to a custom stereotactic apparatus. For xenograft of mouse-derived cell lines, injections were targeted to the midbrain (thalamus) or hindbrain (brainstem) regions.

RNA and Protein Extraction

For cultured cells, RNA was extracted using the NucleoSpin RNA Plus kit (Macherey-Nagel #740984). In some experiments, DNA, RNA and proteins were extracted using the NucleoSpin TriPrep kit (Macherey-Nagel #740966), following the manufacturer's instructions. For RNA extraction from tissues, samples were first homogenized in LPB buffer from the NucleoSpin RNA Plus kit using a Buller Blender Gold apparatus (Next Advance) and 0.5 mm zirconium oxide beads. For protein extraction from tissues, samples were sonicated in RIPA buffer (10 mM Tris-HCL pH8.0, 1 mM EDTA, 1% Triton X-100, 0.1% sodium deoxycholate, 0.1% SDS, 140 mM NaCl, supplemented with protease and phosphatase inhibitors (Complete Mini and PhosSTOP, Roche)) in 1.5 mL microtubes on ice with five 5-sec pulses at power3 on a Misonix XL-2000 instrument, with a 10-sec timeout on ice between each pulse. The lysates were further incubated with rotation for 30 minutes at 4°C, spun at 11000 x g at 4°C for 15 minutes, and the supernatants transferred to new 1.5 mL microtubes. RNA and protein samples were stored at -80°C until further analysis.

Western Blotting

For western blotting, proteins samples were prepared by mixing equal amounts of proteins from cell or tissue lysates with 1X Bolt LDS Sample Buffer (Thermo Fisher #B0007), 1X Bolt Sample Reducing Agent (Thermo Fisher #B0009), and incubating at 70°C for 10 minutes with constant motion. The volume of all samples was equilibrated with lysis buffer. Samples were separated on Bolt 4-12% Bis-Tris Plus gels (Thermo Fisher), following the manufacturer's instructions. Proteins were transferred on nitrocellulose (BioTrace NT, PALL Life Sciences) or PVDF (#03010040001, Roche) membranes in Bolt Transfer Buffer for 1 hour at 30 volts at room temperature. Membranes were blocked with TBST buffer (50 mM Tris-HCl pH7.5, 150 mM NaCl, 0.1% Tween-20) containing 5% powdered milk, or 5% bovine serum albumin (BSA), for 1 hour at room temperature. Membranes were then incubated overnight in primary antibody solution, washed three times in TBST, incubated for 1 hour in TBST containing 5% powdered milk or 5% BSA and secondary antibodies and washed three times in TBST. Chemiluminescent detection was performed using Luminata Crescendo Western HRP Substrate (Millipore #WBLUR0100) and exposing to film (HyBlot CL, Denville Scientific). In some experiments, the western blot membranes were processed with the LI-COR system (LI-COR biotechnology), in which case blocking was performed using the Odyssey Blocking Buffer, and antibody incubations were performed in blocking buffer supplemented with 0.1% Tween-20. Fluorescence detection was performed with an Odyssey CLx instrument (LI-COR). Primary antibodies used were: p-SMAD1/5/8 (Cell Signaling #13820; 1:1000), p-SMAD1/5/8 (laboratory of Peter ten Dijke, 1:1000 (Tamaki et al., 1998)), SMAD1 (Cell Signaling #6944, 1:1000), p-SMAD2 (Cell Signaling #3108, 1:1000), SMAD2 (Cell Signaling #3103, 1:1000), Id1 (Santa Cruz #sc-133104, 1:1000), Id2 (Santa Cruz #sc-398104, 1:1000), Id3 (Santa Cruz #sc-56712), p-ERK1/2 (Cell Signaling #9101, 1:1000), ERK1/2 (Cell Signaling #9102, 1:1000), H3-K27me3 (Millipore #07-449, 1:1000), total H3 (Abcam #ab10799, 1:3000), Rb (Cell Signaling #9309, 1:1000), p-Rb (Cell Signaling #9308, 1:1000) , β-Actin (Sigma #A2066, 1:5000), α-Tubulin (Sigma #T6199, 1:5000). Secondary antibodies used were: mouse-HRP (Amersham #NA9310, 1:5000), Rabbit-HRP (Amersham #NA934, 1:5000), rabbit-Alexa Fluor 680 (Thermo Fisher #A21109, 1:5000), mouse-IRDye 800CW (LI-COR #926-32210, 1:10000). Densitometry analyses were performed using Image J.

cDNA Synthesis and Quantitative PCR

cDNA was synthesized using the iScript kit (Bio-Rad), following the manufacturer's instructions. For quantitative PCR, 10 μl reactions comprising 5 μl 2X Power SYBR Green PCR Sample Mix (Applied Biosystems), 3 μl H_2O, 0.5 μl of each primer (at a 10 μM concentration), and 1 μl cDNA were assembled. Samples were run on a ABI 7900HT Fast Real-Time PCR system (Applied Biosystems) with the following parameters: 95°C for 10 minutes, followed by 45 cycles of 95°C for 15 seconds and 60°C for 1 minute. In all experiments, expression of the genes of interest was normalized to the expression of the housekeeping gene *Rpl19* for mouse samples, and *RPL19* for human samples. Relative gene expression was calculated with the comparative C_t method (Schmittgen and Livak, 2008). All the primers are listed in Table S4.

RNA-Sequencing and Analysis

For RNA-sequencing on mouse brainstem samples, total brainstem RNA was processed, and library preparation was performed using the TruSeq Stranded Total RNA kit (Illumina), following the manufacturer's instructions. Sequencing was performed on a Nextseq 500 instrument (Illumina), using a 75-cycle paired-end read protocol and multiplexing, to obtain approximately 40 million reads per sample. Library preparation and sequencing were performed at the Princess Margaret Genomics Centre (Toronto, Canada). The raw 75-basepair paired-end reads from the sequencer were first quality-checked using FASTQC v0.11.5 (https://www.bioinformatics.babraham.ac.uk/projects/fastqc/) and MultiQC v0.8 (Ewels et al., 2016) software packages, and then aligned to the *Mus musculus* genome assembly version GRCm38 (mm10) from the Genome Reference Consortium using the STAR aligner v2.5.2b (Dobin et al., 2013). The aligned transcripts were quantified using RSEM v1.3.0 (Li and Dewey, 2011). The RSEM quantification output files were then further processed using in-house R scripts to create a gene-by-sample expression matrix. This matrix of raw RSEM expected counts was then input into the DESeq2 R package v1.20.0 (Love et al., 2014) for differential expression analysis. Within DESeq2, the default procedure recommended by the package vignette was generally followed. Since the samples were processed and sequenced in two batches, the batch information was included as a feature variable in the linear modeling design formula to account for any batch effects. For statistical analyses, only genes with FPKM>10 were considered. The baseMean (mean of normalized counts across samples), $\log_2$FoldChange, and the adjusted p-values obtained using the Benjamini-Hochberg procedure, were used to arrive at the set of top differentially expressed genes. Heatmaps were produced using the pheatmap R package.

Gene Set Enrichment Analysis (GSEA) was performed using the GenePattern platform (Broad Institute, MIT). The "GSEAPreranked" module was used on ranked lists of differentially expressed genes. Gene sets queried were: the "Canonical Pathways" and "Chemical and Genetic Perturbations" subsets of C2 (Curated Gene Sets), C5 (Gene Ontology Gene Sets), and C6 (Oncogenic Signatures).

For RNA sequencing analyses on human tumor samples and normal brain tissue, patients provided informed consent, and ethical approval was obtained from the Hospital for Sick Children Research Ethics Board (#1000055059). Total RNA was extracted from fresh-frozen tissue samples using the RNeasy mini kit (Qiagen) using the manufacturer's guidelines. Sample quality was confirmed using Bioanalyzer 2100 (Agilent). 34 DIPG and 17 normal brain samples passed quality control. Paired-end, stranded libraries were constructed using TruSeq Stranded Total RNA Library Prep with Ribo-Zero Gold Kit (Illumina) and sequenced on Illumina HiSeq 2500 instruments. After confirming sequencing quality with FastQC v0.11 (http://www.bioinformatics.babraham.ac.uk/projects/fastqc/), reads were quality trimmed with Trimmomatic v0.35 and aligned to human transcriptome build GRCh37 v75 using RSEM v1.2. Gene expression was quantified as transcripts-per-million. Further information regarding these samples are available from the laboratory of Dr Cynthia Hawkins (Hospital for Sick Children, Toronto, Canada), and the full data set will be published elsewhere.

Cell Growth and Viability Assays

For primary glial cell culture proliferation assays, 5000 cells in 200 μl growth medium were seeded in six wells of four 96-wells plates for each genotype or condition. At the indicated time points after plating, 10 μl PrestoBlue reagent (Thermo Fisher) was added to each well, and the cells placed back in a cell culture incubator. Four hours after PrestoBlue addition, absorbance was measured on a FlexStation 3 plate reader (Molecular Devices) under fluorescence settings with an excitation wavelength of 560 nm and an emission wavelength of 590 nm.

For patient-derived DIPG cell lines growth and viability assays, cells were seeded in TSM medium in a volume of 80 μl/well in 96-well plates (SU-DIP-IV: 4000 cells/well; SU-DIPG-VI: 10 000 cells/well; SU-DIPG-XXI: 8000 cells/well; SU-DIPG-XXXVI: 4000 cells/well; HSJD-DIPG-007: 6000 cells/well). The next day, drugs were added at the indicated concentrations, by adding 20 μl of a 5X concentrated stock, in TSM medium, to the cells. Four days later, relative cell growth or viability was assessed using the ATPLite kit (Perkin Elmer), according to the manufacturer's instruction. Luminescence was measured on a FlexStation 3 plate reader (Molecular Devices) with an integration time of 500 ms. Synergy analyses were performed using SynergyFinder (Ianevski et al., 2017).

NanoBRET Assays

HEK-293 cells were transfected in suspension at a density of 2 x 10^5 cells/ml. For 10 ml of HEK-293 cell suspension, a plasmid mixture of 500 ng of Acvr1-NanoLuc (Promega) and 4.5 μg of transfection carrier DNA (Promega) and 500 μl OPTI-MEM (Thermo Fisher) was prepared. 15 μl of FuGENE HD (Promega) was then added to the plasmid preparation and incubated for 20 minutes at room temperature. 500 μl of the transfection medium was added to 10 ml of HEK-293 cell suspension and the mixture was seeded into a T-75 flask. 24 hours after transfection, cells were trypsinised and re-suspended in phenol red-free OPTI-MEM (Thermo Fisher) at a density of 2 x 10^5 cells/ml. 17 μl of cell suspension was dispensed into each well of 384-well plate and mixed with 1 μl of 1.3 μM Tracer-6908 (Promega) diluted in 31.25% PEG-400 with 12.5 mM HEPES, pH7.5. Subsequently, 2 μl of E6201 dilutions in phenol red-free OPTI-MEM was added to achieve the concentrations indicated. Cells were agitated at 200rpm for 30 seconds and incubated for 2 hours at 37°C. BRET measurement was done using Intracellular TE Nano-Glo Substrate/Inhibitor reagents (Promega), following the manufacturer's instructions. Donor emission (450nm) and acceptor emission (610nm) were measured simultaneously on PHERAstar FSX microplate reader (BMG Labtech). BRET ratio values were calculated by dividing the acceptor emission by donor emission.

Reporter Assays

For reporter assays in primary glial cells, 5 x 10^4 primary glial cells were seeded in 6-wells plates in 2 mL growth medium per well. The next day, 1.5 x 10^6 PFU of the appropriate adenoviruses (Ad-GFP; Vector Biolabs #1060, or Ad-GFP-Cre, Vector Biolabs #1700) were

added to individual wells in 2 mL growth medium. The following day, transduction medium was removed, cells were washed with PBS, and 2 mL fresh growth medium were added in each well. For transfection, a plasmid mixture was prepared containing, for each well: 100 ng of *Renilla*-luciferase plasmid, 1 μg of 6XE2F-Luciferase plasmid (a gift of Dr Kristian Helin, University of Copenhagen), 125 μl OPTI-MEM (Thermo Fisher), and 2 μl P3000 reagent (Thermo Fisher). A mixture containing 125 μl OPTI-MEM and 2 μl Lipofectamine 3000 (Thermo Fisher) for each transfected well was then added to the plasmid preparation, and the samples incubated for 15 minutes at room temperature. 250 μl of the transfection medium was added to the appropriate wells. The next day, the transfection medium was replaced with 2 mL medium without serum. 24 hours later, cells were harvested, lysed and processed for measurement of luciferase activity using the Dual-Luciferase Reporter Assay System (Promega), following the manufacturer's instructions. Luminescence produced by *Renilla* and firefly luciferase activity were measured sequentially on a FlexStation 3 plate reader (Molecular Devices).

For *BRE-Luc* reporter assays in C2C12 and HEK-293 cells, cells were seeded in 24-well plates at a density of 5 x 10^4 (C2C12) or 1.5 x 10^5 (HEK-293) cells per well, and transfected with DharmaFECT Duo (Fisher Scientific) or PEI polyethylenimine (Polysciences), following the recommendations of the manufacturers. For ligand-stimulation experiments, cells were transfected with 150 ng *β-gal* control reporter and 300 ng *BRE-Luc* reporter per well. For experiments with receptor expression construct co-transfection, cells were transfected with 90 ng *β-gal* control reporter, 120 ng *BRE-Luc* reporter, and 200 ng receptor expression construct per well. Each transfection mixture was equalized with empty vector when necessary and every experiment was performed in triplicate. The *BRE-Luc* reporter construct (Korchynskyi and ten Dijke, 2002), the expression vectors encoding wild-type and R206H mutant ACVR1 (van Dinther et al., 2010), and the vectors encoding constitutively active (ca-) ACVR1, BMPR1A, and BMPR1B, have been previously described (Fujii et al., 1999). 48 hours after transfection, the cells were incubated with inhibitors and ligands at the concentrations and times indicated. Cells were harvested and lysed, and β-galactosidase and luciferase activity were measured using reporter assay reagents (Promega), following the manufacturer's instructions, on a Victor3 1420 luminometer (Perkin Elmer). Each transfection mixture was equalized with empty vector when necessary.

For *CAGA-Luc* experiments in HEK-293 cells, cells were seeded in 96-well plates at a density of 1 x 10^4 cells per well. For 100 wells, a plasmid mixture containing 500 ng *Renilla*-luciferase plasmid, 2 μg *CAGA-Luc* plasmid (a gift of Dr Petra Knaus, Free University of Berlin) and 250 μl OPTI-MEM (Thermo Fisher) was prepared. 7.5 μl of FugeneHD (Promega) was then added to the plasmid preparation and incubated for 20 minutes at room temperature. 2.5 μl of the transfection medium was added to each well. 24 hours after transfection, the cells were incubated with 10 ng/ml TGF-β and E6201 simultaneously at the concentrations indicated. 24 hours later, cells were harvested, lysed and processed for measurement of luciferase activity using the Dual-Luciferase Reporter Assay System (Promega), following the manufacturer's instructions. Luminescence produced by firefly and *Renilla* luciferase activity was measured sequentially on a PHERAstar FSX microplate reader (BMG Labtech).

X-Ray Crystallography

ACVR1 kinase domain (residues 172–499) and FKBP12 proteins were prepared from Sf9 insect cells and *E coli* strain BL21(DE3)R3-pRARE2, respectively, as described previously (Chaikuad et al., 2012). Proteins were initially purified by nickel affinity chromatography before mixing and subsequent purification by size exclusion chromatography on a Superdex 200 16/600 column. The eluted protein complex was stored in 50 mM HEPES pH 7.5, 300 mM NaCl, 10 mM DTT and the hexahistidine affinity tags cleaved using tobacco etch virus protease. Crystallization was achieved at 4°C using the sitting-drop vapor diffusion method. The ACVR1-FKBP12 complex at 12.5 mg/mL was preincubated with 1 mM E6201 and crystallized using a precipitant containing 0.05 M ammonium sulfate, 30% pentaerythritol ethoxylate 15/4, 0.1 M bis-Tris pH 6.5. Viable crystals were obtained when the protein solution was mixed with the reservoir solution at 2:1 volume ratio. Crystals were cryoprotected with mother liquor plus 25% ethylene glycol prior to vitrification in liquid nitrogen. Diffraction data were collected at the Diamond Light Source beamline I03 using monochromatic radiation at wavelength 0.9763 Å. Data were processed with MOSFLM (Leslie, 2006) and subsequently scaled using the program AIMLESS from the CCP4 suite (Winn et al., 2011). Initial phases were obtained by molecular replacement using the program PHASER (McCoy et al., 2007) and the structure of ACVR1 (PDB 3H9R) as a search model. The resulting structure solution was refined using Phenix Refine (Adams et al., 2010) and manually rebuilt with COOT (Emsley and Cowtan, 2004). Appropriate TLS restrained refinement using the tls tensor files calculated from the program TLSMD (Painter and Merritt, 2006) was applied at the final round of refinement. The complete structure was verified for geometric correctness with MolProbity (Davis et al., 2007). Data collection and refinement statistics are shown in Table S3.

Histology

Postnatal day 14 and 21 animals were deeply anesthetized using Avertin (250mg/kg, intraperitoneal), and transcardially perfused with PBS, and then with 4% paraformaldehyde (PFA). The brains were dissected, and post-fixed in 4% PFA overnight at 4°C. For postnatal day 7 animals, brains were dissected without perfusion, and fixed in 4% PFA overnight at 4°C. For frozen tissue sections, fixed brains were briefly rinsed in PBS, and cryoprotected by soaking in a 10% sucrose solution for 3 hours at 4°C, and then in a 30% sucrose solution overnight at 4°C. Cryoprotected brains were then embedded in Optimal Cutting Temperature (O.C.T.) compound (VWR), and 8 μm sections were cut on a CryoStar NX70 instrument (Thermo Scientific). O.C.T.-embedded patient tumor samples were obtained from the laboratory of Dr Michelle Monje. The patient characteristics and treatments received are described in (Nagaraja et al., 2019). For paraffin sections, fixed tissue samples were dehydrated, embedded in paraffin using a HistoCore Arcadia H instrument (Leica), and 4.5 μm sections were cut on a RM2255 instrument (Leica). Sections were mounted on +ASSURE®+ frosted slides (Epic Scientific).

For immunohistochemistry, paraffin tissue sections were re-hydrated through a series of xylenes and progressively more diluted ethanol series, ending in water. Endogenous peroxidase activity was neutralized by incubating the section with 3% H_2O_2 for 15 min at room temperature. Sections were washed three times in PBS for 5 minutes at room temperature. Antigen retrieval was performed by immersing slides in 10mM sodium citrate, pH 6.0, at over 100°C in a pressurized pressure cooker for 15 minutes. Sections were then washed in PBS for 5 minutes at room temperature, and blocked in PBST (PBS with 0.2% triton) supplemented with with 5% bovine serum albumin (BSA). Antibodies were applied on the sections at the indicated dilutions in PBST-BSA, and incubated overnight at 4°C. Sections were then washed three times in PBS for 5 minutes at room temperature, and secondary antibodies were applied on the sections at the indicated dilutions in PBST-BSA, and incubated for 1 hour at room temperature. Sections were washed three times in PBS for 5 minutes at room temperature, and incubated in ABC reagent mixture (VECTASTAIN Elite ABC HRP Kit, Vector Laboratories) for 30 minutes at room temperature. After a further three washes in PBS for 5 minutes at room temperature, sections were incubated in 100 mM Tris pH7.5 for 5 minutes at room temperature and briefly rinsed in H_2O. DAB HRP substrate solution (Vector Laboratories), prepared according to the manufacturer's instructions, was applied for 3 minutes, and the sections quickly rinsed in H_2O. Sections were counterstain with hematoxylin, dehydrated in a graded series of ethanol and xylenes, mounted with TBS (Cole-Parmer) and coversliped. Slides were scanned on a NanoZoomer 2.0 HT instrument.

For immunofluorescence, fixed frozen sections were briefly washed in PBS. Antigen retrieval, blocking and primary antibody incubation were performed as described above. Fluorophore-conjugated antibodies were diluted at the indicated concentrations in PBST-BSA, and applied to the sections for 1 hour at room temperature, protected from light. Sections were then washed three times in PBS for 5 minutes at room temperature with gentle motion, and incubated for 10 minutes at room temperature in 0.5 μg/mL DAPI. After a further three washes in PBS for 5 minutes at room temperature, sections were dehydrated in a graded series of ethanol and xylenes, mounted with Entellan (Sigma), and coverslipped.

Antibodies used were: goat anti-PDGFRA (mouse) (1:100, R&D #AF1062), rabbit anti-PDGFRA (human) (1:50; Cell Signaling #5241), rabbit anti-CNPase (1:100, Cell Signaling #D83E10), rat anti-Ki67 (1:175, Abcam # ab156956), rabbit anti-GFAP, (1:500, Dako/Agilent #Z0334), mouse anti-OLIG2 (1:500, Millipore #MABN50), mouse anti-Nestin (1:100, BD Pharmigen #556309), biotinylated rabbit anti-goat (1:500, Vector Laboratories #BA-5000), biotinylated rabbit anti-rat (1:500, Vector Laboratories # BA-4001), goat anti-Rabbit (1:500, Vector Laboratories #BA-1000), goat anti-mouse (1:500, Vector Laboratories BA-9200), and Cy5-conjugated donkey anti-rabbit (1:500 Jackson ImmunoResearch #711-175-152).

To detect endogenous tdTomato fluorescence, fixed frozen sections were briefly washed in PBS, and incubated for 10 minutes at room temperature in 0.5 μg/mL DAPI. Sections were then washed three times with PBS for 5 minutes at room temperature with gentle motion, and mounted with Vectashield medium (Vector Laboratories). Fluorescence images were acquired using a Zeiss AxioImager microscope fitted with a sCMOS camera, and ZEN software.

To quantify tdTomato$^+$ cells, images were processed in the ImageJ software (National Institutes of Health). Images were first converted to 16-bit format. For tdTomato fluorescence images, background was substracted using a rolling ball radius of 10 pixels. To restrict the fluorescence to nuclei, the image threshold was then adjusted on a dark background with a 30-70 range. Watershed segmentation was then performed within the binary processing functions of ImageJ to resolve cell doublets. Fluorescence positive cells were then counted using the Analyze Particles function, with a size (in inches^2) of 0.001 to infinity. The tissue area was measured (in arbitrary units) by tracing the contour of the tissue sections on the images with the polygon selection tool, and then using the "Measure" function. The number of tdTomato$^+$ cells was then normalized to the tissue area.

Chromatin Immunoprecipitation

Chromatin immunoprecipitation was performed as described (Kron et al., 2017), with some modifications: Chromatin was cross-linked by incubating cells in 1% formaldehyde for 10 minutes at 37°C. Cells were then washed once with PBS+0.5% bovine serum albumin (BSA), and collected in 500 μl PBS containing 1X protease and phosphatase inhibitors (Complete Mini and PhosphoSTOP, Roche) in 1.5 mL microtubes. Cells were pelleted by centrifugation at 300 x g for 5 minutes at 4°C. Samples were then lyzed in 350 μl Lysis Buffer (50 mM Tris-HCl pH8.1, 10 mM EDTA, 1% SDS, 1X protease and phosphatase inhibitors) by resuspending the cell pellet. Samples were sonicated using a Bioruptor Pico instrument (Diagenode) with 35 cycles of 30 seconds "on" and 30 seconds "off" at 4°C. Samples were then spun at 11 000 x g at 4°C, and the supernatant transferred to new microtubes. Twenty μl of the sample were kept aside as "input" material, and 300 μl added to 1500 μl Dilution Buffer (20 mM Tris-HCl pH8.1, 150 mM NaCl, 2 mM EDTA, 1% Triton X-100) in 2 mL microtubes. For immunoprecipitation, 100 μl of a bead-antibody mixture was added to each sample. These bead-antibody mixtures were prepared as follow: 10 μl Dynabeads A (Thermo Fisher) and 10 μl Dynabeads G per sample (Thermo Fisher) were washed 3 times in 500 μl PBS+0.5%BSA on a magnetic tube holder, incubated with 8 μg/sample H3-K27me3 (Millipore #07-449), 5 μg/sample H3-K27M (Millipore #ABE419), 0.5 μg/sample SUZ12 (Cell Signaling #3737), 4 μg/sample SMAD1 (Cell Signaling #6944) antibodies, or the equivalent amount of rabbit IgG, in 300 μl PBS+0.5%BSA with rotation of 6 hours at 4°C, washed twice with 500 μl PBS+0.5%BSA on a magnetic tube holder, and resuspended in 100 μl/sample in Dilution Buffer. Samples were rotated overnight at 4°C. The next day, beads were washed three times with 500 μl wash buffer (50 mM HEPES pH7.6, 1 mM EDTA, 0.7% sodium deoxycholate, 1% Nonidet P-40, 0.5 M lithium chloride), and twice with 750 μl 1X TE buffer (10 mM Tris-HCl pH8.0, 1 mM EDTA) on a magnetic tube holder. Beads and "input" samples were then resuspended in 100 μl decrosslinking buffer (1% SDS, 0.1 M sodium bicarbonate), and incubated for 24 hours at 65°C with constant motion. DNA was then isolated using the MinElute purification kit (Qiagen) following the manufacturer's instructions. Samples were assayed by qPCR using the primers listed in Table S4.

Flow Cytometry and Cell Sorting

For flow cytometry, cells suspensions were stained as appropriate in FACS buffer (PBS without $MgCl_2$ and $CaCl_2$, 1% FBS, 2 mM EDTA and 0.05% sodium azide) for 30 minutes on ice, washed twice with 2 mL cold FACS buffer with 5 minutes centrifugation at 4°C between each wash, and resuspended in FACS buffer before cell analysis on a Fortessa (BD Biosciences) instrument.

For lineage tracing and assessment of GFP-expressing DIPG cell lines, cell suspensions were stained with 2 μg/mL DAPI to mark dead cells. For cell sorting, GFP-expressing cells from DIPG cell lines were isolated using an FACSAria cell sorter (BD Biosciences), and collected in FACS buffer. Sorted cells were centrifuged at 300 x g for 5 minutes, resuspended in TSM medium, and plated.

For EdU incorporation measurements, cultured cells were incubated with 10 μM EdU for 2 hours at 37°C in a cell culture incubator, washed in PBS, dissociated as a single-cell suspension in 0.05% trypsin, washed with FACS buffer, and processed for EdU staining using the Click-iT EdU Alexa Fluor 647 Flow Cytometry kit (Thermo Fisher #C10424), following the manufacturer's instruction. 2 μg/mL DAPI was added to the Click-iT reaction to measure DNA content.

For PDGFRA cell surface staining, brainstem cells were dissociated as described for the preparation of primary brainstem glial cell cultures. The single-cell suspension was stained with 1:100 biotinylated anti-PDGFRA (Thermo Fisher #13-1401-82), followed by 1:500 Streptavidin-FITC (BD Biosciences #554060) and 2 μg/mL DAPI to mark dead cells.

For cell cycle assessment, cell suspensions were permeablized by incubating with 500 μl Cytofix/Cytoperm Buffer (BD Biosciences #554722), washed with 2 mL Perm/Wash Buffer (BD Biosciences #554723), and stained in Perm/Wash Buffer with 1:100 anti-Ki67-APC (Thermo Fisher #50-5698-82) and 2 μg/mL DAPI to measure DNA content.

For apoptosis assays, cells were collected in TrypLE and washed with 2 mL FACS buffer. Cells were pelleted by centrifugation at 300 x g for 5 minutes, and the liquid removed by aspiration. Cells were resuspended in 3 mL AnnexinV-binding buffer (10 mM HEPES pH 7.4, 150 mM NaCl, 2.5 mM $CaCl_2$ in PBS), pelleted by centrifugation at 300 x g for 5 minutes, and resuspended in AnnexinV-binding buffer containing 1:50 AnnexinV-FITC (BioLegend #640906) and 1:50 7-AAD (BioLegend #420404). Staining was performed for 30 minutes at room temperature, protected from light. Cells were washed twice with 3 mL FACS buffer, and analyzed by flow cytometry. Flow cytometry data were analyzed using FlowJo version 10.

QUANTIFICATION AND STATISTICAL ANALYSIS

Data were analyzed using t-tests, one- or two-way analysis of variance (ANOVA), Mantel-Cox, Gehan-Breslow-Wilcoxon, or linear regression, followed by multiple-comparisons tests (Bonferonni, Tukey or Sidak) where appropriate, using GraphPad Prism version 7.03. Throughout the manuscript, the following notation was used to indicate statistical significance: *:$p<0.05$; **:$p<0.01$; ***:$p<0.001$. All the error bars depict the standard error of the mean (SEM).

DATA AND CODE AVAILABILITY

The mouse RNA-sequencing data has been deposited in Gene Expression Omnibus, under accession number: GSE142776. The X-ray crystal structure of the ACVR1-FKBP12 complex bound to E6201 has been deposited in the Protein Data Bank, under identification code 6I1S. The human normal brain and DIPG tumor RNA-sequencing data supporting the current study have not been deposited in a public repository because the full analysis of this dataset has not yet been published. The data are available from Dr Cynthia Hawkins (The Hospital for Sick Children, Toronto, Canada) on request.

Cancer Cell, Volume *37*

Supplemental Information

Mutant *ACVR1* Arrests Glial Cell Differentiation to Drive Tumorigenesis in Pediatric Gliomas

Jerome Fortin, Ruxiao Tian, Ida Zarrabi, Graham Hill, Eleanor Williams, Gonzalo Sanchez-Duffhues, Midory Thorikay, Parameswaran Ramachandran, Robert Siddaway, Jong Fu Wong, Annette Wu, Lorraine N. Apuzzo, Jillian Haight, Annick You-Ten, Bryan E. Snow, Andrew Wakeham, David J. Goldhamer, Daniel Schramek, Alex N. Bullock, Peter ten Dijke, Cynthia Hawkins, and Tak W. Mak

A

Acvr1$^{floxG328V/+}$ X *EIIa-Cre*

Genotype	# alive after birth
Acvr1$^{+/+}$	19
Acvr1$^{floxG328V/+}$	17
Acvr1$^{+/+}$; *EIIa-Cre*	16
Acvr1$^{floxG328V/+}$; *EIIa-Cre*	0

B

Acvr1$^{floxG328V/+}$; *EIIa-Cre*

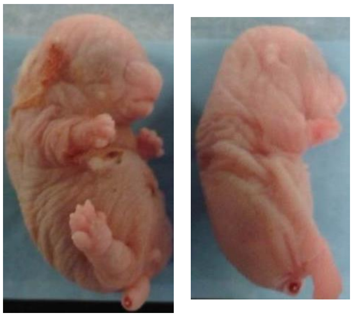

C

Postnatal day 6

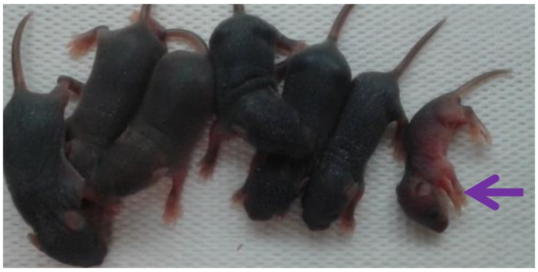

Figure S1, related to Figure 1. Characterization of the *Acvr1*^{floxG328V} allele and effects of the *Acvr1*^{G328V} mutation in glial cells

(A) Table showing the number of viable animals from various genotypes after birth, obtained from a cross of *Acvr1*$^{floxG328V/+}$ mice with *EIIa-Cre* mice.

(B) Photographs of two stillborn *Acvr1*$^{floxG328V/+}$*; EIIa-Cre* pups.

(C) Photograph of a litter from an *Acvr1*$^{floxG328V/+}$ X *Olig2*$^{Cre/+}$ intercross at postnatal day 6. The arrow indicates a runt *Acvr1*$^{floxG328V/+}$*;Olig2*$^{Cre/+}$ pup that did not survive until weaning.

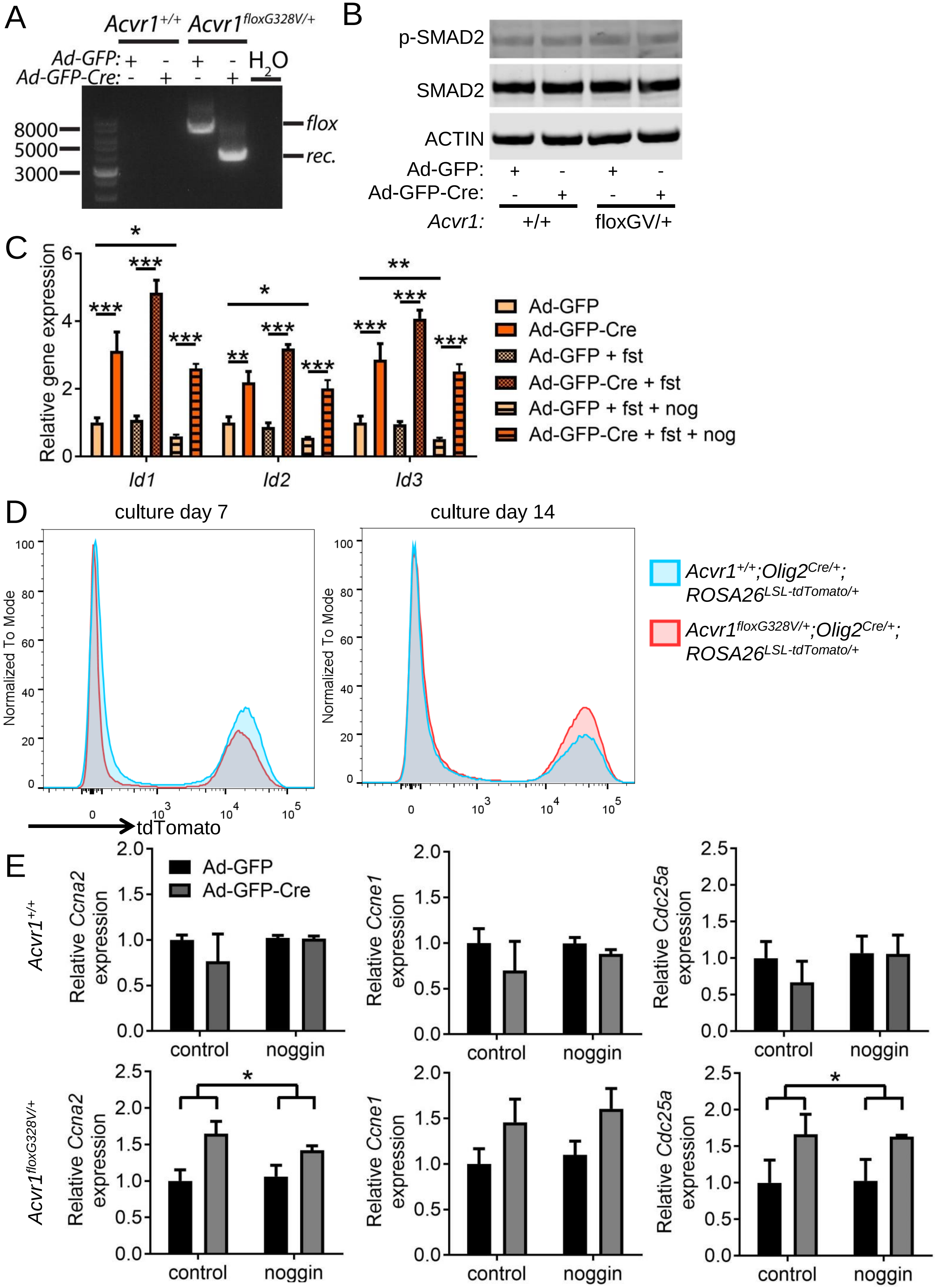

A
Acvr1+/+
Acvr1floxG328V/+
Ad-GFP:
Ad-GFP-Cre:
H2O
8000
5000
3000
flox
rec.
B
p-SMAD2
SMAD2
ACTIN
Ad-GFP:
Ad-GFP-Cre:
Acvr1:
+/+
floxGV/+
C
Relative gene expression
Id1
Id2
Id3
Ad-GFP
Ad-GFP-Cre
Ad-GFP + fst
Ad-GFP-Cre + fst
Ad-GFP + fst + nog
Ad-GFP-Cre + fst + nog
D
culture day 7
culture day 14
Normalized To Mode
tdTomato
Acvr1+/+;Olig2Cre/+;
ROSA26LSL-tdTomato/+
Acvr1floxG328V/+;Olig2Cre/+;
ROSA26LSL-tdTomato/+
E
Ad-GFP
Ad-GFP-Cre
Acvr1+/+
Acvr1floxG328V/+
Relative Ccna2 expression
Relative Ccne1 expression
Relative Cdc25a expression
control
noggin

F

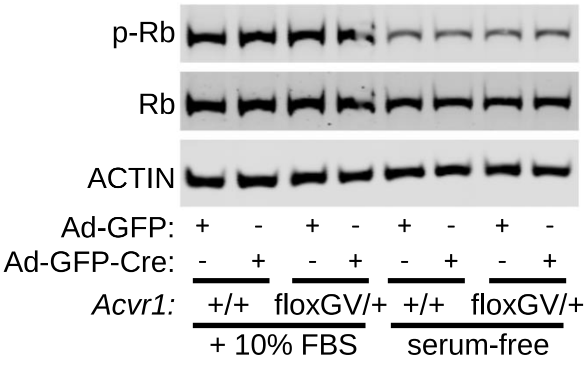

p-Rb
Rb
ACTIN
Ad-GFP: + - + - + - + -
Ad-GFP-Cre: - + - + - + - +
Acvr1: +/+ floxGV/+ +/+ floxGV/+
+ 10% FBS
serum-free

G

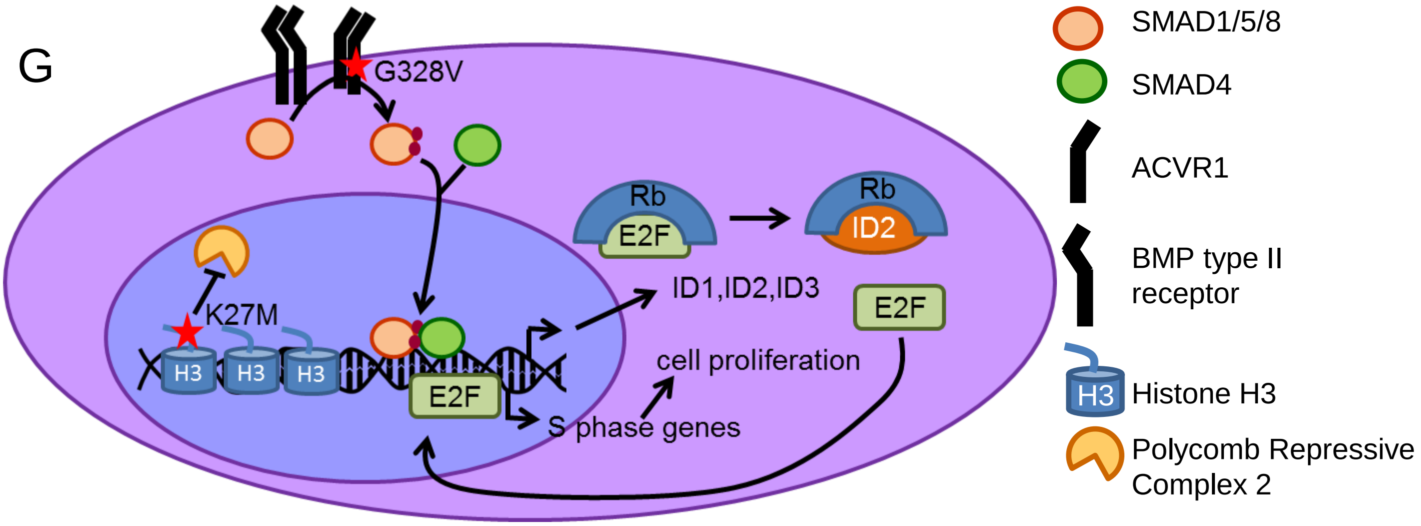

G328V
Rb
E2F
Rb
ID2
ID1,ID2,ID3
E2F
K27M
H3
H3
H3
E2F
cell proliferation
S phase genes
SMAD1/5/8
SMAD4
ACVR1
BMP type II receptor
H3
Histone H3
Polycomb Repressive Complex 2

Figure S2, related to Figure 2. ***Acvr1**^{G328V}* **enhances glial cell proliferation**

(A) Analysis of genomic DNA by PCR with primers amplifying the unrecombined (flox) and recombined (rec.) *Acvr1*$^{floxG328V/+}$ allele, extracted from primary brainstem glial cell cultures prepared from *Acvr1*$^{+/+}$ or *Acvr1*$^{floxG328V/+}$ pups and transduced with Ad-GFP or Ad-GFP-Cre. DNA markers are shown on the right lane.

(B) Western blot of whole cell lysates from primary glial cells prepared from *Acvr1*$^{+/+}$ or *Acvr1*$^{floxG328V/+}$ (floxGV) pups and transduced with Ad-GFP or Ad-GFP-Cre, probed with p-SMA2 (top), SMAD2 (middle), and actin (bottom) antibodies.

(C) Expression of *Id1*, *Id2*, and *Id3*, normalized to the expression of *Rpl19*, measured by qPCR on cDNA prepared from primary brainstem glial cells derived from *Acvr1*$^{floxG328V/+}$ pups, transduced with *Ad-GFP* or *Ad-GFP-Cre*, and treated or not with 100 ng/mL follistatin (fst) and/or 100 ng/mL noggin (Nog). n=4 independent cultures from 4 pups per genotype. *:$p<0.05$; **:$p<0.01$; ***:$p<0.001$, assessed by repeated-measures ANOVA, assessed by repeated-measures ANOVA with Sidak multiple comparisons tests.

(D) Flow cytometry histograms showing tdTomato-positive and tdTomato-negative cells in primary brainstem glial cell cultures prepared from *Acvr1*$^{+/+}$*;Olig2*$^{Cre/+}$*;ROSA26*$^{LSL\text{-}tdTomato/+}$ (blue trace) or *Acvr1*$^{floxG328V/+}$*;Olig2*$^{Cre/+}$*;ROSA26*$^{LSL\text{-}tdTomato/+}$ (red trace) pups at culture day 7 (left) and 14 (right). To adjust for different cell counts, data were normalized to the highest point on the histogram for in each sample (normalized to mode).

(E) Expression of *Ccna2* (left), *Ccne1* (middle) and *Cdc25a* (right), normalized to the expression of *Rpl19*, measured by qPCR on cDNA prepared from brainstem glial cultures derived from *Acvr1*$^{+/+}$ (top) or *Acvr1flox*$^{G328V/+}$ (bottom), and transduced with Ad-GFP (black bars) or Ad-GFP-Cre (grey bars). In all panels, *:$p<0.05$, assessed by repeated-measures ANOVA with Tukey multiple comparisons tests.

(F) Western blot of whole cell lysates from primary glial cells prepared from *Acvr1*$^{+/+}$ (+/+) or *Acvr1*$^{floxG328V/+}$ (floxGV) pups, transduced with Ad-GFP or Ad-GFP-Cre, and cultured in the presence of 10% fetal bovine serum (FBS) or under serum-free conditions, probed with phospho-Rb (p-Rb) (top), Rb (middle), and actin (bottom) antibodies.

(G) Model of the cellular effects of the *Acvr1*^{G328V} mutation on BMP target gene expression and cell proliferation, inferred from the data shown in this paper. *Acvr1*^{G328V} increases the expression of *Id1*, *Id2*, and *Id3*, and drive cell cycle progression in the absence of growth factors through a mechanism involving enhanced E2F-dependent transcriptional activity, likely as a result of increased ID2 levels.

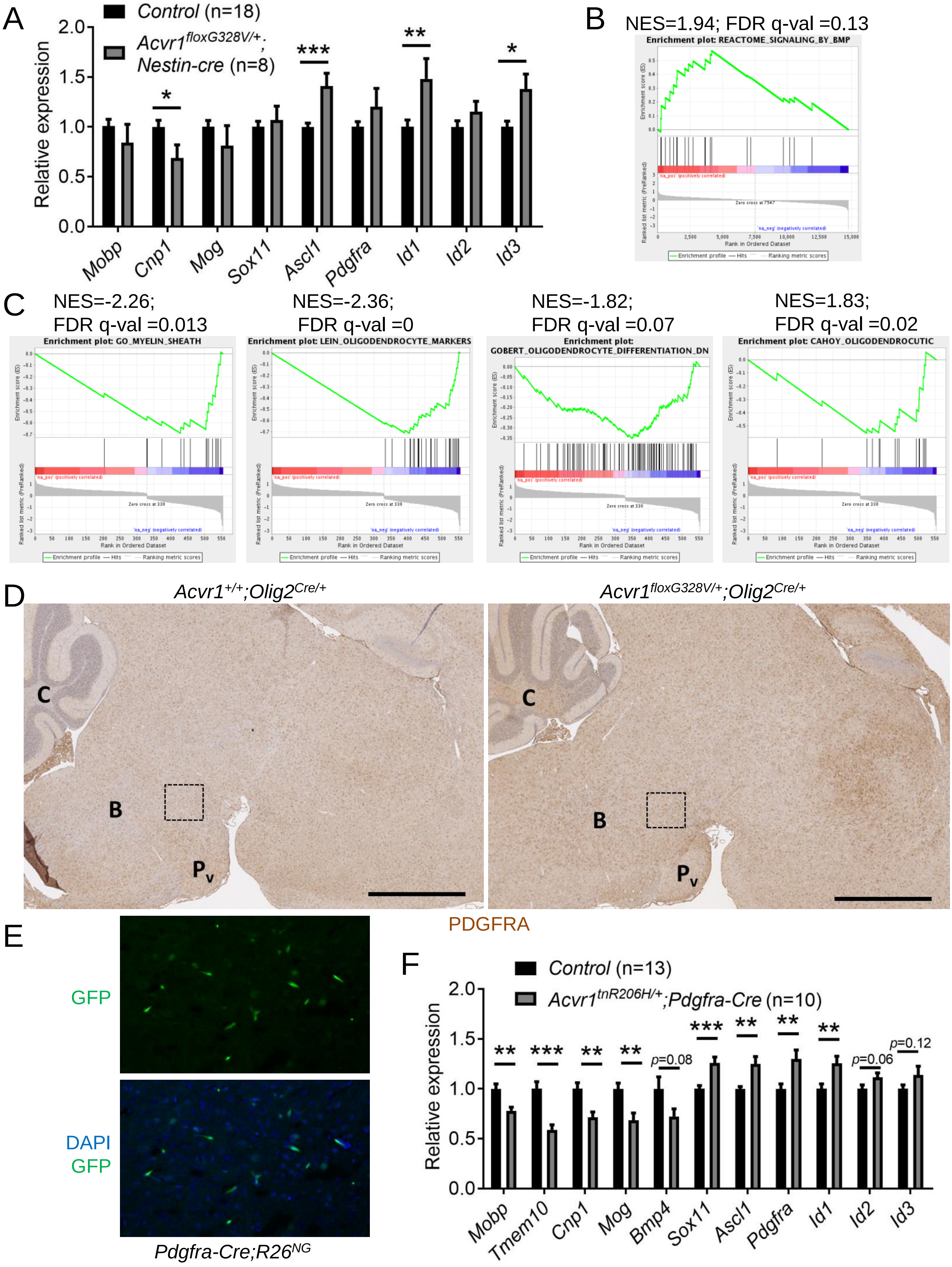

A
Control (n=18)
Acvr1floxG328V/+;
Nestin-cre (n=8)
Relative expression
2.0
1.5
1.0
0.5
0.0
*
***
**
*
Mobp
Cnp1
Mog
Sox11
Ascl1
Pdgfra
Id1
Id2
Id3
B
NES=1.94; FDR q-val =0.13
Enrichment plot: REACTOME_SIGNALING_BY_BMP
Zero cross at 7547
Rank in Ordered Dataset
C
NES=-2.26;
FDR q-val =0.013
Enrichment plot: GO_MYELIN_SHEATH
NES=-2.36;
FDR q-val =0
Enrichment plot: LEIN_OLIGODENDROCYTE_MARKERS
NES=-1.82;
FDR q-val =0.07
Enrichment plot:
GOBERT_OLIGODENDROCYTE_DIFFERENTIATION_DN
NES=1.83;
FDR q-val =0.02
Enrichment plot: CAHOY_OLIGODENDROCUTIC
D
Acvr1+/+;Olig2Cre/+
Acvr1floxG328V/+;Olig2Cre/+
C
B
Pv
C
B
Pv
PDGFRA
E
GFP
DAPI
GFP
Pdgfra-Cre;R26NG
F
Control (n=13)
Acvr1tnR206H/+;Pdgfra-Cre (n=10)
Relative expression
2.0
1.5
1.0
0.5
0.0
**
***
**
**
p=0.08
***
**
**
**
p=0.06
p=0.12
Mobp
Tmem10
Cnp1
Mog
Bmp4
Sox11
Ascl1
Pdgfra
Id1
Id2
Id3

Figure S3, related to Figure 3. ***Acvr1**^{G328V}* **induces differentiation arrest of oligodendroglial lineage cells and PDGFRA upregulation**

(A) Expression of selected genes, normalized to the expression of *Rpl19*, measured by qPCR on cDNA prepared from the brainstem tissue of control (black bars – a mix of *Acvr1*$^{+/+}$, *Acvr1*$^{floxG328V/+}$, and *Acvr1*$^{+/+}$*;Nestin-Cre*) and *Acvr1*$^{floxG328V/+}$*;Nestin-Cre* (grey bars) postnatal day 7 pups.

(B) Gene Set Enrichment Analysis (GSEA) showing significantly altered BMP signaling pathway (FDR q value <0.25) in *Acvr1*$^{floxG328V/+}$*;Olig2*$^{Cre/+}$ P7 pups, compared with their *Acvr1*$^{+/+}$*;Olig2*$^{Cre/+}$ littermates, using a pre-ranked list of all genes with FPKM>10. The Normalized Enrichment Score (NES) and FDR q value are indicated on top of the plot.

(C) Gene Set Enrichment Analysis (GSEA) showing significantly altered pathways (FDR q value <0.25), a pre-ranked list of genes differentially expressed in *Acvr1*$^{floxG328V/+}$*;Olig2*$^{Cre/+}$ P7 pups, compared with their *Acvr1*$^{+/+}$*;Olig2*$^{Cre/+}$ littermates, with adjusted p values lower than 0.1. The Normalized Enrichment Score (NES) and FDR q values are indicated on top of each plot.

(D) Representative immunohistochemistry images showing PDGFRA protein expression in sagittal brain sections from *Acvr1*$^{+/+}$*;Olig2*$^{Cre/+}$ (left) and *Acvr1*$^{floxG328V/+}$*;Olig2*$^{Cre/+}$ (right) postnatal day 14 mice. Higher magnifications of the boxed areas are shown in Figure 3D. C: Cerebellum. B: Brainstem. P_v: ventral pons. Scale bars: 1 mm.

(E) GFP (top) and combined GFP and DAPI (bottom) fluorescence microscopy images of a sagittal brainstem section for a postnatal day 7 *Pdgfra-Cre;R26*^{NG} mouse.

(F) Expression of selected genes, normalized to the expression of *Rpl19*, measured by qPCR on cDNA prepared from the brainstem tissue of control (black bars – a mix of *Acvr1*$^{+/+}$ and *Acvr1*$^{+/+}$*;Pdgfra-Cre*) and *Acvr1*$^{tnR206H/+}$*;Pdgfra-Cre* (grey bars) postnatal day 7 pups.

In (A) and (F), *:p<0.05; **:p<0.01; ***:p<0.001, assessed by unpaired t-test.

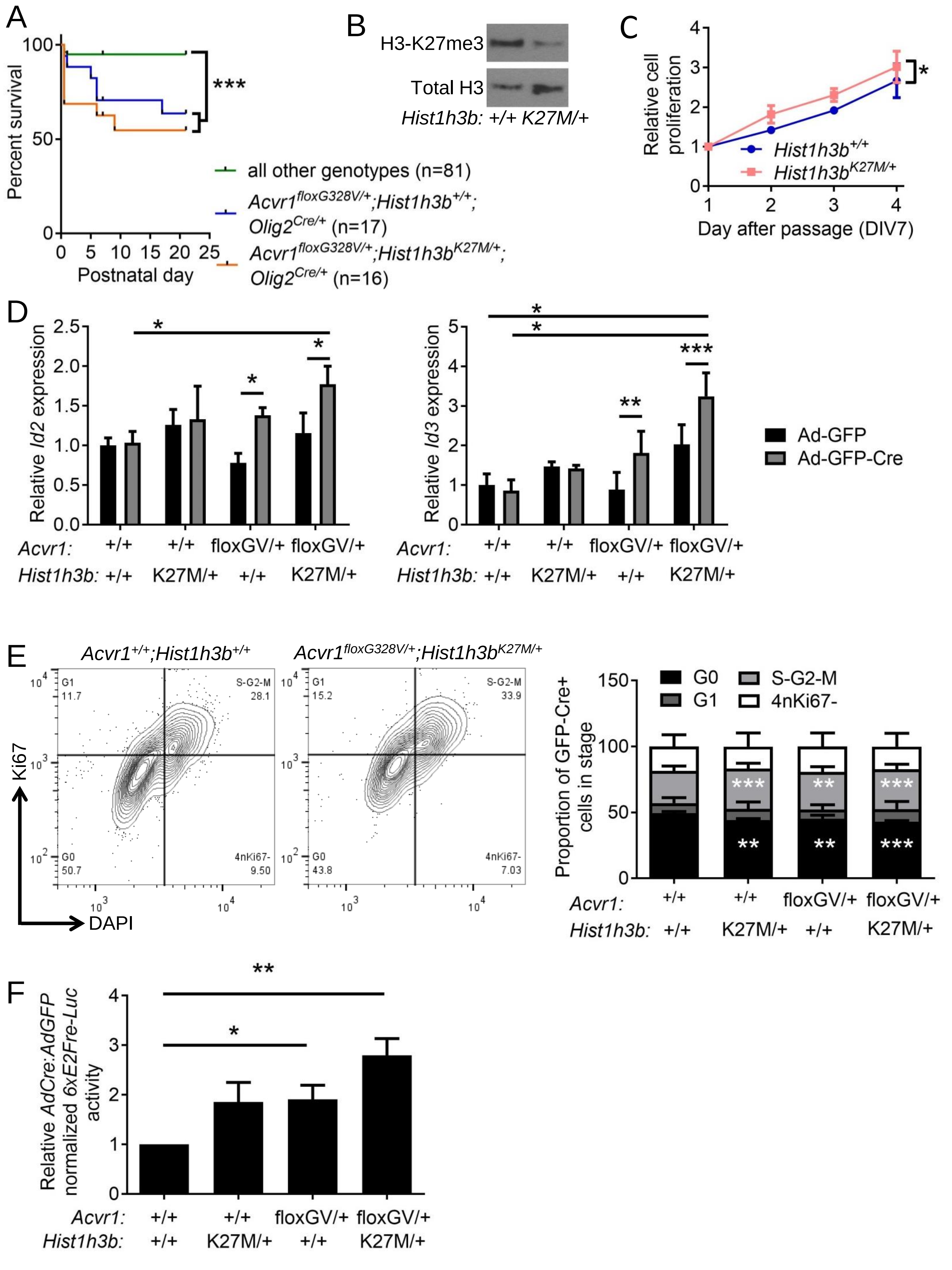

A
Percent survival
Postnatal day
***
all other genotypes (n=81)
Acvr1floxG328V/+;Hist1h3b+/+;Olig2Cre/+ (n=17)
Acvr1floxG328V/+;Hist1h3bK27M/+;Olig2Cre/+ (n=16)
B
H3-K27me3
Total H3
Hist1h3b: +/+ K27M/+
C
Relative cell proliferation
Day after passage (DIV7)
Hist1h3b+/+
Hist1h3bK27M/+
*
D
Relative Id2 expression
Relative Id3 expression
Ad-GFP
Ad-GFP-Cre
Acvr1: +/+ +/+ floxGV/+ floxGV/+
Hist1h3b: +/+ K27M/+ +/+ K27M/+
E
Acvr1+/+;Hist1h3b+/+
Acvr1floxG328V/+;Hist1h3bK27M/+
Ki67
DAPI
G1 11.7
S-G2-M 28.1
G0 50.7
4nKi67- 9.50
G1 15.2
S-G2-M 33.9
G0 43.8
4nKi67- 7.03
Proportion of GFP-Cre+ cells in stage
G0
G1
S-G2-M
4nKi67-
Acvr1: +/+ +/+ floxGV/+ floxGV/+
Hist1h3b: +/+ K27M/+ +/+ K27M/+
F
Relative AdCre:AdGFP normalized 6xE2Fre-Luc activity
Acvr1: +/+ +/+ floxGV/+ floxGV/+
Hist1h3b: +/+ K27M/+ +/+ K27M/+

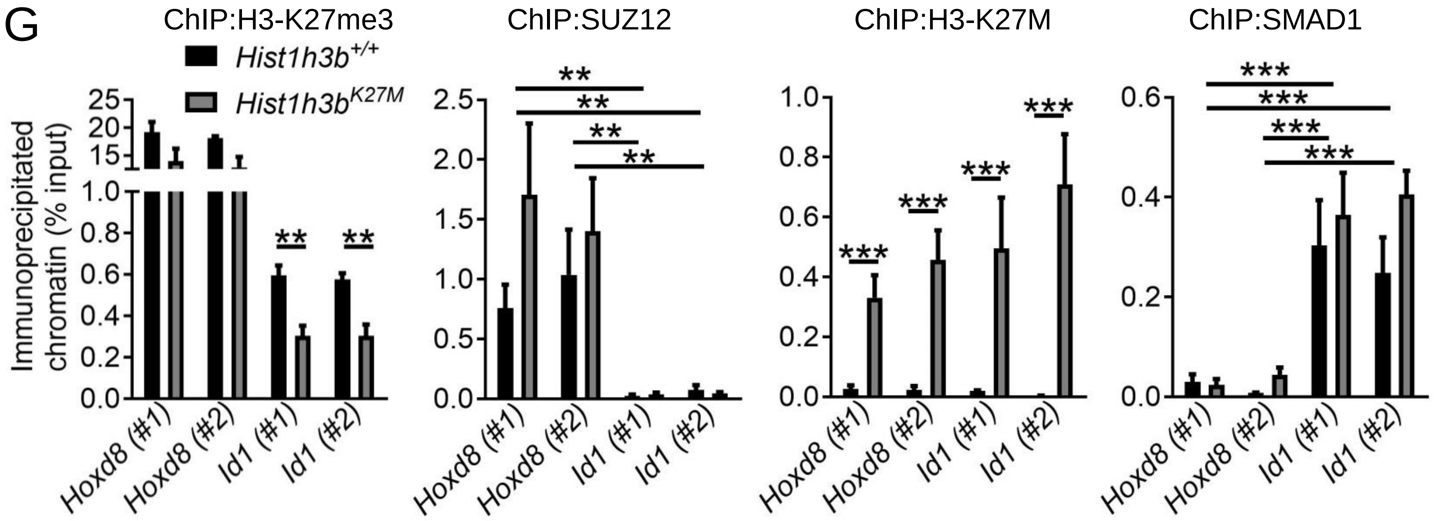
G
ChIP:H3-K27me3
ChIP:SUZ12
ChIP:H3-K27M
ChIP:SMAD1
Hist1h3b+/+
Hist1h3bK27M
Immunoprecipitated chromatin (% input)
Hoxd8 (#1)
Hoxd8 (#2)
Id1 (#1)
Id1 (#2)

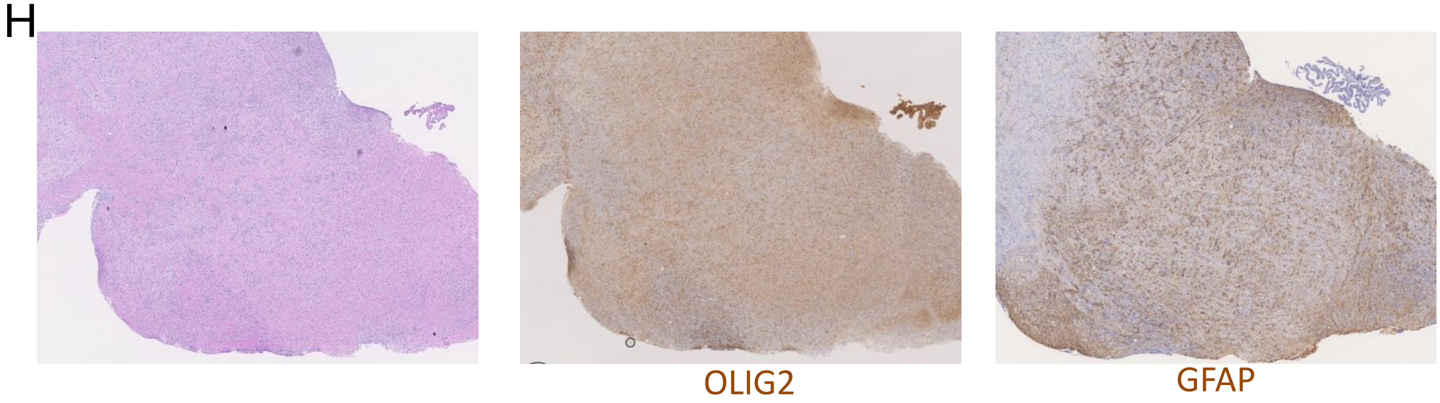
H
OLIG2
GFAP

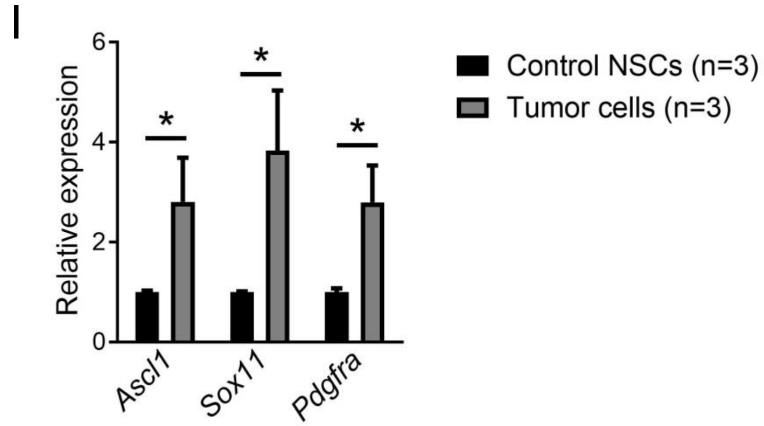
I
Relative expression
Control NSCs (n=3)
Tumor cells (n=3)
Ascl1
Sox11
Pdgfra

Figure S4, related to Figure 4. ***Acvr1*^{G328V} and *Hist1h3b*^{K27M} cooperatively stimulate gene expression and glial cell proliferation**

(A) Survival curves of *Acvr1*$^{floxG328V/+}$;*Hist1h3b*$^{K27M/+}$;*Olig2*$^{Cre/+}$ mice (orange curve), *Acvr1*$^{floxG328V/+}$;*Olig2*$^{Cre/+}$ mice (blue curve), and their control littermates (green curve), within the first three postnatal weeks.

(B) Western blot of whole cell lysates from primary brainstem glial cells prepared from *Hist1h3b*$^{+/+}$ or *Hist1h3b*^{K27M} pups, probed with H3-K27me3 (top), or total-H3 (bottom) antibodies.

(C) Relative cell proliferation, measured by the PrestoBlue assay, in primary brainstem glial cells prepared from *Hist1h3b*$^{+/+}$ (blue line) and *Hist1h3b*$^{K27M/+}$ (pink line) pups. n=5 independent experiments.

(D) Expression of *Id2* (left), and *Id3* (right), normalized to the expression of *Rpl19*, measured by qPCR on cDNA prepared from primary glial cells derived from pups carrying a combination of *Acvr1*$^{+/+}$ or *Acvr1*$^{floxG328V/+}$, and *Hist1h3b*$^{+/+}$ or *Hist1h3b*$^{K27M/+}$ alleles as indicated under the graphs (floxGV indicates the *Acvr1*^{floxG328V} allele), transduced with Ad-GFP (black bars) or Ad-GFP-Cre (grey bars), and treated with 100 ng/mL noggin. n=3 experiments from 3 independent litters.

(E) Left: representative flow cytometry plots depicting GFP-positive cells from Ad-GFP-Cre-transduced primary brainstem glial cell cultures prepared from *Acvr1*$^{+/+}$;*Hist1h3b*$^{+/+}$ and *Acvr1*$^{floxG328V/+}$;*Hist1h3b*$^{K27M/+}$ pups and stained with Ki67 antibodies and DAPI. The gating strategy to identify cells in the G0, G1, S-G2-M, and undefined ("4nKi67-") phases of the cell cycles is shown. Right: quantification of the percentage of GFP-positive cells in each cell cycle stage in Ad-GFP-Cre-transduced primary brainstem cell cultures derived from pups carrying a combination of *Acvr1*$^{+/+}$ or *Acvr1*$^{floxG328V/+}$, and *Hist1h3b*$^{+/+}$ or *Hist1h3b*$^{K27M/+}$ alleles as indicated under the graphs (floxGV indicates the *Acvr1*^{floxG328V} allele). n= 2 experiments from 2 independent litters.

(F) Relative reporter activity, measured by luminescence activity, in lysates from primary brainstem glial cells prepared from pups carrying a combination of *Acvr1*$^{+/+}$ or *Acvr1*$^{floxG328V/+}$, and *Hist1h3b*$^{+/+}$ or *Hist1h3b*$^{K27M/+}$ alleles as indicated under the graphs (floxGV indicates the *Acvr1*^{floxG328V} allele), transduced with Ad-GFP or Ad-GFP-Cre, and transfected with Renilla-Luc (to control for differences in transfection efficiency) and the 6xE2F-Luc reporter. Relative reporter activity was measured as a ratio of luminescence in lysates from cells transduced with Ad-GFP-Cre compared with Ad-GFP. n=4 experiments from 4 independent litters. Data from the "*Acvr1*$^{+/+}$;*Hist1h3b*$^{+/+}$" and "*Acvr1*$^{floxG328V/+}$;*Hist1h3b*$^{+/+}$" conditions are also shown by themselves in Figure 2F, and included here for comparison purposes.

(G) Occupancy of two regions of the *Hoxd8* promoter and of the *Id1* promoter by H3-K27me3 (left), SUZ12 (second from left), H3-K27M (third from left), and SMAD1 (right), measured by chromatin immunoprecipitation (ChIP) in primary glial cells prepared from *Hist1h3b*$^{+/+}$ (black bars) or *Hist1h3b*^{K27M} (grey bars) pups, expressed as a percentage of the input chromatin. For all the ChIP experiments, data were pooled from at least 3 independent primary glial cultures, prepared from separate animals, for each genotype.

(H) Histological sections showing an example of a high-grade diffuse glioma with brainstem involvement in an *Acvr1*$^{floxG328V/+}$*; Pik3ca*$^{floxH1047R/+}$*; Olig2*$^{Cre/+}$ mouse, stained with hematoxylin and eosin (left), and immunohistochemistry for OLIG2 (middle), and GFAP (right). Scale bars: 1mm.

(I) Expression of *Ascl1*, *Sox11*, and *Pdgfra*, normalized to the expression of *Rpl19*, measured by qPCR on cDNA prepared from cell lines derived from *Acvr1*^{floxG328V}; *Hist1h3b*^{K27M}; *Pik3ca*^{floxH1047R}; *Olig2*^{Cre} tumors, and from neural stem cells derived from normal brains, cultured in the same conditions.

In all panels, *:p<0.05; **:p<0.01; ***:p<0.001, assessed by Mantel-Cox test (A), linear regression analysis (C), repeated-measures ANOVA with Tukey (D-F) multiple-comparisons tests, two-way ANOVA with Sidak multiple comparisons tests (G), or t-tests (I).

A

	SOX11		*ASCL1*	
	log_2FC	Adj. p value	log_2FC	Adj. p value
Normal brain vs. DIPG	2	<0.0001	1.586	<0.0001
Normal brain vs. *ACVR1*-WT	2.006	<0.0001	1.489	0.0004
Normal brain vs. *ACVR1*-MUT	1.962	0.021	2.047	0.0002
ACVR1-WT vs. *ACVR1*-MUT	-0.044	>0.9999	0.558	0.4007

Spearman	*SOX11-ASCL1*	
	r	p value
Overall	0.807	8.93E-13
Normal	0.461	0.0625
DIPG	0.666	1.68E-05
ACVR1-WT	0.726	8.14E-06
ACVR1-Mut	0.7	0.233

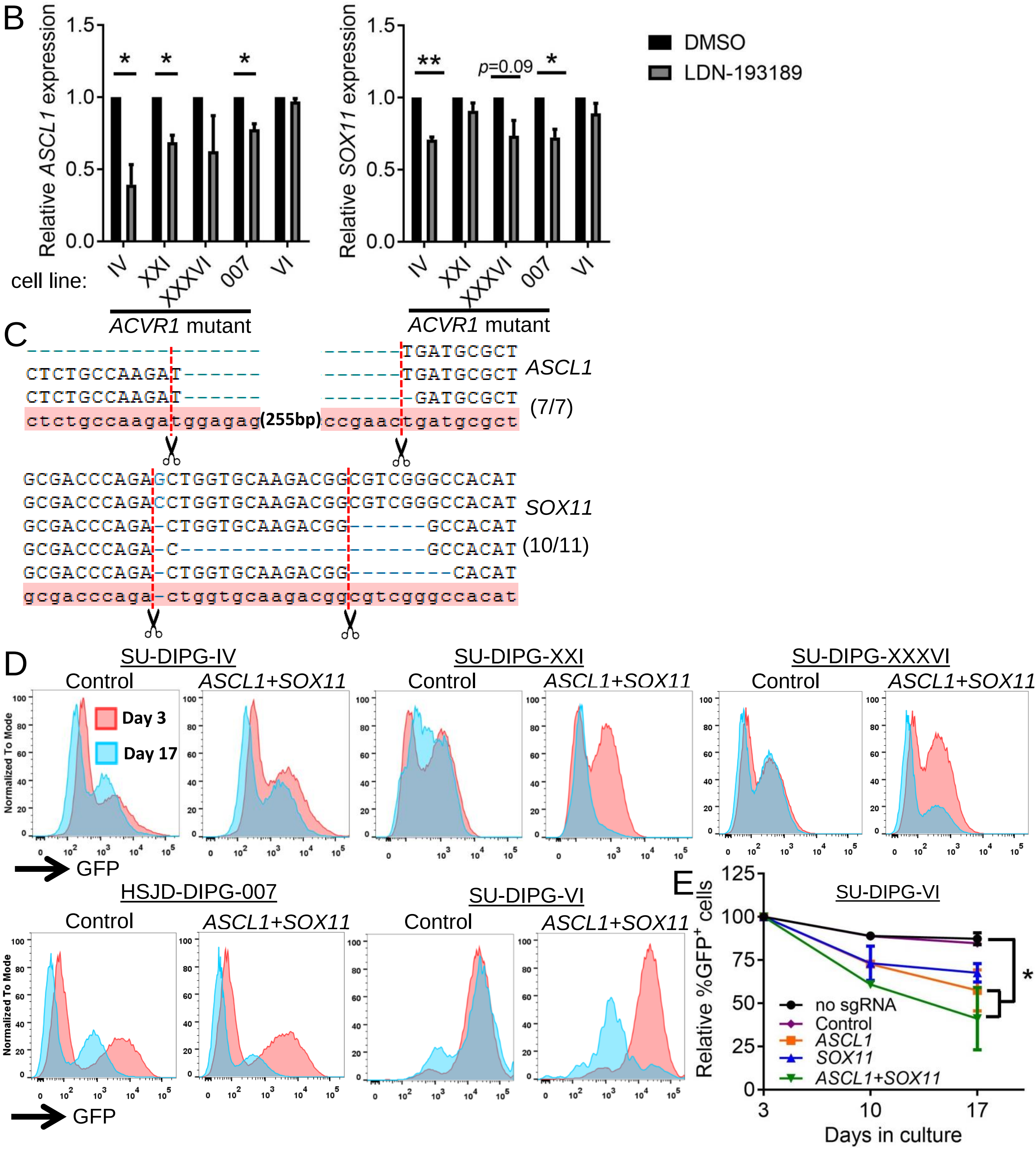

Figure S5, related to Figure 5. ASCL1 and SOX11 control DIPG cell fitness

(A) Left: summary statistic comparing the expression of *ASCL1* and *SOX11* in normal brain and DIPG samples, or only those DIPG samples that are wild-type (WT) or mutant (MUT) for *ACVR1*, assessed by ANOVA with Dunn's multiple comparisons tests. Right: Spearman analysis of the correlation between *ASCL1* and *SOX11* expression in the same samples.

(B) Expression of *ASCL1* (left) and SOX11 (right), normalized to the expression of *RPL19*, measured by qPCR on cDNA prepared from *ACVR1* mutant DIPG cell lines (SU-DIPG-IV, SU-DIPG-XXI, SU-DIPG-XXXVI, HSJD-DIPG-007, or *ACVR1* wild-type DIPG cells (SU-DIPG-VI), treated with 1 µM LDN-193189 (grey bars), or DMSO (black bars) for 24 hours.

(C) Alignment to the reference genome (highlighted in red) of sequencing reads from randomly selected clones generated from PCR-amplified fragments of the genomic regions edited by sgRNAs targeting *ASCL1* (top) and *SOX11* (bottom) in GFP-positive SU-DIPG-IV cells transduced with LentiCRISPRv2-GFP lentiviruses. Cells were transduced with a mixture of lentiviruses encoding two sgRNAs, and the scissors and dotted lines indicate the third nucleotide upstream of the protospacer adjacent motif (PAM) site for both gRNAs. The number of edited clones with insertions/deletions predicted to cause loss-of-function, out of the total number of sequenced clones, is indicated to the right.

(D) representative flow cytometry histograms showing GFP fluorescence for cells transduced with lentiviruses encoding control (left panels) or *ASCL1* and *SOX11*-targeting sgRNAs (right panels) at day 3 (red trace) and day 17 (blue trace) post-infection. To adjust for different cell counts, data were normalized to the highest point on the histogram for in each sample (normalized to mode).

(E) Relative percentage of GFP-positive cells over time in SU-DIPG-VI cells transduced with the lentiviruses encoding the indicated sgRNAs.

In all panels, *:$p<0.05$; **:$p<0.01$, assessed by *t*-test (B), or linear regression and slope comparisons (E).

Table S2, related to Figure 5. Expression of *ASCL1* and *SOX11* in normal brain tissue and DIPG tumor samples

Gene expression (FPKM)					
Normal brain		*ACVR1*-WT tumor		*ACVR1*-Mut tumor	
ASCL1	*SOX11*	*ASCL1*	*SOX11*	*ASCL1*	*SOX11*
7.75	4.2	8.62	18.55	31.19	41.06
1.23	2.12	26.62	23.28	5.34	0.99
2.05	1.69	3.15	3.91	17.39	7
1.1	1.34	16.42	11.16	31.12	9.68
2.25	2.71	5.82	7.21	17.85	56.03
2.46	2.49	12.04	39.07		
2.38	1.13	1.18	4.37		
2.24	1.38	8.14	11.12		
1.5	2.67	21.91	19.83		
1.86	1.89	1.56	1.87		
1.36	0.85	25.99	14.89		
1.55	1.78	1.71	3.05		
1.55	1.37	8.31	18.89		
2.09	2.11	11.08	9.56		
2.85	4.04	8.35	5.58		
1.83	1.41	15.89	18.51		
1.58	1.93	24.41	19.66		
		6.65	16.94		
		11.08	12.8		
		16.78	17.08		
		1.32	2.75		
		20.82	23.52		
		4.77	18.1		
		2.66	11.99		
		5.09	6.95		
		12.95	19.51		
		8.25	15.71		
		10.75	40.24		
		2.34	9.56		

Normalized expression of *ASCL1* and *SOX11* in normal brain tissue and *ACVR1*-WT or *ACVR1*-Mut DIPG tumor samples. Each row represents an individual patient. Normal brain: n=17; *ACVR1*-WT: n=29; *ACVR1*-Mut: n=5.

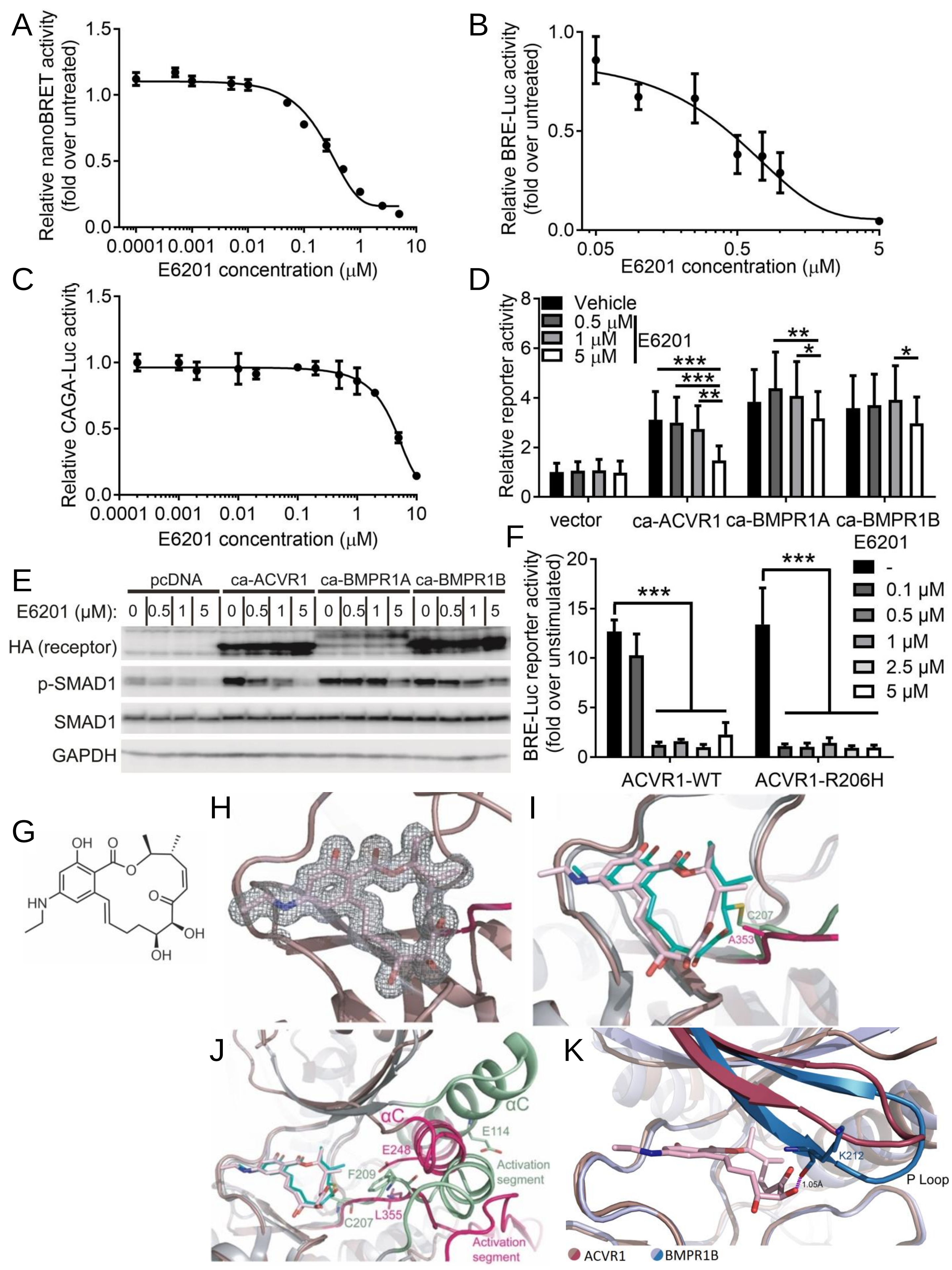

A
Relative nanoBRET activity (fold over untreated)
E6201 concentration (μM)
B
Relative BRE-Luc activity (fold over untreated)
E6201 concentration (μM)
C
Relative CAGA-Luc activity
E6201 concentration (μM)
D
Relative reporter activity
Vehicle
0.5 μM
1 μM
5 μM
E6201
vector
ca-ACVR1
ca-BMPR1A
ca-BMPR1B
E
pcDNA
ca-ACVR1
ca-BMPR1A
ca-BMPR1B
E6201 (μM):
HA (receptor)
p-SMAD1
SMAD1
GAPDH
F
BRE-Luc reporter activity (fold over unstimulated)
E6201
-
0.1 μM
0.5 μM
1 μM
2.5 μM
5 μM
ACVR1-WT
ACVR1-R206H
G
OH
O
HN
OH
H
I
C207
A353
J
αC
E114
E248
F209
C207
L355
Activation segment
K
K212
1.05A
P Loop
ACVR1
BMPR1B

L

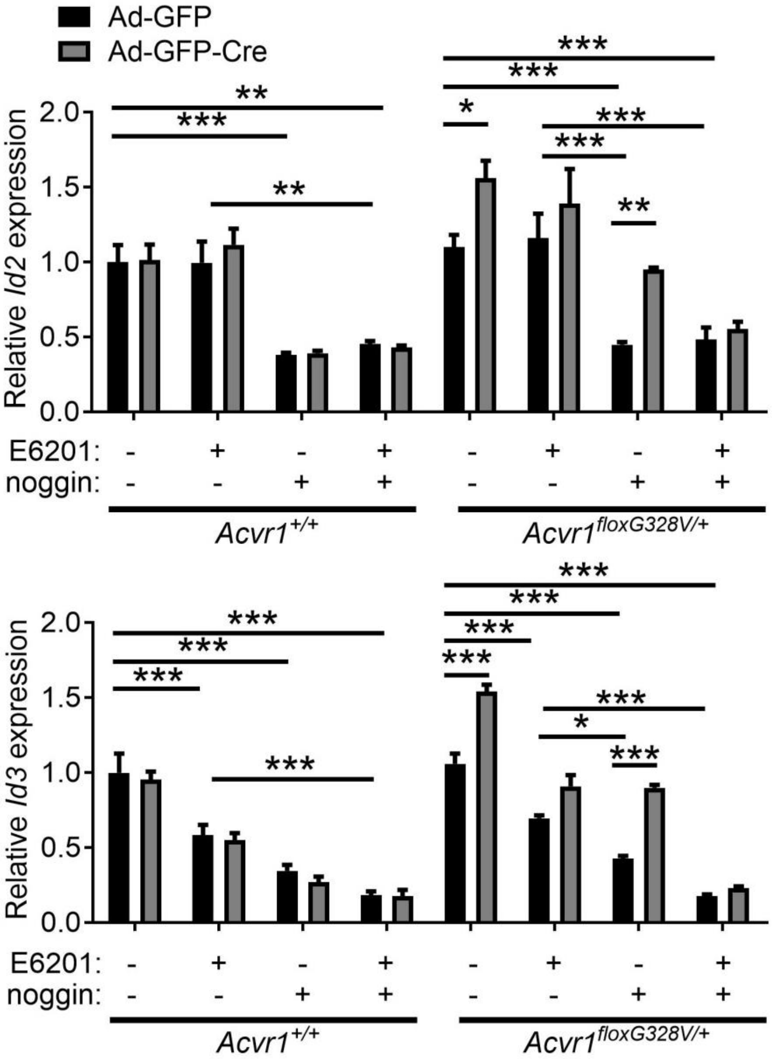

Ad-GFP
Ad-GFP-Cre
Relative Id2 expression
E6201:
noggin:
Acvr1+/+
Acvr1floxG328V/+
Relative Id3 expression
E6201:
noggin:
Acvr1+/+
Acvr1floxG328V/+

Figure S6, related to Figure 6. **E6201 inhibits BMP pathway activation induced by wild-type and mutant ACVR1**

(A) NanoBRET target engagement assay demonstrating binding of E6201 to ACVR1. Data shown are pooled from 3 independent experiments.

(B) Luminescent reporter activity in lysates from C2C12 cells transfected with BRE-Luc reporter and treated with 25 ng/mL BMP-6 and the indicated concentrations of E6201 overnight. Data shown are pooled from 3 independent experiments.

(C) Luminescent reporter activity in lysates from HEK-293 cells transfected with CAGA-Luc reporter and treated with 10 ng/ml TGF-β and the indicated concentrations of E6201 for 24 hours (IC_{50}~0.51 µM). Data shown are from triplicate measurements.

(D) Luminescent reporter activity in lysates from HEK-293 cells transfected with the BRE-Luc reporter and constructs encoding the indicated constitutively active (ca-) receptor, or empty vector, and exposed to the indicated concentrations of E6201 overnight. n= 3 independent experiments.

(E) Western blot of whole cell lysates from HEK-293 cell transfected with constructs encoding the indicated HA0-tagged constitutively active (ca-) receptor, or empty vector (pcDNA) probed with HA, p-SMAD1, SMAD1, and GAPDH antibodies. Point mutations for the receptors were: human ca-ACVR1: Q207D; human ca-BMPR1A: Q233D; mouse ca-BMPR1B: Q203D.

(F) Luminescent reporter activity in lysates from C2C12 cells transfected with the BRE-Luc reporter and constructs encoding wild-type (WT) or R206H mutant ACVR1, and exposed to 50 ng/mL BMP6 and the indicated concentrations of E6201 overnight. Data are shown as fold over luminescence activity in untreated cells transfected with wild-type (WT) or R206H mutant ACVR1. n= 3 experiments.

(G) Chemical structure of E6201.

(H) 2Fo-Fc electron density map for E6201 in the ACVR1 complex contoured at 1 σ.

(I) Superposition of the E6201 complexes of ACVR1 (brown ACVR1 ribbon, pink E6201) and MEK1 (grey MEK1 ribbon, green E6201; PDB 5HZE). MEK1 shows covalent binding to E6201 through Cys207, which is replaced by Ala353 in ACVR1.

(J) MEK1 complex shows an αC out, DFG-out conformation in contrast to the αC in, DFG-in conformation of ACVR1. Parts of strands β1 and β2 are omitted for clarity.

(K) Superposition of the ACVR1-E6201 structure (red) with that of the BMPR1B-LDN-193189 inhibitor complex (blue, PDB 3MDY). ACVR1 and BMPR1B adopt distinct inactive conformations of the kinase domain. The BMPR1B structure suggests it favours a more collapsed conformation of the P-loop (β1-β2) that would clash with E6201 (purple dashed line). While no structure exists for the kinase domain of BMPR1A, it is likely to resemble BMPR1B with which it shares 85% sequence identity compared to just 64% for ACVR1.

(L) Expression of *Id2* (top), and *Id3* (bottom), normalized to the expression of *Rpl19*, measured by qPCR on cDNA prepared from primary brainstem glial cells derived from *Acvr1*$^{+/+}$ or *Acvr1flox*$^{G328V/+}$ pups, transduced with Ad-GFP (black bars) or Ad-GFP-Cre (grey bars), and treated where indicated with 100 ng/mL Noggin and/or 1 µM E6201. n=3 experiments from 3 independent litters.

In all panels, *:p<0.05; **:p<0.01; ***:p<0.001, assessed by repeated-measures ANOVA with Tukey multiple comparisons tests.

Table S3, related to Figure 6. X-ray crystallography data collection and refinement statistics.

	ACVR1-FKBP12 complex with E6201
Wavelength	0.9763
Resolution range [Å]	41.32 - 1.52 (1.574 - 1.52)
Space group	$P2_12_12_1$
Cell dimensions [Å] α,β,γ	*a* = 44.3, *b* = 108.6, *c* = 114.4 90°, 90°, 90°
Total reflections	2436319 (240741)
Unique reflections	85798 (8479)
Multiplicity	28.4 (28.4)
Completeness (%)	99.95 (99.99)
Mean *I/σI*	33.17 (1.95)
R-merge	0.712 (2.274)
R-meas	0.727 (2.319)
R-pim	0.143 (0.448)
CC1/2	0.841 (0.581)
CC*	0.956 (0.857)
Reflections (for R-free)	4191 (386)
R-work (%)	17.9 (23.1)
R-free (%)	19.4 (24.8)
# Atoms total	3762
macromolecules	3464
Ligands	61
solvent	237
RMSD bonds [Å]	0.006
RMSD angles [°]	1.18
Ramachandran favored (%)	98.11
Ramachandran allowed (%)	1.89
Average B-factor [$Å^2$]	23.00
Macromolecules [$Å^2$]	22.49
Ligands [$Å^2$]	27.90
Solvent [$Å^2$]	29.14
Number of TLS groups	10

Statistics for the highest-resolution shell are shown in parentheses.

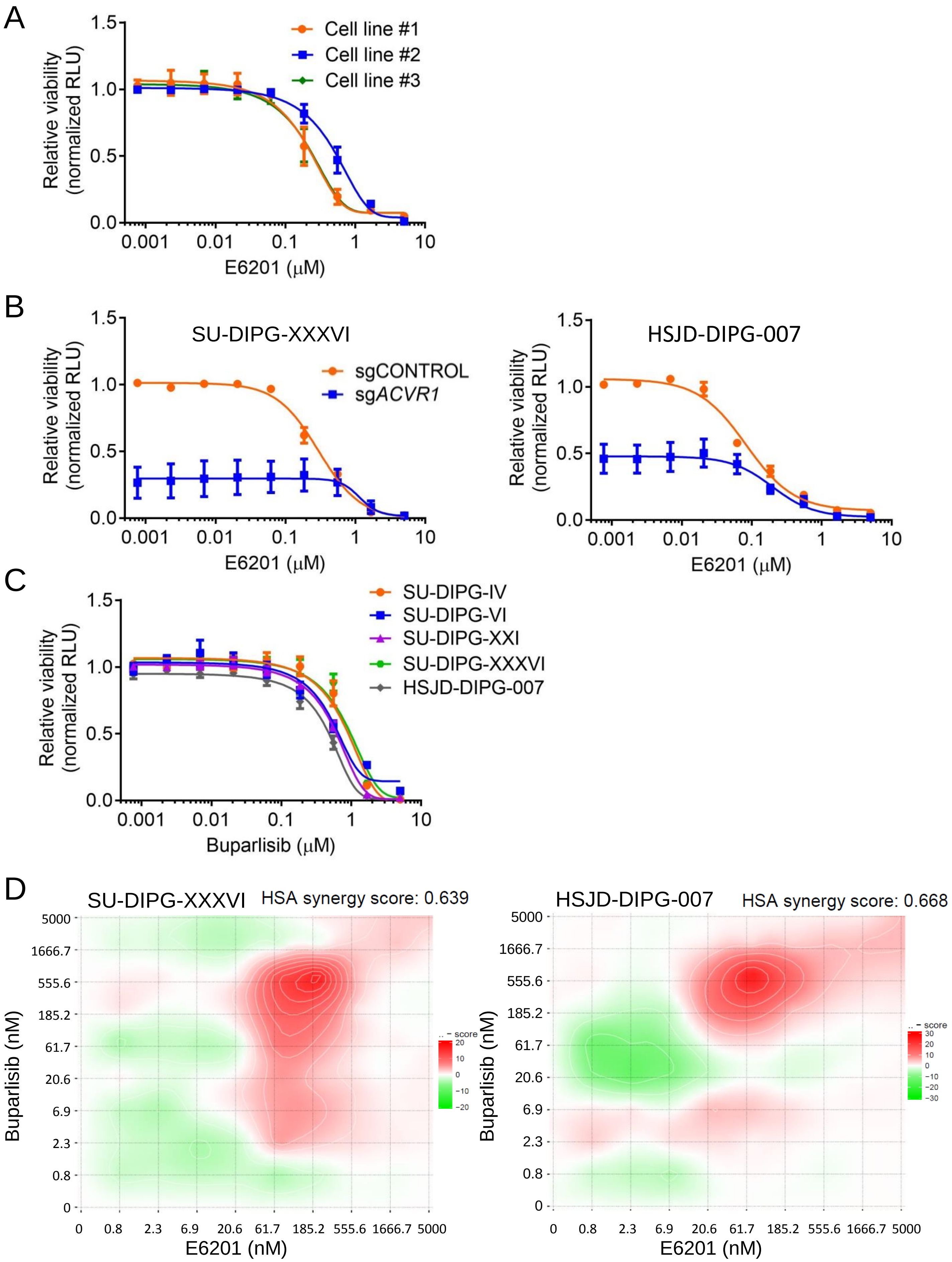
A
Cell line #1
Cell line #2
Cell line #3
Relative viability (normalized RLU)
E6201 (μM)
B
SU-DIPG-XXXVI
sgCONTROL
sgACVR1
HSJD-DIPG-007
C
SU-DIPG-IV
SU-DIPG-VI
SU-DIPG-XXI
SU-DIPG-XXXVI
HSJD-DIPG-007
Buparlisib (μM)
D
SU-DIPG-XXXVI HSA synergy score: 0.639
HSJD-DIPG-007 HSA synergy score: 0.668
Buparlisib (nM)
E6201 (nM)
score

Figure S7, related to Figure 7. **Characterization of E6201 as an inhibitor of mutant ACVR1**

(A) Relative cell proliferation or viability, measured by the ATPLite ATP-dependent luminescence reaction, in cell lines derived from three *Acvr1*$^{floxG328V/+}$*; Hist1h3b*$^{K27M/+}$*; Pik3ca*$^{floxH1047R/+}$*; Olig2*$^{Cre/+}$ brain tumors, exposed to increasing concentrations of E6201. n=3 experiments for each cell line.

(B) Relative cell proliferation or viability, measured by the ATPLite ATP-dependent luminescence reaction, in SU-DIPG-XXXVI (left) or HSJD-DIPG-007 (right) cells transduced with LentiCRISPRv2-GFP lentiviruses encoding a control (orange line) or *ACVR1*-targeting sgRNAs (blue line), and exposed to increasing concentrations of E6201. Data were normalized to the vehicle-treated condition for *sgCONTROL*. n= 3 experiments for each cell line.

(C) Relative cell proliferation or viability, measured by the ATPLite ATP-dependent luminescence reaction, in DIPG cell lines exposed to increasing concentrations of Buparlisib. n=3 experiments for each cell line.

(D) Visualization of Highest Single Agent (HAS) synergy scores in SU-DIPG-XXXVI (left) or HSJD-DIPG-007 (right) cells exposed to various concentrations of E6201 and/or Buparlisib, based on measurements of ATPLite ATP-dependent luminescence reactions. Score calculations and graphical representation were generated using the SynergyFinder software. Areas of potential synergy are indicated in red; areas of potential antagonism are indicated in green.
